# Early life famine exposure and anthropometric profile in adulthood: a systematic review and Meta-analysis

**DOI:** 10.1186/s40795-022-00523-w

**Published:** 2022-04-22

**Authors:** Getachew Arage, Tefera Belachew, Kalkidan Hassen Abate

**Affiliations:** 1 Department of Nutrition and Dietetics, College of Health Sciences, Debre Tabor University, Debre Tabor, Ethiopia; 2 Department of Nutrition and Dietetics, Institute of Health, Jimma University, Jimma, Ethiopia

**Keywords:** Anthropometric profile, Famine exposure, Meta-analysis, Systematic review

## Abstract

**Background:**

Previous famine studies reported the association between early life famine exposure and adulthood anthropometric profile. However, the findings were variable. Thus, a systematic review and meta-analysis was conducted to clarify the association of famine exposure in early life with the anthropometric profiles in adults.

**Methods:**

Potentially relevant studies were searched through Scopus, Medline, Google Scholar and Google for gray literature and reference lists of previous studies. The random effects model (REM) and I^2^ test was used to adapt the pooling method and assess heterogeneity, respectively.

**Results:**

Prenatal famine exposure was associated with increased risk of body mass index [SMD = 0.10 (95% CI: 0.02, 0.18)], waist circumference [SMD = 0.21 (95% CI: 0.11, 0.31)] in adults. Likewise, famine exposure during prenatal life was associated with decreased adult height [SMD) = − 0.26 (95% CI: − 0.44, − 0.09)]. Moreover, famine exposure during early childhood was associated with increased risk of waist circumference [SMD = 0.09 (95% CI: 0.01, 0.16)] and decreased adult height [SMD = − 0.16 (95% CI: − 0.27, − 0.04)].

**Conclusion:**

Our finding indicates that exposure to famine during early life was associated with the anthropometric profile of adults. In terms of public health significance, the results of the study further underscore the importance of improving the nutritional status of mothers and children to prevent adulthood diseases in the long run.

**Systematic review registration number:**

PROSPERO CRD42020168424

**Supplementary Information:**

The online version contains supplementary material available at 10.1186/s40795-022-00523-w.

## Background

According to the Developmental Origins of Health and Disease (DOHaD) hypothesis nutritional deprivation during the critical periods of growth and development leads to structural and functional changes and increases the risk of developing adulthood disease later in life [1–6]. Early life, particularly intrauterine, first 2 years of postnatal and adolescence stage of life are the critical “window” period for for all rounded development of human capital, where optimal nutrition during this period is fundamental [7, 8]. These periods are exceptional periods where the body employs reductive adaptive mechanisms to sustain life at the expense of shaping the future adulthood for the worst [9, 10].

Naturally, growth and development is determined by our genome, but realization of this growth potential is only possible if nutrient supplies are maintained to the fullest, especially during the critical periods of life [11]. However, when these supplies are restricted, physiological adaptive process takes place to ensure survival, may leave behind a permanent damage of the exposure [2, 4, 12]. For example, the pancreas is fully formed by the time of birth and while the number of islets is set in utero [13]. Subtle developmental exposures that resulted in fewer islets formation may have no immediate impact upon pancreatic function but may mark pancreatic insufficiency in response to aging [14]. The kidney as well, may reflect ill function during adulthood due to impact of fetal adversaries on nephrogenesis [15].

In order to generate the best available evidence on the long-term impact of early life famine exposure on adulthood health, a natural study setting is required where the exposure was a natural phenomenon, such as famine. Famine studies can serve as a natural experimental setting which can provide unique insights into the effect of earl life undernutrition on the development adulthood diseases [16–18]. In light of an impending food insecurity today the famine studies are a compelling event from which to learn, look back, and look forward to preventing famine-related adverse outcomes [19, 20]. Moreover, the long-term anthropometric consequences of early-life undernutrition is very interesting and important to stimulate new thinking in the concepts of health and diseases [21, 22].

Previous famine studies have provided a number of evidences supporting the association between early famine exposure and adulthood anthropometric profile [23–35]. Yet, the findings were not always consistent. For example, positive associations of early life famine exposure with obesity [23, 27, 34] and adult height [26, 31, 35] were found in China and Dutch famine studies, but not in Leningrad studies [30, 36] and another Chinese study [35]. Two previous systematic review and meta-analysis reported the association between early life famine and the risks of obesity and overweight later in life [37, 38]. However, the potential impact of early life exposure to famine on height, BMI, and waist circumference has not been quantified. Given these considerations, we will conduct a systematic review and meta-analysis of observational studies in order to gain a better understanding of the links between early life famine experience and adult anthropometric measurements.

## Methods

### Search strategy and study selection

*This review looked at both published and unpublished research to assess if there was a link between early life famine experience and anthropometric measurements in adults. Manual and electronic searches were used to locate the studies. The following databases were used to conduct an electronic search. An electronic search was conducted on* Scopus, Medline, and Google Scholar databases. Gray literatures were retrieved using Google. Moreover, a manual search was performed to locate papers from previous studies. *The Preferred Reporting Items of Systematic Review and Meta-Analysis (PRISMA) 2020 guideline was used* [39]*, and the following keywords were used to search the articles:* “Famine” OR “*malnutrition*” *OR* “*undernutrition*” *OR* “malnourishment” OR *“starvation*” OR “hunger” *AND “early life” OR “pregnancy*” *OR* “*fetus*” *OR* “*infant*” *OR* “child” or “adolescence” *AND* “*height*” *OR “short stature” OR “body mass index*” OR waist circumference” OR “waist to height ratio” *AND* “*Adults*”.

The research question was defined by the Participants, Interventions, Comparisons, Outcomes, and Study design (PICOS) criteria*. In order to avoid double counting, only the article with the most relevant was included if several articles reported data from the same study population.*

### Inclusion and exclusion criteria’s

Both published and unpublished observational studies conducted among early life famine exposed adults (aged ≥19 years) in any setting across the world were included. *The study included all articles published until October 30, 2020.* The studies that did not fully accessed after accessing abstracts were excluded after at least two email contacts with the primary author. The exclusion of these articles reflects our inability to assess the quality of articles in the absence of full text. Furthermore, articles written in languages other than English were excluded.

### Data extraction process

A standardized data extraction format was used to abstract data from the included articles **(Supplementary file** **1**), which was adapted from the Johanna Briggs Institute’s data extraction format [40]. All relevant data for this review were extracted by two reviewers (GA and KHA). The disparities between to reviewers at the time of data abstraction were resolved through discussion. The corresponding author of the original research was contacted via email for further information or to clarify procedure details. The data extraction format included first author, study design, age sample size, publication year, country of origin, outcome, main findings between famine exposure in early life and adulthood.

### Measurement of outcome variables

*The primary interest of the study was to investigate the association of early life famine exposure and adulthood anthropometric measurements such as height, body mass index and waist circumference* ***.***

### Measurement of exposure

This review was considered studies that report on the association between early life famine exposure (prenatal, early childhood, mid-childhood, adolescence) and adulthood anthropometric measurements. Prenatal exposure was defined as exposure to famine in during pregnancy, early childhood exposure was defined as exposure to famine during the first 2 years of life after birth, mid-childhood exposure was defined as exposure to famine during 4–9 years of age, adolescence exposure was defined as exposure to famine during 10–19 years of age [41].

### Quality assessment

Two authors (GA and KHA) independently assessed the quality of each original study using the Newcastle Ottawa Scale, a three-part approach, for observational studies quality assessment [42] There are three key parts to the tool. The first component, which was assessed on a scale of one to five stars, was primarily concerned with the methodological quality of each piece. The second component assesses the study’s comparability, with a possible two-star rating. The third component, which was graded from three stars, focused on the outcomes and statistical analysis of each original research. Disagreements between the two reviewers were solved through discussion. Articles with a scale of ≥6 from 10 scales were categorized as high quality (**Supplementary file** **2****).**

### Statistical analysis

Statistical analysis was performed using the Rev. Man 5.3 software (Rev Man 5.3) [43]. Odds Ratio (OR) pooled with 95% CI was determined to assess the strength of the association between exposure to famine and the risk of overweight, general obesity, and abdominal obesity. Standardized mean difference (SMD) was used to compare BMI, waist circumference, and height difference between exposed and nonexposed groups. The I square value (I2) was used to assess the heterogeneity between studies and the Random Effects Model (REM) was used as a pooling method. The I^2^ values of 0, 25, 50 and 75%, respectively, represent no, low, moderate, and high heterogeneity, while the *P*-values of chi-square statistics < 0:05 represent significant heterogeneity. Sensitivity analysis was performed by sequential failure of individual studies to further evaluate the source of heterogeneity [44]. Subgroup analyzes were conducted on the basis of gender. Publication bias was assessed through funnel plots.

## Results

### Characteristics of the study

**Fig. 1:**
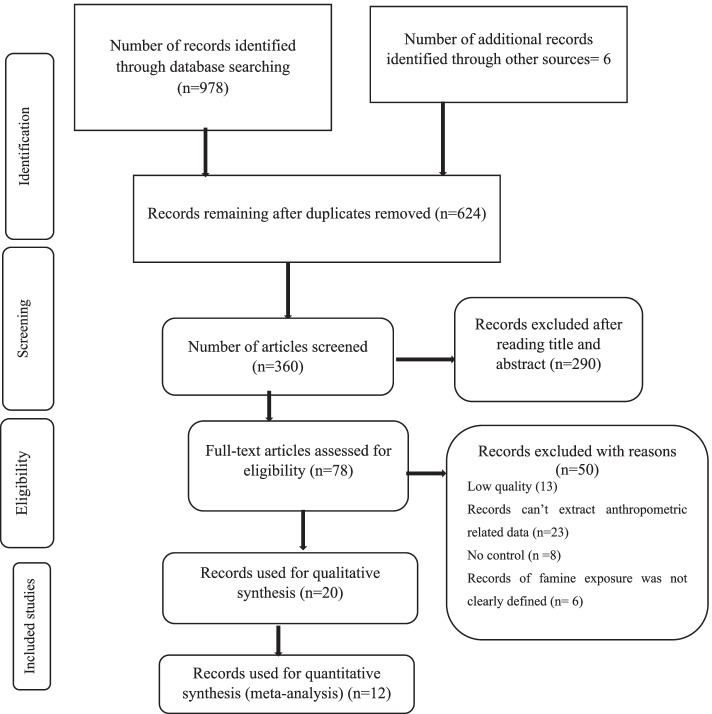
*Flow diagram of studies included in the systematic review and meta-analysis of famine exposure in early life and anthropometric profile in adults*

A total of 984 articles were retrieved on the basis of the search strategy. Approximately 624 potential articles were duplicated and removed, with the remaining 360 retrieved for further investigation. After the review of the titles and abstracts, 290 articles were excluded. The full text of the remaining 70 studies was retrieved for detailed evaluation, of which 50 were excluded. Of these 13 studies were excluded due to suboptimal quality (Supplementary file 3). The remaining 20 studies [23–26, 28–34, 45–56] have been included in the current systematic review. Of these, 12 studies were included in the meta-analysis to estimate the relationship between early-life exposure to famine and BMI [23–25, 29, 31, 33, 53], waist circumference [24, 29, 45, 49, 52, 53] and height [24, 26, 27, 31, 33, 53] in adulthood. The detailed characteristics of the studies have been shown in Table 1. The Flow chart diagrams to describe the selection of studies for a systematic review and meta-analysis is shown in Fig. 1.

**Table 1:** Characteristics of studies reporting the association of early life exposure to famine and anthropometric profile in adults, 2021

First Author, Year/country	Famine year /duration	Study Design	Sample size	Age at measure (years)	Outcome studied	Main findings
de Rooij et al., 2007 / Dutch	1944–45 / 6 months	Historical cohort	783	Exposed ~ 58.5 Unexposed ~ 57.4	WC (cm)	Waist circumference (cm): unexposed = 94.1 + 12.4, late gestation exposed = 92.6 + 13.9, mid-gestation = 92.0 + 12.9, early gestation = 89.6 + 11.4
Han and Hon, 2019/ South Korea	1950–53/ 4 year	Historical cohort	25,708	Exposed ~ 59–73 Unexposed ~ 50–55	WC (cm) BMI (kg/m2)	Waist circumference (cm): **Men:** Fetal exposed = 85.87 (0.42), early childhood exposed = 85.46 (0.43), late childhood exposed = 84.26 (0.64), adolescence exposed = 83.11 (0.92), unexposed = 85.86 (0.47), **Women:** Fetal exposed = 82.58 (0.43)**, e**arly childhood exposed = 82.60 (0.44)**,** late childhood exposed = 81.94 (0.64)**,** adolescent exposed = 81.49 (0.92), unexposed = 81.26 (0.47) BMI: Fetal exposed = 24.19 (0.16), early childhood exposed = 24.09 (0.16), late childhood exposed = 23.70 (0.23), adolescent exposed = 23.36 (0.32), Unexposed = 24.24 (0.17), **Women:** Fetal exposed = 24.29 (0.15)**,** early childhood exposed = 24.28 (0.15), late childhood exposed = 24.11 (0.22), adolescent exposed = 23.96 (0.31), unexposed = 23.92 (0.17)
Ning et al., 2019/ China	1959–61 /3 year	Historical cohort	9, 588	Exposed ~ 47–65 Unexposed ~ 40	WC (cm) BMI (kg/m2)	Waist circumference (cm), **men**: Unexposed = 85.0 (0.56), fetal-exposed = 87.2 (0.71), childhood-exposed = 86.5 (0.33), adolescence-exposed = 88.3 (0.69), **women**: Unexposed = 82.1 (0.39), fetal-exposed = 82.8 (0.52), childhood-exposed = 83.2 (0.25), adolescence-exposed = 83.6 (0.55) BMI**, all subjects:** Unexposed = 24.6 (0.12)**,** fetal-exposed = 25.3 (0.15), childhood-exposed = 25.7 (0.07), adolescence-exposed = 26.3 (0.15)
Wang et al., 2017/ China	1959–61/3 year	Historical cohort	6445	Exposed ~ 52–59 Unexposed 40–51	WC (cm)	Waist circumference, **men:** Unexposed = 82.9 ± 8.8, fetal-exposed = 83.9 ± 9.0, childhood-exposed = 83.6 ± 9.5, adolescence = 83.5 ± 9.7, **women**: Unexposed = 74.1 ± 8.2, fetal-exposed = 77.4 ± 8.7, childhood-exposed = 79.6 ± 9.3, adolescence = 81.6 ± 10.2
Wang et al., 2019/ China	1959–61/3 year	Historical cohort	2148	Exposed = 51–55 Unexposed = 48	Height WC (cm) BMI (kg/m2)	Waist circumference (cm): Unexposed = 85.43 (9.80), fetal exposed = 85.70 (10.62), infant = 84.96 (9.75), preschool = 85.50 (10.04)
Stanner et al., 1997/ Leningrad	1941–44/ 6 months	Cross-sectional	549	Exposed = 52–53 Unexposed = 52.8	BMI (kg/m2) Height (m) WHR	BMI (kg/m2): Unexposed = 25.2 (24.1 to 26.3), intrauterine exposed = 24.6 (23.6 to 25.6), infant group = 25.4 (24.2 to 26.6), Height (m): Unexposed = 1.73 (1.71 to 1.75), intrauterine exposed = 1.72 (1.70 to 1.74), infant group = 1.74 (1.72 to 1.76) WHR: Unexposed = 0.87 (0.85 to 0.89), intrauterine exposed = 0.86 (0.84 to 0.88), infant group = 0.88 (0.84 to 0.92)
Shi, Nicholls et al. 2018/ China	1959–61/3 year	Historical cohort	5772	Exposed = 50–57 Unexposed = 47	BMI	BMI: Unexposed cohort = 24.2 (3.6), fetal exposed cohort = 24.3 (4.4), early child exposed = 23.9 (3.9), mid childhood exposed = 23.3 (3.6), late childhood exposed = 23.7 (3.8)
Chen et al., 2019/ China	1959–61/3 year	Historical cohort	5295	Exposed = 52–93 Unexposed = 40–51	WC (cm) BMI	Waist circumference, **men**: Unexposed = 82.9 ± 8.8, fetal exposed = 83.9 ± 9.0, childhood exposed = 83.6 ± 9.5, adolescent exposed = 83.4 ± 9.7, **women**: Unexposed = 74.1 ± 8.2, fetal exposed: 77.4 ± 8.7, childhood exposed = 79.5 ± 9.3, adolescent exposed = 81.6 ± 10.1 BMI, **men** Unexposed = 24.8 ± 3.2**, f**etal exposed = 24.9 ± 3.1**, c**hildhood exposed = 24.7 ± 3.3**, a**dolescent exposed = 23.9 ± 3.4, **women**: Unexposed = 23.6 ± 3.3, fetal exposed = 24.5 ± 3.4, childhood exposed = 24.7 ± 3.6, adolescent exposed = 24.4 ± 3.8**,**
Hult et al., 2010/ Nigeria	1968–70/2 year	Historical cohort	1338	Exposed = 40–43 Unexposed = 37	WC (cm) BMI (kg/m2) Height	Height, cm (mean (SD): unexposed = 170 [8], fetal-infant = 169 [8], early childhood = 169 [8] Waist circumference, cm (mean (SD): Unexposed = 91 [11], fetal-infant = 94 [13], early childhood = 93 [11] BMI, kg/m2: unexposed = 26.5 (4.4), fetal-infant = 27.5 (4.6), early childhood = 26.7 (4.7)
Painter et al., 2006b/ Dutch	1944–45 /6 months	Historical cohort	721		BMI	BMI, kg/m^2^: Born before famine = 28.4, late gestation exposure = 28.1, mid gestation exposure = 27.9, early gestation exposure = 27.9, conceived after famine = 28.8
Liu et al., 2019/China	1959–61/3 year	Historical cohort	18,984	Exposed ~ 41.6–44.6	Height	Height, cm, mean (SD): Unexposed = 161.2 (8.2), fetal-Exposed = 161.0 (8.1), infant-Exposed = 160.4 (8.2)
				Unexposed ~ 38.6		
Meng et al., 2016/China	1959–61/3 year	Historical cohort	94,052	NM	BMI	Exposed: BMI (β-coefficients (95% CI): 0.12, 0.03–0.22)
Portrait et al., 2017/Dutch	1944–45/ 6 months	Historical cohort	1008	Age between 44 and 60 years	Adult Height	Height = Mean (SD), **Exposed** during gestation to age 1 = 170.8 (8.1), early childhood = 171.8 (9.0), late childhood = 171.0 (8.3), puberty = 170.6 (9.0), **Male**: gestation to age 1 = 177.1 (4.9), early childhood = 177.9 (6.8), late childhood = 176.6 (6.0), puberty = 176.6 (6.8) **Female**: gestation to age 1 = 163.4 (3.4), early childhood = 165.5 (6.2), late childhood = 165.2 (6.1), puberty = 163.9 (5.8) Height = Mean (SD), **Unexposed** during gestation to age 1 = 173.4 (8.4), early childhood = 171.7 (9.3), late childhood = 170.9 (9.0), puberty = 169.3 (8.1), **Male**, gestation to age 1 = 179.7 (6.5), early childhood = 178.6 (6.8), late childhood = 178.5 (7.0), puberty = 175.8 (5.8), **Female,** gestation to age 1 = 168.2 (5.8)**, e**arly childhood = 165.7 (6.6)**,** late childhood = 165.3 (5.4)**,** puberty = 164.5 (5.9)
Ravelli et al., 1999/Dutch	1944–45/ 6 months	Historical cohort	6445	Exposed ~ 52–59 Unexposed ~ 40–51	BMI (kg/m2) WC (cm) Height (cm)	BMI (kg/m2): Born before famine = 26.7, late gestation = 26.7, mid gestation = 26.6, early gestation = 28.1 conceived after the famine = 27.2 Waist circumference (cm): Born before famine = 91.8, late gestation = 92.4, mid gestation = 91.0, early gestation = 95.6, conceived after the famine = 92.5 Height (cm): Born before famine = 171.0, late gestation = 170.9, mid gestation = 168.6, early gestation = 171.0, conceived after the famine = 170.9
Song et al., 2020/China	1959–61/3 year	Historical cohort	8054	Exposed = 50.9(50.2–51) Unexposed = 48.4(47.8–49)	WC (cm) BMI (kg/m2)	BMI (kg/m2): Unexposed = 24.1(22.0,26.5), fetal exposed = 24.2(22.0,26.5), WC (cm): unexposed = 82.0(75.4,89.0), fetal exposed = 82.5(76.0,89.3)
Stein et al., 2007 /China	1944–45/ 6 months	Historical cohort	11,784	Exposed age = 58.9 ± 0.49 Control age = 58.8 ± 1.57	Height (cm) WC (cm) BMI (kg/m2)	Height (cm), **Men:** Unexposed = 178.3 ± 6.3, exposed = 177.4 ± 6.2, **Women:** Unexposed = 165.4 ± 6.3, exposed = 165.4 ± 6.6, BMI (kg/m2), **Men:** Unexposed = 27.9 ± 4.0, exposed = 27.8 ± 3.6, **Women:** Unexposed = 26.9 ± 4.5, exposed = 28.8 ± 5.7, WC (cm), **Men:** Unexposed = 101.4 ± 10.5, exposed = 100.5 ± 10.1, **Women:** Unexposed = 93.9 ± 11.1, exposed = 99.0 ± 11.9
van Abeelen et al., 2012c/Dutch	1944–45/ 6 months	Historical cohort	11,784	Ages between 49 and 70 years	BMI (kg/m2) WC (cm)	*BMI* (kg/m^2^) *Mean (SD),* **0–9 years** *=* Unexposed = 25.6 (3.9)*,* Moderately exposed = 25.9 (4.0)*,* Severely exposed = 26.2 (4.3)*,* **10–17 years,** Unexposed = 26.5 (4.0)*,* Moderately = 26.7(4.0)*,* Severely = 26.4 (4.0)*,* **≥18 years =** Unexposed = 26.9(4.3)*,* Moderately = 27.1(4.5)*,* Severely = 27.1 (3.8)*,* *WC (cm)*, *Mean (SD),* **0–9 years** *=* Unexposed = 82.3 (9.6)*,* Moderately = 83.0 (9.8)*,* Severely = 83.8 (10.5)*,* **10–17 years =** Unexposed = 85.7(9.9)*,* Moderately = 86.4(9.9)*,* Severely = 85.8 (10.2) **≥18 years =** Unexposed = 87.0 (9.6)*,* Moderately = 87.2(9.9)*,* Severely = 87.5 (9.9)
Wang, Wang et al. 2010/China	1959–61/3 year	Historical cohort	17,023	Born during 1956–1964	Height (cm)	Height, **Female**: Unexposed = 158.12 cm, gestational = 157.67 cm, toddler = 156.98 cm, **Male**: Unexposed = 168.45 cm, gestational = 168.52 cm, toddler = 167.86 cm
Woo et al., 2010/China	1959–61/3 year	Cross sectional cohort	3732	Men and women aged ≥65 years	BMI (kg/m2) Height (cm)	BMI: Unexposed = 23.46 (3.25), exposed = 23.83 (3.31) Whole body % fat, Unexposed = 29.51 (7.16), exposed = 29.23 (7.19) Height (cm), Unexposed = 157.37 (8.19), exposed = 156.96 (8.24)

*AOR* Adjusted odds ratio, *BMI* Body mass index, *WC* Waist circumference

### Meta-analyses

#### Prenatal exposure to famine and anthropometric profile in adulthood

**Fig. 2:**
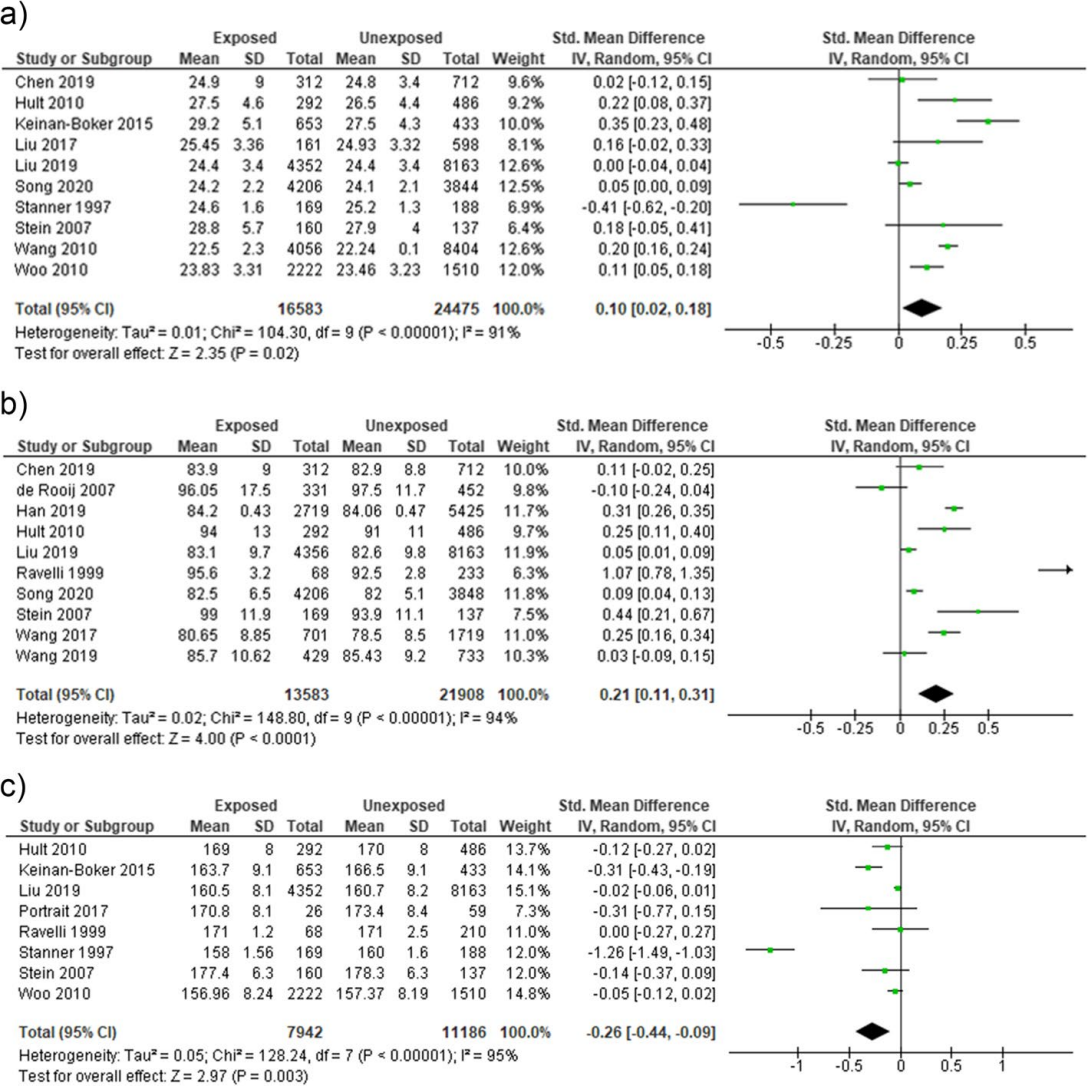
Sensitivity analysis forest plot of prenatal exposure to famine and (**a**) BMI, (**b**) waist circumference (**c**) adult height, 2021

Prenatal famine exposure was associated with body mass index [OR = 0.17 (95% CI: 0.07, 0.27], waist circumference [OR = 0.46 (95% CI: 0.11, 0.82)], and adult height [OR = − 0.30 (95% CI: − 0.53, − 0.08)]. Nonetheless, higher heterogeneity was observed in the analysis of body mass index (*I*^2^ = 95%) and waist circumference (*I*^2^ = 100%), adult height (*I*^2^ = 98%). To further seek heterogeneity sources, sensitivity analysis was performed by omitting one study at a time. The result showed that famine exposure during prenatal life was associated with increased risk of BMI [Standardized Mean Difference (SMD) = 0.10 (95% CI: 0.02, 0.18)], (*I*^2^ = 91%) after omitting the study of Ravelli et al. (1999) [27] **(**Fig. 2a**)**, and waist circumference [SMD = 0.21 (95% CI: 0.11, 0.31)], (*I*^2^ = 94%) after removing the study of Ning et al. (2019) [47] **(**Fig. 2b**)**. Moreover, prenatal famine exposure to famine was associated with decreased adult height [SMD) = − 0.26 (95% CI: − 0.44, − 0.09)], (*I*^2^ = 95%) after omitting the study of Woo (2010) [33] **(**Fig. 2c).

**Fig. 3:**
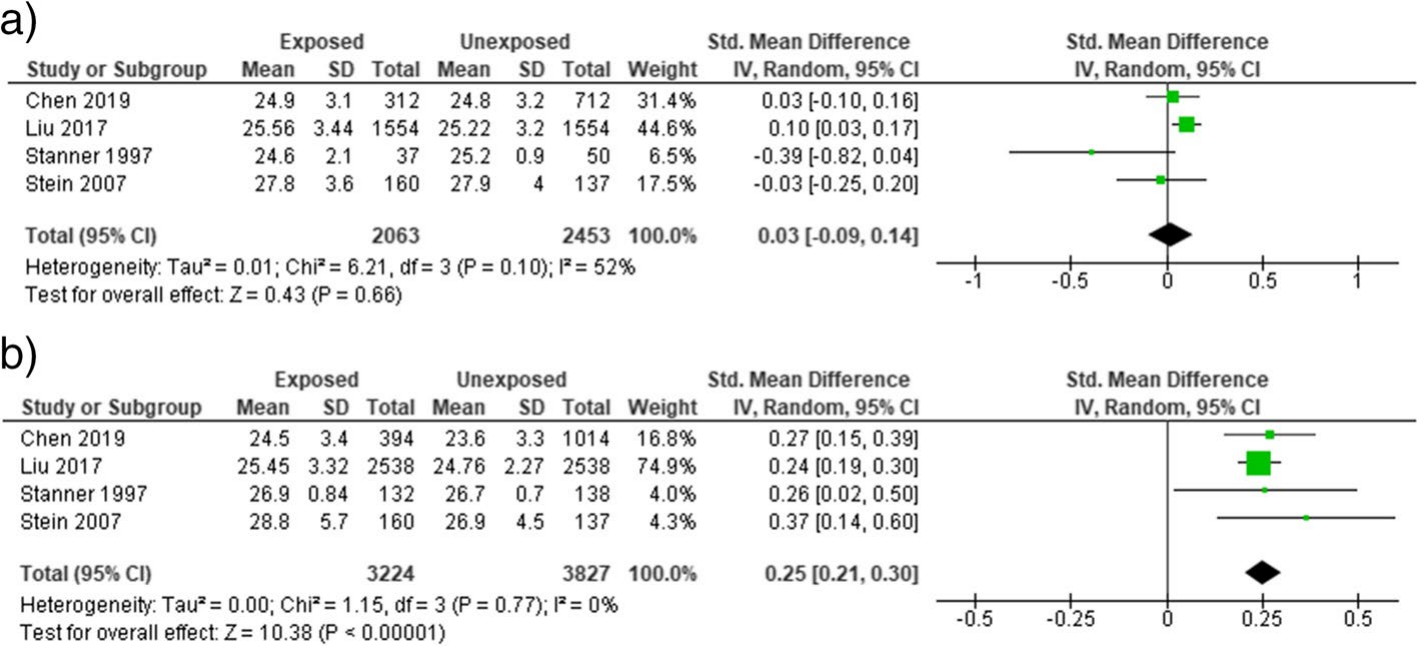
Forest plot of sex-specific effect of prenatal expose exposure to famine on BMI in adults (**a**) male (**b**) female, 2021

**Fig. 4:**
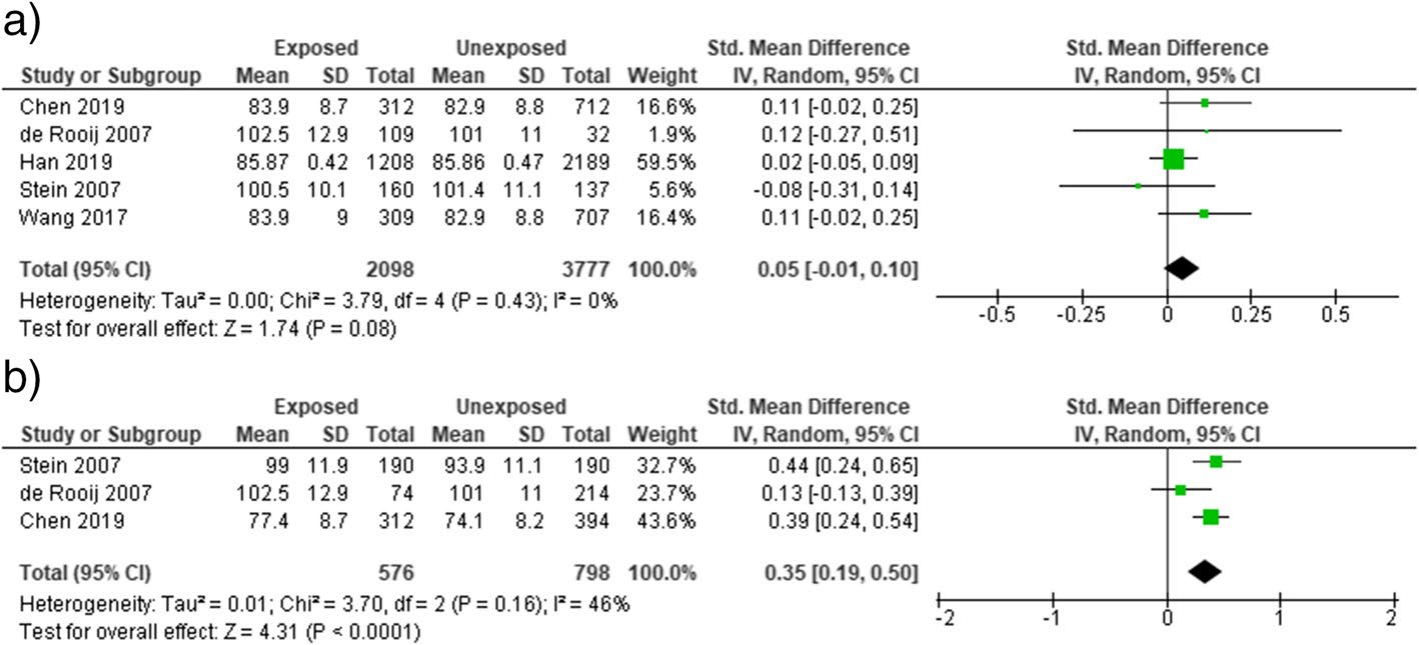
Forest plot of sex-specific effect of prenatal expose to famine on waist circumference in adults (**a**) male (**b**) female, 2021

We also performed subgroup analysis based on the gender of participants. No significant association was observed between prenatal famine exposure and BMI [SMD = 0.03 (95% CI: − 0.09, 0.14), *I*^2^ = 52%] **(**Fig. 3a). In females, famine exposure during prenatal life was significantly associated with the higher BMI [SMD = 0.25 (95% CI: 0.21, 0.30)], (*I*^2^ = 0%) (Fig. 3b). similarly, prenatal famine exposure was not significantly associated with waist circumference in males [SMD = 0.05 (95% CI: − 0.0.01, 0.10)], (*I*^2^ = 0%) (Fig. 4a). However, the association was significant in male participants [SMD = 0.35 (95% CI: 0.19, 0.50)], (*I*^2^ = 46%) (Fig. 4b**)**.

#### Childhood exposure to famine and anthropometric profile in adulthood

**Fig. 5:**
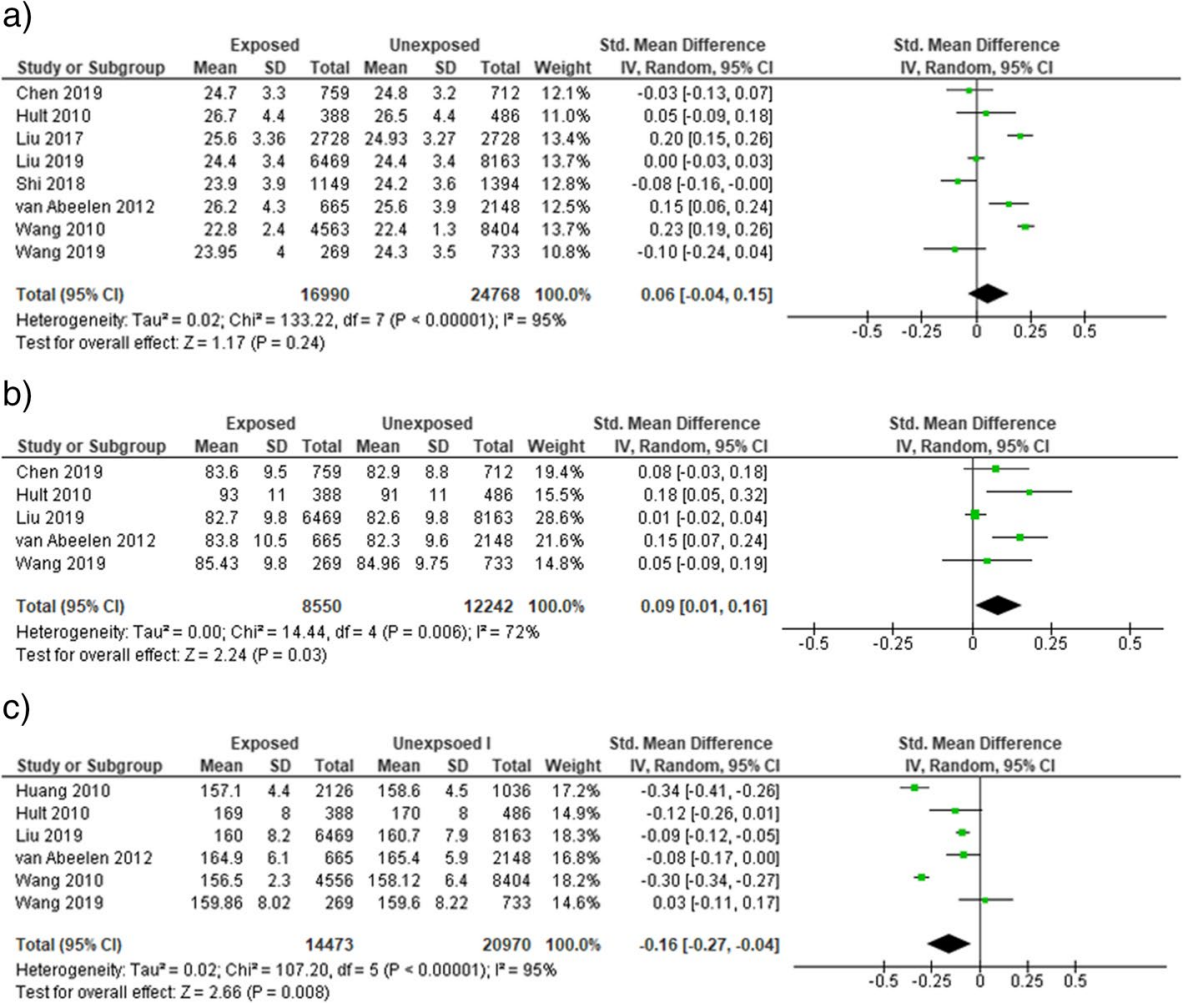
Sensitivity analysis forest plot of childhood exposure to famine and (**a**) BMI, (**b**) waist circumference (**c**) adult height, 2021

Famine exposure during early childhood period of life was not associated with body mass index [OR = 0.06 (95% CI: − 0.04, 0.15)], *I*^2^ = 95% **(**Fig. 5a) in adults. A significant association was observed between early childhood famine exposure and waist circumference [SMD = 0.09 (95% CI: 0.01, 0.16)], *I*^2^ = 72% **(**Fig. 5b) in adults. Similarly, famine exposure during early childhood life was associated with decreased adult height [SMD = − 0.16 (95% CI: − 0.27, − 0.04)], *I*^2^ = 95% (Fig. 5c). We performed sensitivity analysis to identify the sources of heterogeneity. However, no change was observed on the heterogeneity test (*I*^2^).

#### Publication bias

Publication bias was evaluated by funnel plots. As the study sourced out all quality gray literatures, less publication bias was reported for the analysis of metabolic syndrome, diabetes mellitus, and hypertension (**Supplementary file** **4****)**.

## Discussion

We found that famine exposure during prenatal life was also associated with increased BMI and waist circumference and decreased height in adulthood. Early life famine exposure was associated with increased BMI and waist circumference among female participants than males. However, no association was observed between childhood famine exposure and BMI.

Epigenetic change could be one of the mechanisms behind the link between famine experience in childhood and adulthood anthropometric measures. Evidence suggests that famine-induced epigenetic changes such as DNA methylation or programming of the hypothalamic pituitary adrenal (HPA) axis result in catch-up development and long-term impacts on the risk of increased body mass index. According to DOHaD hypothesis, famine exposure during the early stages could change the structure and function of important tissues and organs [1, 2]. Studies also revealed that childhood stunting as a result of nutrient deprivation in early life is associated with decreased height in adults [26, 35, 57].

The review may also reflect a possible sex-difference in the impact of effect of early life famine exposure on adulthood anthropometric measures. In certain parts of the world, particularly in Asian and African countries, parents tend to care sons more than daughters [58, 59]. These preferences may lead to poor health that may increase their susceptibility to increased body mass index, waist circumference and short stature in later life. Moreover, the sex-difference effect might be partly explained by mortality selection where men had higher mortality rates than women during famine [59, 60] Furthermore, females in low-income countries may have experience of physical inactivity, which modifies the effect of early life famine exposure on the increased risk of body mass index and waist circumference [61].

The contemporary relevance of our finding indicate the long term effects of earlier famine and undernutrition are far from over [62, 63]. It contributes to our understanding of the link between childhood malnutrition and a later risk of increased body mass index, waist circumference and short stature. We may be able to design targeted intervention and, eventually, preventative strategies once we have a better knowledge of these processes. As a result, our findings may be valuable in improving health-system awareness of those born during high-risk years, as well as emphasizing the importance of proper nutrition in infancy.

### Strength and limitations

There are various advantages to this systematic review and meta-analysis. As there are potential differences in famine exposure during early life, the study looked into the effects of prenatal and early childhood famine exposure on BMI, waist circumference and adult height. Moreover, the sensitivity and subgroup analysis were performed in order to identify the sources of heterogeneity sources. However, certain potential limitations should be considered in our research. To begin with, the length of the famine varied between research, spanning from 1 to 4 years, which may have influenced the consistency of our findings. Second, the original article did not specify the extent of famine exposure. As a result, we were unable to investigate the relationship between famine severity and BMI, waist circumference and adult height. The other potential limitations of this study include subgroup analyses was not perform according to whether the studies adjusted for age and current lifestyle factors. It would be interesting to see if the results of the studies that performed adjustment for age and current lifestyle factors are different from those that did not perform such an adjustment.

## Conclusion

Results from this study confirmed the relationships between early life, particularly prenatal life, exposure to famine and its association with BMI, waist circumference and adult height. The finding underpinning the nutritional status in early life, has a long-term effect on later life. Further studies on the mechanisms behind the association between early life famine exposure and adulthood anthropometric measures need to be clarified.

## Supplementary Information

**Additional file 1.**

**Additional file 2.**

**Additional file 3.**

**Additional file 4.**

## Data Availability

Data will be available upon reasonable request of the corresponding author.

## Abbreviations

BMI: Body mass index
SMD: Standardized mean difference
REM: Random effects model
DOHaD: Developmental Origins of Health and Disease
PRISMA: Preferred Reporting Items of Systematic Review and Meta-Analysis
WC: Waist circumference
