# Supplementary material for: Early life famine exposure and anthropometric profile in adulthood: a systematic review and Meta-analysis
Source: BMC Nutr. 2022 Apr 22;8:36. doi: 10.1186/s40795-022-00523-w (PMC9028079; doi:10.1186/s40795-022-00523-w)
Supplement: Supplementary file 1 — Additional file 1. [file 40795_2022_523_MOESM1_ESM.docx]

**Supplementary file 1:** Data Extraction form for systematic review and meta-analysis on the long-term effects of famine

| **Authors/year** | **(de Rooij et al., 2007)** | **(Han and Hong, 2019)** | **(Meng et al., 2020)** |
| --- | --- | --- | --- |
| Participant characteristics | **Mean Age**  Exposed =58.5 ± 0.5  Unexposed = 59.2 ± 0.7 (Born before famine) and  57.4 ± 0.6 (Born after famine) | Age at the during famine, y  Fetal exposed = fetal period  Early childhood exposed = 0-4  Late childhood exposed = 5-9  Adolescent exposed = 10-14  Sex = male and female | born between 1 October 1956 and 30 September 1964  aged 39–51 years  both male and female |
| Settings/context | Born as term singletons in the Wilhelmina Gasthuis, a hospital in Amsterdam | both urban and rural setting | urban rural settings |
| Famine year/duration | November 1944 and May 1945 / 7 month | 1950-1953/ 3 year | 1959 - 1961/ 3 year |
| Assessment of famine Exposure | **prenatally exposed** = daily food rations < 1000 calories | Year of birth and age | self- reported birth date at baseline |
| Exposure groups | **In utero** = 7 January 1945 and 8 December 1945  **Late gestation** = 7 January and 28 April 194  **Mid gestation** = 29 April and 18 August 1945  **Early gestation** = 19 August and 8 December 1945  **Unexposed** = born before 7 January 1945 and conceived after 8 December 1945 | Fetal-exposed (1951-1953)  Early childhood exposed (1946-1950)  Late-childhood-exposed (1941-1945)  Adolescent-exposed (1936-1940)  Transition period:  (1954-1958)  Unexposed:(1959-1963) | Pre- famine births (1 October 1956 and 30 September 1958)  Famine births (1 October 1959 and 30 September 1961)  Post- famine births (1 October 1962 and 30 September 1964)  Unexposed = pre- famine + post- famine |
| Types of studies | Retrospective Historical cohort study | Cross-sectional | Prospective cohort study = 10 years |
| sources of information | Medical birth records | Korean national health and nutrition examination survey (KNHANES) | China Kadoorie Biobank (CKB) is a largescale prospective cohort |
| Country of origin of included studies | Netherlands | Korea |  |
| Sample size | Exposed = 331  Unexposed = 452  Total SS = 783 | Exposed = 6758  Unexposed = 4422  Total SS = 25 708 | Exposed = 6758  Unexposed = 4422  Total SS = 92 284 |
| Sampling technique |  | Stratified, multistage, probability sampling | SRS |
| Outcome studied | Metabolic syndrome | Metabolic syndrome | cardiovascular disease  cancer  respiratory disease |
| Outcome measurement/definition | National Cholesterol Education Program (NCEP) | NCEP |  |
| Key findings/Proportions/Mean | **Metabolic syndrome**  Born before the famine = 48%  Late gestation = 51%  Midgestation = 47%  Early gestation = 51%  Conceived after the famine = 47%  **BMI (kg/m2)**  Born before the famine = 28.0 ±1.2  Late gestation = 28.0 ± 1.2  Midgestation = 27.8 ± 1.2  Early gestation = 27.5 ± 1.2  Conceived after the famine = 28.7 ± 1.2  **Waist circumference (cm), Men**  Born before the famine = 101.5 ± 12.7  Late gestation = 100.9 ± 9.3  Midgestation = 99.0 ± 11.2  Early gestation = 102.5 ± 12.9  Conceived after the famine = 101.0 ± 11.0  **Waist circumference (cm), Women**  Born before the famine = 92.6 ±14.2  Late gestation = 92.6 ± 13.9  Midgestation = 92.0 ± 12.9  Early gestation = 89.6 ± 11.4  Conceived after the famine = 94.1 ± 12.4  **Fasting glucose (mmol/L)**  Born before the famine = 5.6 ± 1.1  Late gestation = 5.5±1.1  Midgestation = 5.5±1.1  Early gestation = 5.7 ± 1.1  Conceived after the famine = 5.5 ±1.1  **HDL cholesterol (mmol/L), Men**  Born before the famine = 1.3 ± 1.3  Late gestation = 1.2 ±1.3  Midgestation = 1.3 ± 1.3  Early gestation = 1.2±1.3  Conceived after the famine = 1.3 ± 1.4  **HDL cholesterol (mmol/L), Women**  Born before the famine = 1.7 ± 1.3  Late gestation = 1.6 ± 1.3  Midgestation = 1.6 ± 1.4  Early gestation = 1.7 ± 1.3  Conceived after the famine = 1.7 ± 1.3  **Triacylglycerol (g/L)**  Born before the famine = 1.2 ±1.8  Late gestation = 1.3 ± 1.8  Midgestation = 1.3 ± 1.8  Early gestation = 1.3 ± 1.9  Conceived after the famine = 1.3 ± 1.8  **Systolic blood pressure (mm Hg)**  Born before the famine = 137 ± 1.2  Late gestation = 135 ± 1.2  Midgestation = 136 ± 1.1  Early gestation = 135 ±1.1  Conceived after the famine = 135 ± 1.1  **Diastolic blood pressure (mm Hg)**  Born before the famine = 81 ± 10  Late gestation = 81 ± 10  Midgestation = 80 ±11  Early gestation = 82±10  Conceived after the famine = 82 ± 10 | **Men**  **HDL (mg/dL)**  Fetal exposed = 45.43 (0.56)  Early childhood exposed = 45.25 (0.58)  Late childhood exposed = 44.84 (0.86)  Adolescent exposed = 44.92 (1.20)  Non-exposed = 45.72 (0.61)  **Triglycerides (mg/dL)**  Fetal exposed = 154.41 (6.33)  Early childhood exposed = 143.43 (5.68)  Late childhood exposed = 137.39 (9.23)  Adolescent exposed = 132.35 (13.49)  Non-exposed = 152.73 (6.42)  **Fasting blood glucose (mg/dL)**  Fetal exposed = 111.11 (1.47)  Early childhood exposed = 109.32 (1.50)  Late childhood exposed = 106.85 (2.39)  Adolescent exposed = 102.75 (3.17)  Non-exposed = 109.31 (1.64)  **Systolic blood pressure (mmHg)**  Fetal exposed = 124.41 (0.82)  Early childhood exposed = 124.09 (0.83)  Late childhood exposed = 123.36 (1.27)  Adolescent exposed = 121.72 (1.83)  Non-exposed = 124.73 (0.94)  **Diastolic blood pressure (mmHg)**  Fetal exposed = 78.41 (0.49)  Early childhood exposed = 77.80 (0.49)  Late childhood exposed = 76.56 (0.78)  Adolescent exposed = 75.37 (1.13)  Non-exposed = 78.87 (0.57)  **Waist circumference (cm)**  Fetal exposed = 85.87 (0.42)  Early childhood exposed = 85.46 (0.43)  Late childhood exposed = 84.26 (0.64)  Adolescent exposed = 83.11 (0.92)  Non-exposed = 85.86 (0.47)  **Body Mass Index (kg/m2)**  Fetal exposed = 24.19 (0.16)  Early childhood exposed = 24.09 (0.16)  Late childhood exposed = 23.70 (0.23)  Adolescent exposed = 23.36 (0.32)  Non-exposed = 24.24 (0.17)  **Height (cm)**  Fetal exposed = 166.75 (0.28)  Early childhood exposed = 167.00 (0.28)  Late childhood exposed = 167.14 (0.43)  Adolescent exposed = 166.72 (0.62)  Non-exposed = 167.14 (0.32)  **Women**  **HDL (mg/dL)**  Fetal exposed = 50.19 (0.65)  Early childhood exposed = 45.25 (0.58)  Late childhood exposed = 50.28 (0.62)  Adolescent exposed = 49.36 (1.13)  Non-exposed = 51.37 (0.72)  **Triglycerides (mg/dL)**  Fetal exposed = 141.66 (4.56)  Early childhood exposed = 143.08 (5.19)  Late childhood exposed = 148.75 (6.88)  Adolescent exposed = 145.69 (9.08)  Non-exposed = 137.49 (4.92)  **Fasting blood glucose (mg/dL)**  Fetal exposed = 103.83 (1.36)  Early childhood exposed = 102.17 (1.24)  Late childhood exposed = 99.09 (1.69)  Adolescent exposed = 97.10 (2.21)  Non-exposed = 103.27 (1.21)  **Systolic blood pressure (mmHg)**  Fetal exposed = 122.18 (0.87)  Early childhood exposed = 123.07 (0.85)  Late childhood exposed = 122.17 (1.17)  Adolescent exposed = 120.41 (1.65)  Non-exposed = 122.04 (0.88)  **Diastolic blood pressure (mmHg)**  Fetal exposed = 75.77 (0.48)  Early childhood exposed = 75.61 (0.47)  Late childhood exposed = 74.33 (0.68)  Adolescent exposed = 72.72 (0.98)  Non-exposed = 75.99 (0.51)  **Waist circumference (cm)**  Fetal exposed = 82.58 (0.43)  Early childhood exposed = 82.60 (0.44)  Late childhood exposed = 81.94 (0.64)  Adolescent exposed = 81.49 (0.92)  Non-exposed = 81.26 (0.47)  **Body Mass Index (kg/m2)**  Fetal exposed = 24.29 (0.15)  Early childhood exposed = 24.28 (0.15)  Late childhood exposed = 24.11 (0.22)  Adolescent exposed = 23.96 (0.31)  Non-exposed = 24.24 (0.17)  **Height (cm)**  Fetal exposed = 154.59 (0.25)  Early childhood exposed = 154.86 (0.27)  Late childhood exposed = 154.33 (0.37)  Adolescent exposed = 153.02 (0.52)  Non-exposed = 167.14 (0.32) | **Cerebrovascular disease**  **Men**  non- famine births = 7.8%  Famine births = 8.4%  **Women**  non- famine births = 7.8%  Famine births = 9.4%  **Cancer**  **Men**  non- famine births = 3.2%  Famine births = 3.2%  **Women**  non- famine births = 3.4%  Famine births = 3.5%  **chronic obstructive pulmonary disease**  **Men**  non- famine births = 1.1%  Famine births = 1.0%  **Women**  non- famine births = 0.9%  Famine births = 0.8%  **Cerebrovascular disease**  **Rural areas**  non- famine births = 8.0%  Famine births = 8.4%  **Urban areas**  non- famine births = 7.4%  Famine births = 9.7%  **Cancer**  **Rural areas**  non- famine births = 3.0%  Famine births = 3.1%  **Urban areas**  non- famine births = 3.8%  Famine births = 3.7%  **chronic obstructive pulmonary disease**  **Rural areas**  non- famine births = 1.5%  Famine births = 1.4%  **Urban areas**  non- famine births = 0.3%  Famine births = 0.4% |
| Effect Measures **(**95%CI OF AOR OR COR OR β-coefficients) | **OR, 95% CI,** metabolic syndrome  **Exposed =**1.2 (0.9, 1.7)  **triacylglycerol concentrations**  **Exposed =** 0.1 g/L (0.0, 0.2 g/L) | Fetal exposed men: AOR, 1.28; 95% CI, 0.93-1.76  Fetal-exposed women: OR, 1.35; 95% CI, 1.01-1.80  Early-childhood exposed men: AOR, 1.25; 95% CI, 0.82-1.90  Early-childhood exposed women OR, 1.41; 95% CI, 0.97-2.06).  Late childhood exposed men: 0.95 (0.54-1.68)  Late childhood exposed women: 1.17 (0.70-1.96)  Adolescent exposed men: OR 0.69 (0.34-1.41)  Adolescent exposed women: 0.90 (0.47-1.70)  Transition period: 1.15 (0.96-1.37) | **HRs (95% CIs)**  **Cerebrovascular disease**  **Men**  Famine births = 1.00 (0.91 to 1.09)  **Women**  Famine births = 1.09 (1.01 to 1.17)  **Cancer**  **Men**  Famine births = 0.97 (0.76 to 1.25)  **Women**  Famine births = 1.15 (0.91 to 1.45)  **chronic obstructive pulmonary disease**  **Men**  Famine births = 1.08 (0.83 to 1.41)  **Women**  Famine births = 1.06 (0.84 to 1.33)  **Cerebrovascular disease**  **Rural areas**  Famine births = 1.04 (0.96 to 1.13)  **Urban areas**  Famine births = 1.18 (1.09 to 1.28)  **Cancer**  **Rural areas**  Famine births = 1.07 (0.94 to 1.22)  **Urban areas**  Famine births = 0.97 (0.86 to 1.10)  **chronic obstructive pulmonary disease**  **Rural areas**  Famine births = 0.99 (0.82 to 1.20)  **Urban areas**  Famine births = 1.29 (0.87 to 1.93) |
| Comments | Prenatal exposure to famine or reduced birth weight is not associated with a significantly greater prevalence of the metabolic syndrome. Our findings suggest that, although elements of the metabolic syndrome may be programmed by fetal undernutrition, the origin of the syndrome as a whole is not likely to be found in poor nutrition during gestation. Men exposed to famine in early gestation had significantly lower HDL-cholesterol concentrations did unexposed men | Fetal and early childhood exposure increased the risk for metabolic syndrome in adults compared with the non-exposed group: | prenatal exposure to the chinese famine might be associated with an increased cardiovascular risk and such risk may be modified by adult lifestyle. |
| Adjustment for covariates | Birth weight, BMI, smoking status, participation in sports, and current socioeconomic status, maternal age, maternal weight gain during the third trimester, maternal weight at the end of pregnancy, gestational age, birth weight | Age; household income, smoking status, drinking status, exercise status, and current treatment of hypertension, diabetes (type 1 and type 2), or hyperlipidemia | Sociodemographic characteristics lifestyle behaviors (tobacco smoking, alcohol consumption, physical activity, and intakes of red meat, fresh fruits and vegetables), and personal and family medical history (hypertension, diabetes, and family history of heart attack, stroke and cancer). |

| **Authors/year** | **(Ning et al., 2019)** | **(Peng et al., 2020)** | **(Wang et al., 2017b)** |
| --- | --- | --- | --- |
| Participant characteristics | select from a general population aged 35–74 year  Sex = men and women | Mean age 52.10±4.94  SEX = male and female | Persons >18 years old in the general population |
| Settings/context | urban and rural areas | both urban and rural setting | rural/urban |
| Famine year/duration | 1959 - 1961/ 3 year | 1959 - 1961/ 3 year | 1959 - 1961/ 3 year |
| Assessment of famine Exposure | age at exposure | Year of birth and age | ages when exposed |
| Exposure groups | Fetal-exposed (January 1, 1959 - December 31, 1961),  Childhood exposed (January 1, 1949 - December 31, 1958)  Adolescence/adult-exposed cohorts (January 1, 1931- December 31, 1948)  unexposed (January 1, 1962, and December 31, 1975), | fetal life exposure, between 1959 and 1961  early childhood exposure, between 1956 and 1958  mid-childhood exposure, between 1953 and 1955  late childhood exposure, between 1949 and 1952  Unexposed, born after 1961; | Fetal period (age 52-55 yrs, birth year 1959-1962)  Childhood (age 56-65 yrs, birth year 1949-1958)  Adolescence and young adult (age 66-93 yrs, birth year 1921-1948)  Non-exposed (age 40-51 yrs, birth year 1963-1974 |
| Types of studies | Cross-sectional | Cross-sectional | Cross-sectional |
| sources of information | Survey | 2009 of the China Health and Nutrition Survey | SPECT-China |
| Country of origin of included studies | China | China | China |
| Sample size | Exposed = 5933  Fetal-exposed = 621  Childhood-exposed = 2877  Adolescence exposed = 2435  Unexposed = 3655  Total = 9 588 | Exposed = 2039  Fetal-exposed = 349  Early-childhood =531  Mid-childhood = 536  Late-childhood= 623  Unexposed = 769  Total = 2,808 | Fetal period = 701  Childhood = 1776  Adolescence and young adult = 1053  Non-exposed = 1719  Non-exposed (birth year after 1975) = 1196  Total = 6445 |
| Sampling technique | stratified, random cluster sampling | A multistage, random cluster | stratified cluster sampling |
| Outcome studied | Metabolic syndrome | Metabolic syndrome | Metabolic syndrome |
| Outcome definition | Diabetes Federation (IDF) | Chinese Diabetes Society  (CDS) | IDF |
| Key findings/Proportions/Mean | **Prevalence of Metabolic syndrome**  Unexposed = 21.8%  Overall exposed = 35.3%  Fetal-exposed = 29.5%  Childhood-exposed = 35.4%  Adolescence/adult-exposed = 43.5 %  **Obesity (%)**  Unexposed group = 20.2  Fetal-exposed group = 22.7  Childhood-exposed = 23.3  Adolescence exposed = 24.2  **BMI (kg/m2)**  Unexposed group = 24.6 (0.12)  Fetal-exposed group = 25.3 (0.15)  Childhood-exposed = 25.7 (0.07)  Adolescence exposed = 26.3 (0.15)  **Waist circumference (cm), Men**  Unexposed group = 85.0 (0.56)  Fetal-exposed group = 87.2 (0.71)  Childhood-exposed = 86.5 (0.33)  Adolescence exposed = 88.3 (0.69)  **Waist circumference (cm), Women**  Unexposed group = 82.1 (0.39)  Fetal-exposed group = 82.8 (0.52)  Childhood-exposed = 83.2 (0.25)  Adolescence exposed = 83.6 (0.55)  **SBP (mmHg)**  Unexposed group = 131.3 (0.65)  Fetal-exposed group = 132.6 (0.84)  Childhood-exposed = 133.8 (0.39)  Adolescence exposed = 137.0 (0.86)  **DBP (mmHg)**  Unexposed group = 80.8 (0.38)  Fetal-exposed group = 84.0 (0.50)  Childhood-exposed = 85.8 (0.23)  Adolescence exposed = 86.6 (0.51)  **FPG (mmol/L)**  Unexposed group = -0.06 (0.03)  Fetal-exposed group = 0.02 (0.04)  Childhood-exposed = 0.01 (0.02)  Adolescence exposed = 0.07 (0.04)  **Total cholesterol (mmol/L)**  Unexposed group = -0.07 (0.03)  Fetal-exposed group = 0.01 (0.04)  Childhood-exposed = 0.09 (0.02)  Adolescence exposed = 0.00 (0.04)  **HDL-C (mmol/L)**  Unexposed group = -0.02 (0.03)  Fetal-exposed group = 0.03 (0.04)  Childhood-exposed = 0.06 (0.02)  Adolescence exposed = -0.05 (0.04)  **Triglycerides (mmol/L)**  Unexposed group = 0.08 (0.03)  Fetal-exposed group = 0.07 (0.04)  Childhood-exposed = 0.00 (0.02)  Adolescence exposed = -0.13 (0.04) | **Prevalence of Metabolic syndrome**  No exposure = 24.15%  Fetal childhood = 10.38%  Early-childhood = 14.72%  Mid-childhood = 20.38%  Late-childhood = 30.38% | **Prevalence of Mets**  **Men**  Fetal = 20.1%  Childhood-exposed = 19.1%  Non-exposed = 16.4%,  Women  Fetal = 23.7%  Childhood-exposed = 33.5%  Non-exposed = 13.5%  **Prevalence of components of metabolic syndrome**  **MEN**  **Central obesity, %**  Non-exposed = 23.8  Fetal-exposed = 25.2  Childhood-exposed = 25.6*  Adolescence-exposed = 29.8*  **Raised FPG, %**  Non-exposed = 30.3  Fetal-exposed = 46.3*  Childhood-exposed = 49.0*  Adolescence-exposed = 54.2  **Raised triglycerides, %**  Non-exposed = 48.0  Fetal-exposed = 48.2  Childhood-exposed = 37.2*  Adolescence-exposed = 28.3*  **Reduced HDL, %**  Non-exposed = 13.0  Fetal-exposed = 13.3  Childhood-exposed = 10.7  Adolescence-exposed = 9.6  **Raised BP, %**  Non-exposed = 53.0  Fetal-exposed = 59.5  Childhood-exposed = 67.6  Adolescence-exposed = 81.4  **MEN**  **Waist circumference, cm,**  Non-exposed = 82.9 ± 8.8  Fetal-exposed = 83.9 ± 9.0  Childhood-exposed = 83.6 ± 9.5*  Adolescence-exposed = 83.5 ± 9.7  **FPG, mmol/L**  Non-exposed = 5.6 ± 1.4  Fetal-exposed = 5.9 ± 1.3*  Childhood-exposed = 5.9 ± 1.6  Adolescence-exposed = 5.9 ± 1.3  **HDL, mmol/L**  Non-exposed = 1.34 ± 0.29  Fetal-exposed = 1.36 ± 0.31  Childhood-exposed = 1.41 ± 0.34*  Adolescence-exposed = 1.44 ± 0.35*  **Triglycerides, mmol/L**  Non-exposed = 2.24 ± 2.31  Fetal-exposed = 1.98 ± 1.43*  Childhood-exposed = 1.80 ± 1.83*  Adolescence-exposed = 1.54 ± 1.35*  **Systolic BP, mmHg**  Non-exposed = 128 ± 18  Fetal-exposed = 130 ± 20  Childhood-exposed = 135 ± 19  Adolescence-exposed = 143 ± 21  **Diastolic BP, mmHg**  Non-exposed = 82 ± 13  Fetal-exposed = 82 ± 12  Childhood-exposed = 82 ± 13  Adolescence-exposed = 79 ± 12*  **HOMA-IR**  Non-exposed = 1.38 ± 1.53  Fetal-exposed = 1.41 ± 1.30  Childhood-exposed = 1.49 ± 2.26  Adolescence-exposed = 1.45 ± 2.04  **Women**  **Waist circumference, cm,**  Non-exposed = 74.1 ± 8.2  Fetal-exposed = 77.4 ± 8.7*  Childhood-exposed = 79.6 ± 9.3*  Adolescence-exposed = 81.6 ± 10.2  **FPG, mmol/L**  Non-exposed = 5.4 ± 1.0  Fetal-exposed = 5.7 ± 1.3  Childhood-exposed = 5.8 ± 1.3  Adolescence-exposed = 6.0 ± 1.5  **HDL, mmol/L**  Non-exposed = 1.52 ± 0.31  Fetal-exposed = 1.52 ± 0.31  Childhood-exposed = 1.53 ± 0.33  Adolescence-exposed = 1.55 ± 0.33  **Triglycerides, mmol/L**  Non-exposed = 1.31 ± 1.11  Fetal-exposed = 1.77 ± 2.22*  Childhood-exposed = 1.69 ± 1.17*  Adolescence-exposed = 1.68 ± 1.28  **Systolic BP, mmHg**  Non-exposed = 122 ± 18  Fetal-exposed = 130 ± 18*  Childhood-exposed = 135 ± 21*  Adolescence-exposed = 144 ± 22*  **Diastolic BP, mmHg**  Non-exposed = 75 ± 12  Fetal-exposed = 79 ± 12*  Childhood-exposed = 78 ± 12  Adolescence-exposed = 78 ± 12  **HOMA-IR**  Non-exposed = 1.42 ± 1.28  Fetal-exposed = 1.68 ± 1.87*  Childhood-exposed = 1.73 ± 2.62*  Adolescence-exposed = 1.73 ± 2.39 |
| Effect Measures **(**95%CI OF AOR OR COR OR β-coefficients) | **AOR (95% CI) defined by the IDF**  Fetal-exposed = 1.23 (1.00-1.52)  Childhood-exposed = 1.27 (1.04-1.55)  Adolescence/adult-exposed = 1.23 (0.88-1.72)  **AOR (95% CI) defined by the IDF, stratified by sex**  **Men**  Fetal-exposed = 1.01 (0.71-1.43)  Childhood-exposed = 0.96 (0.69-1.33)  Adolescence/adult-exposed = 0.85 (0.49-1.49)  **Women**  Fetal-exposed = 1.31 (1.01-1.72)  Childhood-exposed = 1.37 (1.06-1.76)  Adolescence/adult-exposed = 1.38 (0.90-2.11) | **AOR (95% CI) defined by the CDS**  Fetal-exposed =0.661 (0.435–1.002)  Early-childhood= 0.706 (0.555–0.898)  Mid-childhood= 1.551 (1.052–2.286)  Late-childhood = 2.455 (1.201–5.019)  **AOR (95% CI)**  **Urban**  Fetal-exposed =0. 825 (0.420–1.622)  Early-childhood= 0.701 (0.473–1.037)  Mid-childhood= 1.477 (0.777–2.810)  Late-childhood = 1.593 (0.479–5.295)  **Rural**  Fetal-exposed = 0.583 (0.341–0.998)  Early-childhood= 0.703 (0.517–0.956)  Mid-childhood= 1.625 (0.992–2.663)  Late-childhood = 3.096 (1.257–7.625)  **AOR (95% CI)**  **Male**  Fetal-exposed = 0.568 (0.318–1.013)  Early-childhood= 0.633 (0.443–0.904)  Mid-childhood= 2.100 (1.218–3.620)  Late-childhood = 3.721 (1.341–10.330)  **Female**  Fetal-exposed = 0.738 (0.397–1.373)  Early-childhood= 0.811 (0.584–1.127)  Mid-childhood= 1.194 (0.678–2.100)  Late-childhood = 1.726 (0.620–4.800) | **AOR (95% CI)**  **Men**  Non-exposed =0.88 (0.51, 1.52)  Fetal-exposed =0.93 (0.62, 1.39)  Childhood-exposed = 0.62 (0.37, 1.02)  Adolescence = 0.51 (0.23, 1.13)  **Women**  Non-exposed = 0.53 (0.32, 0.86)  Fetal-exposed = 1.53 (1.08, 2.17)  Childhood-exposed = 1.95 (1.30, 2.93) *  Adolescence = 1.57 (0.82, 3.02) |
| Comment | Famine exposure in early life is associated with an increased risk of MetS in later life, especially in women. Early-life malnutrition and later life overnutrition were critical in determining adulthood metabolic disorders. | Famine exposure in mid- and late-childhood was associated with the higher risk of MS, especially in rural, males, and severe famine areas | Exposure to famine in early life had sex-specific association with MS |
| Adjustment for covariates | age, study cohorts, residential areas, education levels, income levels, current smoking, and current drinking | age, gender, BMI, smoke, drink, ethnicity, physical activity, region, famine severity, and gross family income | age, smoking, rural/ urban residence, economic status |

| **Authors/year** | **(Wang et al., 2019)** | **(Yu et al., 2018)** | **(Zheng et al., 2012)** |
| --- | --- | --- | --- |
| Participant characteristics | Participants born between 1 January 1956 and 30 September 1964  aged ≥45 yrs old  Sex = men and women | Participants who were born between October 1, 1952 and September 30, 1964  Sex = men and women | Born around the great Chinese famine year (1957–1964), aged from 44 to 51 years |
| Settings/context | urban and rural areas | urban and rural areas | Only urban residents |
| Famine year/duration | 1959 - 1961/ 3 year | 1959 - 1961/ 3 year | 1959 - 1961/ 3 year |
| Assessment of famine Exposure | Birthdates | Birthdates and age | Birthdates and age |
| Exposure groups | Fetal exposed = (10/01/1959–09/30/1961)  Infant exposed = (01/01/1958–12/ 31/1958),  Preschool exposed = (01/01/ 1956–12/31/1957)  Non-exposed (10/01/1962–09/30/1964) | Fetal exposed = October 1, 1959, and September 30, 1961 (current age 52-54 y)  Early childhood = October 1, 1956, and September 30, 1958 (current age 55-57 y)  Mid childhood = October 1, 1954, and September 30, 1956 (current age 57-59 y)  Late childhood = October 1, 1952, and September 30, 1954 (current age 59-61 y) | Fetal exposed = (1960–1961)  postnatal exposed (1957–1958)  Non-exposed = (1963–1964) |
| Types of studies | Cross-sectional | Retrospective cohort | Retrospective |
| sources of information | CHARLS- a large-scale national longitudinal survey |  | 2008 annual physical examinations in Public Health Center: Hospital of Chongqing Medical University |
| Country | China | China | China |
| Sample size | Fetal exposed = 429  Infant exposed = 269  Preschool = 717  Non-exposed = 733  Total = 2148 | Fetal exposed = 1268  Early childhood = 1940  Mid childhood = 1741  Late childhood = 2010  Non-exposed = 956  Total = 7,915 | Fetal exposed = 1022  postnatal exposed = 1344  Non-exposed = 2674  Total = 5040 |
| Sampling technique |  |  | multistep cluster sampling |
| Outcome studied | Metabolic syndrome | Metabolic syndrome | Metabolic syndrome |
| Outcome measurement/definition | CDS | IDF | CDS |
| Key findings/conclusions | **Prevalence of Metabolic syndrome**  Fetal exposed= 37.5%  Infant = 43.5%  Preschool = 37.9%  Non-exposed = 34.0%  **Add the ff findings**  **Overweight/obesity, n (%)**  Fetal exposed = 47.2  Infant = 45.5  Preschool = 43.7  Non-exposed = 50.5  **Central obesity, n (%)**  Fetal exposed = 42.2  Infant = 44.2  Preschool = 41.8  Non-exposed = 43.2  **WC (cm)/mean (SD)**  Fetal exposed = 85.70 (10.62)  Infant = 84.96 (9.75)  Preschool = 85.50 (10.04)  Non-exposed = 85.43 (9.80)  **BMI (kg/m2)/mean (SD)**  Fetal exposed = 24.27 (4.06)  Infant = 23.95 (4.00)  Preschool = 23.84 (3.91)  Non-exposed = 24.30 (3.53)  **Height (cm)/mean (SD)**  Fetal exposed = 159.70 (8.28)  Infant = 159.86 (8.02)  Preschool = 159.34 (8.55)  Non-exposed = 159.60 (8.22)  **SBP (mmHg)/mean (SD)**  Fetal exposed = 126.61 (19.62)  Infant = 128.71 (19.49)  Preschool = 127.00 (20.09)  Non-exposed = 124.46 (18.33)  **DBP (mmHg)/mean (SD)**  Fetal exposed = 77.18 (12.81)  Infant = 77.45 (12.43)  Preschool = 76.32 (12.34)  Non-exposed = 76.12 (12.42)  **AOR (95% CI) defined by CDS**  Fetal exposed = 1.41 (0.93–2.12)  Infant exposed = 1.94 (1.21–3.12)  Preschool exposed = 1.39 (0.97–2.00)  **Men**  Fetal exposed = 1.17 (0.57–2.41)  Infant exposed = 1.98 (0.98–4.02)  Preschool exposed = 1.01 (0.56–1.82)  **Women**  Fetal exposed = 1.69 (1.02–2.80)  Infant exposed = 1.89 (0.97–3.68)  Preschool exposed = 2.08 (1.28–3.38) | Fetal exposed = 25.2%  Early childhood = 26.9%  Mid childhood = 30.3%  Late childhood = 32.7%,  Non-exposed = 32.7  **AOR (95% CI)**  Fetal exposed = 0.96 (0.77-1.20)  Early childhood = 1.24 (1.01-1.52)  Mid childhood = 1.39 (1.13-1.72)  Late childhood = 1.33 (1.08-1.63) | **Prevalence of MetS**  **Men**  Fetal exposed = 22.5%  postnatal exposed = 18.8%  Non-exposed = 20.1%  **Women**  Fetal exposed = 7.3%  postnatal exposed = 8.6%  Non-exposed = 4.0%  **Men**  **Obesity, (%)**  Control = 42.3  Fetally exposed = 42.1  Postnatally exposed = 38.8  **Hypertriglyceridemia, n (%)**  Control = 44.8  Fetally exposed = 43.3  Postnatally exposed = 47.2  **Low HDL-c, n (%)**  Control = 12.8  Fetally exposed = 12.3  Postnatally exposed = 10.3  **Hypertension, n (%)**  Control = 24.3  Fetally exposed = 25.7  Postnatally exposed = 32.1*  **Dysglycemia, n (%)**  Control = 10.7  Fetally exposed = 15.6*  Postnatally exposed = 14.2*  **Women**  **Obesity, (%)**  Control = 17.0  Fetally exposed = 18.5  Postnatally exposed = 25.5*  **Hypertriglyceridemia, n (%)**  Control = 16.8  Fetally exposed = 20.2*  Postnatally exposed = 24.0*  **Low HDL-c, n (%)**  Control = 5.2  Fetally exposed = 5.6  Postnatally exposed = 5.2  **Hypertension, n (%)**  Control = 13.5  Fetally exposed = 16.5*  Postnatally exposed = 19.2*  **Dysglycemia, n (%)**  Control = 3.3  Fetally exposed = 5.6*  Postnatally exposed = 8.1*  **Men**  **BMI (kg/m2)**  Control = 4.4±3.0  Fetally exposed = 24.4±3.1  Postnatally exposed = 24.3±2.9  **SBP (mmHg)**  Control = 125.1±16.6  Fetally exposed =126.7±16.9*  Postnatally exposed = 127.6±18.2*  **DBP (mmHg)**  Control = 81.7±12.2  Fetally exposed = 82.6±12.4  Postnatally exposed = 83.0±12.3  **TC (mmol/l)**  Control = 5.2±0.9  Fetally exposed = 5.2±0.9  Postnatally exposed = 5.2±0.9  **TG (mmol/l)**  Control = 2.4±1.9  Fetally exposed = 2.4±2.1  Postnatally exposed = 2.2±1.7  **HDL-c(mmol/l)**  Control = 1.2±0.3  Fetally exposed = 1.2±0.3  Postnatally exposed = 1.2±0.3  **LDL-c(mmol/l**  Control = 3.1±0.8  Fetally exposed = 3.1±0.8  Postnatally exposed = 3.1±0.8  **FPG(mmol/l)**  Control = 5.3±1.2  Fetally exposed = 5.5±1.6*  Postnatally exposed = 5.5±1.5*  **Women**  **BMI (kg/m2)**  Control = 22.5±2.7  Fetally exposed = 22.8±2.7*  Postnatally exposed = 23.2±2.9*  **SBP (mmHg)**  Control = 117.6±16.2  Fetally exposed = 121.8±16.3*  Postnatally exposed = 123.1±18.3*  **DBP (mmHg)**  Control = 75.3±11.1  Fetally exposed = 76.8±10.6*  Postnatally exposed = 77.0±11.5*  **TC (mmol/l)**  Control = 4.9±0.8  Fetally exposed = 5.0±0.8*  Postnatally exposed = 5.2±0.9*  **TG (mmol/l)**  Control = 1.3±1.0  Fetally exposed = 1.3±0.9  Postnatally exposed = 1.4±1.0  **HDL-c(mmol/l)**  Control = 1.5±0.3  Fetally exposed = 1.5±0.3  Postnatally exposed = 1.5±0.3  **LDL-c(mmol/l**  Control = 2.8±0.7  Fetally exposed = 2.9±0.8*  Postnatally exposed = 3.1±0.8*  **FPG(mmol/l)**  Control = 5.0±0.7  Fetally exposed = 5.1±0.9*  Postnatally exposed = 5.2±1.2*  **AOR (95% CI)**  **Men**  Fetal exposed = 1.15 (0.92–1.44)  postnatal exposed = 1.04 (0.91–1.11)  **Women**  Fetal exposed = 1.87 (1.15–3.04)  postnatal exposed = 1.50 (1.20–1.87) |
| Comment | The metabolic syndrome prevalence in the infant exposed group was significantly higher than that in the unexposed group (d= 8.1%, 95% CI: 2.3, 13.9; rφ = 0.288, P= 0.006) | The association of famine with MetS prevalence risk was stronger among individuals who were overweight/obesity, and exposed to severe famine | We found that exposure to the Chinese famine in early life period was associated with higher risk of metabolic syndrome in adulthood of women, but not men |
| Adjustment for covariates | gender, smoking status, drinking status, physical activity, and the educational levels, BMI | Gender, education, smoking status, drinking status, physical activity, past history of CHD, family history of hypertension and diabetes, fruit intake, vegetable intake, meat intake, BMI and famine severity | age, gender |

| **Authors/year** | **(Chang et al., 2018)** | **(Liu et al., 2019)** | **(Liu et al., 2017a)** |
| --- | --- | --- | --- |
| Participant characteristics | aged 54-56. | Born between October 1, 1956, and September 30, 1964. | born between 1/1/1941 and 12/31/1971 aged 35–74 |
| Settings/context | urban and rural areas | urban and rural | Urban and Rural |
| Famine year/duration | 1959 - 1961/ 3 year | 1959 - 1961/ 3 year | 1959 - 1961/ 3 year |
| Assessment of famine Exposure | Birthdates | Date of birth (DOB) | age at exposure |
| Exposure groups | EXPOSED: born between 1959 and 1961  UNEXPOSED = born between 1955 and 1957 | Fetal-exposed = October 1, 1959, and September 30, 1961  Infant-exposed = October 1, 1956, and September 30, 1958  Unexposed, = October 1, 1962, and September 30, 1964 | Fetal/infant exposed = 01/01/1959 and 12/31/1961)  childhood exposed = 01/01/1949 and 12/31/1958)  Adolescence exposed = 01/01/1941 and 12/31/1948)  Unexposed = 12/31/1971 |
| Types of studies | Retrospective cohort | Retrospective cohort | Cross sectional |
| sources of information | 2015 AND 2011 (CHARLS) national survey | Data were extracted from the China Nutrition and Health Survey (CNHS) | Survey |
| Country | China | China | China |
| Sample size | Exposed = 1092  Unexposed =1616  Total = 2,708 | Fetal Exposed = 4352  Infant Exposed = 6469  Unexposed = 8163  Total = 18,984 | Exposed = 298  Unexposed = 443  Total = 741 |
| Sampling technique |  | stratified, multistage probability cluster sampling design |  |
| Outcome studied | Overweight, Obesity and abdominal Obesity | abdominal obesity | obesity |
| Outcome measurement/definition | body mass index(BMI) and waist circumference | Chinese criteria of weight for adults | Chinese criteria of weight for adults |
| Key findings/conclusions | Prevalence of overweight Exposed = 35.4%  Unexposed = 29.00%  Prevalence of obesity  Exposed = 15.7%  Unexposed = 12.30%  Abdominal obesity  Exposed = 67.9%  Unexposed = 58.70  **AOR (95% CI)**  Exposed group (prenatally and 0-2 years old) =  Overweight = 0.870 (0.661,1.144)  Obesity = 1.085(0.757,1.555)  Abdominal obesity = 0.972 (0.731,1.292)  **Male**  Overweight = 1.169 (0.899, 1.521)  Obesity = 1.249 (0.842, 1.850)  Abdominal obesity = 1.409 (1.095, 1.811)  **Female**  Overweight = 1.357 (1.067, 1.727)  Obesity = 1.356 (1.001, 1.836)  Abdominal obesity = 1.345 (1.038, 1.742) | **Prevalence**  **Overweight**  Fetal-Exposed = 41.3%  Infant-Exposed = 38.4%  Unexposed = 36.3%  **Obesity**  Fetal-Exposed = 9.4%  Infant-Exposed = 9.7%  Unexposed = 8.2%  **Abdominal obesity**  Fetal-Exposed = 19.4%  Infant-Exposed = 18.9 %  Unexposed = 15.5%  **Height, cm, mean (SD)**  Fetal-Exposed = 161.0 (8.1)  Infant-Exposed = 160.4 (8.2)  Unexposed = 161.2 (8.2)  Central Obesity  **AOR (95% CI)**  Fetal-Exposed = 1.31 (1.19–1.44)  Infant-Exposed = 1.28 (1.17–1.40)  **Male**  Fetal = 1.13 (0.98, 1.31)  Infant = 0.99 (0.86, 1.13)  **Female**  Fetal = 1.50 (1.31, 1.72)  Infant = 1.59 (1.41, 1.80)  **Urban**  Fetal = 1.26 (1.08, 1.48)  Infant = 1.13 (0.97, 1.31)  **Rural**  Fetal = 1.36 (1.20, 1.54)  Infant = 1.40 (1.25, 1.56) | **Prevalence**  **Obesity**  Fetal/infant exposed = 25.30%  childhood exposed = 23.27%  Adolescence exposed = 25.49%  Unexposed = 18.77%  **Men**  Fetal/infant exposed = 25.40%  childhood exposed = 18.50%  Adolescence exposed = 18.30%  Unexposed = 21.60%  **Women**  Fetal/infant exposed = 25.20%  childhood exposed = 26.00%  Adolescence exposed = 31.10%  Unexposed = 17.10%  **AOR (95% CI)**  Fetal/infant exposed = 1.59(1.24-2.03)  childhood exposed = 1.42(1.11-1.82)  Adolescence exposed = 1.86(1.25-2.77)  **Male**  Fetal/infant exposed = 1.01(0.72-1.39)  childhood exposed = 1.31(0.94-1.82)  Adolescence exposed = 1.48(0.87-2.50)  **Female**  Fetal/infant exposed = 1.56(1.14-2.14)  childhood exposed = 1.41(1.04-1.92)  Adolescence exposed = 1.76(1.07-2.89)  **Urban**  Fetal/infant exposed = 1.50(1.02-2.19)  childhood exposed = 1.90(1.37-2.62)  Adolescence exposed = 2.94(1.80-4.78)  **Rural**  Fetal/infant exposed = 1.85(1.36-2.53)  childhood exposed = 1.49(1.08-2.05)  Adolescence exposed = 2.03(1.21-3.42) |
| Comment | Undernutrition in early life increased the risks of overweight and obesity in women not in men, and the risk of abdominal obesity was increased with the experience of undernutrition at early age both in men and women. | Exposure to famine during early life was associated with increased risks of abdominal obesity in adulthood, which was partially alleviated by healthy lifestyle factors (e.g., physical activity) | The study showed that famine exposure in early life could increase risks of obesity in adult men and women. |
| Adjustment for covariates | Gender; education, residence, current smoking and current drinking | sex, residential areas, education level, marital status, household income, current drinking status, current smoking status, and physical activity | gender, residence, family monthly income, education, family history diseases, smoking and alcohol habits |

| **Authors/year** | **(Meng et al., 2016)** | **(Portrait et al., 2017)** | **(Ravelli et al., 1999)** |
| --- | --- | --- | --- |
| Participant characteristics | who were born between October 1956 and September 1964 | born between 15 May 1930 and 1 November 1945 | born at term between November 1943 and February 1947 in Amsterdam age 50 y |
| Settings/context | urban and rural areas | urban and rural areas | Wilhelmina Gasthuis, a university hospital in Amsterdam |
| Famine year/duration | 1959 - 1961/ 3 year | 1944–1945 | 1944–1945 |
| Assessment of famine Exposure | Birthdates | place of residence during the Dutch famine | **prenatally exposed** = daily food rations < 1000 calories  Exposed = exposed to famine in late, mid, or early gestation  Unexposed = before or conceived after the famine |
| Exposure groups | Before famine (born between October 1956 and September 1958)  Born during famine (between October 1959 and September 1961)  Born after famine (control group) (between October 1962 and September 1964) | (1) gestation to one year of age = born between May 15th, 1944 and November 1st, 1945),  (2) early childhood = 1–5years (born between May 15th, 1939 and May 14th,1944)  (3) late childhood = 6–10years (born between May 15th, 1934 and May 14th, 1939)  (4) puberty = 11–15years (born between May 15th, 1930 and May 14th, 1934 = 274). | **In utero** = 7 January 1945 and 8 December 1945  **Late gestation** = 7 January and 28 April 194  **Mid gestation** = 29 April and 18 August 1945  **Early gestation** = 19 August and 8 December 1945  **Unexposed** = born before 7 January 1945 and conceived after 8 December 1945 |
| Types of studies | Retrospective cohort | Retrospective cohort | Retrospective |
| sources of information | baseline survey of China Kadoorie Biobank | Longitudinal Aging Study Amsterdam (LASA) | Medical birth records |
| Country | China | Dutch | Dutch |
| Sample size | 94 052 participants | Exposed during gestation to age 1= 26  Early childhood = 116  Late childhood = 112  Puberty = 98  Unexposed = 656  Total = | Born before famine = 210  Late gestation = 120  Mid gestation = 110  Early gestation = 68  conceived after the famine = 233  Total = 440 |
| Sampling technique |  |  | multistep cluster sampling |
| Outcome studied | BMI | Adult height | Obesity |
| Outcome measurement/definition |  | Stadiometer | BMI |
| Key findings/conclusions | In females, the group born during famine had higher BMI (β-coefficients (95% CI): 0.12, 0.03-0.22) | **Height = Mean (SD) exposed**  Gestation to age 1= 170.8 (8.1)  Early childhood = 171.8 (9.0)  Late childhood = 171.0 (8.3)  Puberty = 170.6 (9.0)  **Male**  Gestation to age 1= 177.1 (4.9)  Early childhood = 177.9 (6.8)  Late childhood = 176.6 (6.0)  Puberty = 176.6 (6.8)  **Female**  Gestation to age 1= 163.4 (3.4)  Early childhood = 165.5 (6.2)  Late childhood = 165.2 (6.1)  Puberty = 163.9 (5.8)  **Height = Mean (SD) Unexposed**  Gestation to age 1= 173.4 (8.4)  Early childhood = 171.7 (9.3)  Late childhood = 170.9 (9.0)  Puberty = 169.3 (8.1)  **Male**  Gestation to age 1= 179.7 (6.5)  Early childhood = 178.6 (6.8)  Late childhood = 178.5 (7.0)  Puberty = 175.8 (5.8)  **Female**  Gestation to age 1= 168.2 (5.8)  Early childhood = 165.7 (6.6)  Late childhood = 165.3 (5.4)  Puberty = 164.5 (5.9)  **Coefficient [95% CI]**  Exposed during gestation to age 1= -3.70 [-6.04 – -1.36]  Early childhood = - 0.98 [-2.53–0.55]  Late childhood = -1.34 [-2.65 – -0.02]  Puberty = - 0.37 [-1.81–1.23]  **Males**  Exposed during gestation to age 1= -3.16 [-6.82–0.49]  Early childhood = -1.71 [-4.08–0.65]  Late childhood = -1.70 [- 3.79–0.38]  Puberty = -0.14 [-2.44–2.16]  **Females**  Exposed during gestation to age 1= -4.45 [-7.44 – -1.47]  Early childhood = - 0.31 [-2.36–1.74]  Late childhood = -0.85 [-2.56–0.84]  Puberty = - 0.06 [-2.09–1.95] | **PREVALNCE**  BMI ≥ 25 kg/m2 = OBESITY  Born before famine = 65%  Late gestation = 63%  Mid gestation = 64%  Early gestation = 75%  conceived after the famine = 67%  **BMI (kg/m2)**  Born before famine = 26.7  Late gestation = 26.7  Mid gestation = 26.6  Early gestation = 28.1  conceived after the famine = 27.2  **Waist circumference (cm)**  Born before famine = 91.8  Late gestation = 92.4  Mid gestation = 91.0  Early gestation = 95.6  conceived after the famine = 92.5  **Height (cm)**  Born before famine = 171.0  Late gestation = 170.9  Mid gestation = 168.6  Early gestation = 171.0  conceived after the famine = 170.9  **95% CI, Obesity, Men**  Late gestation = 0.4 (-3.5, 4.5)  Mid gestation = -1.2 (-5.5, 3.3)  Early gestation = 0.5 (-4.6, 6.0)  **Waist circumference (cm)**  Late gestation = 1.8 (-1.4, 4.9)  Mid gestation = -1.0 (-4.5, 2.5)  Early gestation = 1.8 (-2.4, 6.0)  **95% CI, Height (cm), Men**  Late gestation = 0.5 (-1.4, 2.5)  Mid gestation = -1.5 (-3.7, 0.6)  Early gestation = 0.9 (-1.7, 3.4)  **95% CI, Obesity, Women**  Late gestation = -2.1 (-7.0, 3.1)  Mid gestation = -1.3 (-6.3, 3.9)  Early gestation = 7.4 (0.7, 14.5)  **Waist circumference (cm)**  Late gestation = -0.7 (-4.4, 3.0)  Mid gestation = 0.4 (-3.2, 4.1)  Early gestation = 5.7 (1.1, 10.3)  **95% CI, Height (cm), Men**  Late gestation = 0.1 (-1.6, 1.8)  Mid gestation = -0.6 (-2.3, 1.0)  Early gestation = 0.9 (-1.2, 2.9) |
| Comment | Famine exposure during early life, especially during fetal period, might increase risks of overweight and obesity in females  P values for interaction between famine and smoking, alcohol use, physical activity were estimated by likelihood ratio tests  Except physical activity (interaction: P<0.077), both smoking and alcohol use had modification effects on the associations between famine exposure and BMI (interaction: all P<0.001) | Adult height was significantly shorter for females exposed at ages younger than 1 or at ages younger than 2. The results for males were only borderline significant for exposure under age 1 and significant for exposure under age 2. | Maternal malnutrition during early gestation was associated with higher BMI and waist circumference in 50-y-old women but not in men |
| Adjustment for covariates | gender, smoking status, drinking status, physical activity, and the educational levels | Gender, socio-economic status (SES), season of birth, the total number of siblings and the degree of urbanization of the municipality of residence during the Dutch famine | Maternal age, parity, weight at end of pregnancy, weight gain in third trimester, interspinous distance, socioeconomic status at birth, and present level of education, smoking, and alcohol intake |

| **Authors/year** | **(Song et al., 2020)** | **(Stein et al., 2007)** | **(van Abeelen et al., 2012c)** |
| --- | --- | --- | --- |
| Participant characteristics | Age  Exposed = 50.9(50.2,51.6)  Unexposed = 48.4(47.8,49.1) | Exposed age = 58.9 ±0.49  Control age = 58.8 ±1.57 | recruited at ages between 49 and 70 years |
| Settings/context | urban and rural areas | born in hospital | Women Only |
| Famine year/duration | 1959 - 1961/ 3 year | 1944–1945 | 1944–1945 |
| Assessment of famine Exposure | Birthdates | Exposed = Ration of < 900 kcal/d during gestation  + age during famine | place of residence Age at famine exposure, The average daily ration, hunger score |
| Exposure groups | Fetal exposed = 1960 and 1961  unexposed = subjects born in 1963 | Exposed to famine during gestation  Time control subjects (born in the same institution and not exposed to famine during gestation)  Sibling control subjects | Exposure age ===According to the seven stages  childhood (0 to 9 years)  adolescence (10 to 17 years)  young adulthood (18 years or older) |
| Types of studies | Cross-sectional | Retrospective cohort | Retrospective |
| sources of information | cross-sectional 2010–2012 CNNHS | Birth record | From Prospect-EPIC exposed to the Dutch famine |
| Country | China | Dutch | Dutch |
| Sample size | Fetal exposed = 4206  unexposed = 3848  Total = 8054 | Exposed to famine during gestation = 350  Time control subjects (born in the same institution and not exposed to famine during gestation) = 296  Sibling control subjects = 310  Total= 656 | 0–9 years = 4425  10–17 years = 3197  ≥18 years = 487  Unexposed = 3,675  Total = 11,784 |
| Sampling technique |  |  |  |
| Outcome studied | Obesity, Overweight | Anthropometric measures | Overweight |
| Outcome measurement/definition |  | Stadiometer | BMI, WC |
| Key findings/Proportions/Mean | **WC (cm)**  Fetal exposed = 82.5(76.0,89.3)  unexposed = 82.0(75.4,89.0)  **BMI (kg/m2)**  Fetal exposed = 24.2(22.0,26.5)  unexposed = 24.1(22.0,26.5)  **Overweight**  Fetal exposed = 36.7%  unexposed = 36.5%  **Obesity**  Fetal exposed = 13.4%  Unexposed = 13.0%  **Central obesity**  Fetal exposed = 31.8%  Unexposed = 30.8% | **Height (cm) = Men**  Exposed = 177.4 ±6.2  Control = 178.3 ± 6.3  **Height (cm) = Women**  Exposed = 165.4 ± 6.6  Control = 165.4 ± 6.3  **BMI (kg/m2) = Men**  Exposed = 27.8 ± 3.6  Control = 27.9 ± 4.0  **BMI (kg/m2) = Women**  Exposed = 28.8 ± 5.7  Control = 26.9 ± 4.5  **WC (cm)= Men**  Exposed = 100.5 ±10.1  Control = 101.4 ±10.5  **WC (cm)= Women**  Exposed = 99.0 ±11.9  Control = 93.9 ± 11.1 | *BMI* = *Mean (SD)*  **0–9 years**  Unexposed=25.6 (3.9)  Moderately exposed = 25.9 (4.0)  Severely exposed = 26.2 (4.3)  **10–17 years**  Unexposed = 26.5 (4.0)  Moderately=26.7(4.0)  Severely = 26.4 (4.0)  **≥18 years**  Unexposed= 26.9(4.3)  Moderately=27.1(4.5)  Severely= 27.1 (3.8)  ***WC*= *Mean (SD)***  **0–9 years**  Unexposed = 82.3 (9.6)  Moderately = 83.0 (9.8)  Severely= 83.8 (10.5)  **10–17 years**  Unexposed= 85.7(9.9)  Moderately=86.4(9.9)  Severely = 85.8 (10.2)  **≥18 years**  Unexposed = 87.0 (9.6)  Moderately=87.2(9.9)  Severely= 87.5 (9.9) |
| Effect Measures **(**95%CI OF AOR OR COR OR β-coefficients) | **β (95%CI)**  WC = 0.52(0.08,0.96)  BMI = 0.08(−0.07,0.24)  **AORs (95%CI)**  Obesity = 1.05(0.92,1.20)  Overweight = 1.02(0.93,1.11)  Central Obesity = 1.05(0.95,1.16)  **Male = β (95%CI)**  WC = −0.03(−0.73,0.67)  BMI = −0.06(−0.30,0.17)  **AORs (95%CI)**  Obesity = 0.90 (0.73,1.11)  Overweight = 0.98(0.85,1.13)  Central Obesity = 0.93(0.79,1.08)  **Female = β (95%CI)**  WC = 0.93(0.37,1.49)  BMI = 0.19(−0.02,0.40)  **AORs (95%CI)**  Obesity = 1.16(0.98,1.38)  Overweight = 1.05(0.93,1.18)  Central Obesity = 1.15(1.01,1.31) | **95% CI =** Height  Weeks 1–10 **= -**0.30 (-1.72, 1.13)  Weeks 11–20 = -0.35(-1.51, 0.82)  Weeks 21–30 = -1.01(-2.13, 0.11)  Week 31 to delivery = 0.51(-0.62, 1.63)  **95% CI = BMI**  Weeks 1–10 **=** 1**.**06 (- 0.23, 2.34)  Weeks 11–20 = - 0.49 (-1.61, 0.63)  Weeks 21–30 = 0.66 (-0.41, 1.72)  Week 31 to delivery = -0.35 (-1.40, 0.69)  **95% CI =** WC  Weeks 1–10 **=** 3.37 (- 0.73, 7.46)  Weeks 11–20 = -1.63 (-5.19, 1.93)  Weeks 21–30 = 2.08 (-1.31, 5.47)  Week 31 to delivery = -1.17 (-4.49, 2.16) | 95% CI  *BMI* = *Mean (SD)*  **0–9 years**  Moderately exposed = 0.37 (0.10 to 0.64)  Severely exposed = 0.44 (0.07 to 0.82)  **10–17 years**  Moderately exposed = 0.31 (−0.02 to 0.64)  Severely exposed = −0.02 (−0.44 to 0.40)  **≥18 years**  Moderately exposed = 0.41 (−0.52 to 1.34)  Severely exposed = 0.75 (−0.42 to 1.93)  *WC* = *Mean (SD)*  **0–9 years**  Moderately exposed = 1.09 (0.40 to 1.77)  Severely exposed = 0.86 (−0.09 to 1.81)  **10–17 years**  Moderately exposed = 1.22 (0.37 to 2.06)  Severely exposed = 0.44 (−0.64 to 1.52)  **≥18 years**  Moderately exposed = 0.64 (−1.64 to 2.91)  Severely exposed = 1.84 (−1.02 to 4.70) |
| Comment | The female subjects had a significantly higher prevalence of obesity and central obesity | Reduced food availability may lead to increased adiposity later in life in female offspring. | positive association between short and transient undernutrition during postnatal development and BMI, waist circumference, and overweight in adulthood. |
| Adjustment for covariates | gender, education level, economic status, physical exercise, sedentary time, smoking, drinking, the intake of livestock and poultry and the intake of cereal and beans | Smoking status, intake of alcohol, intake of energy as estimated from a food-frequency questionnaire, physical activity level | adjusted for age at start of the famine (October 1, 1944), smoking (pack years), alcohol intake (g/day), level of education (3 categories: low, intermediate, and high), and total energy intake (kcal) |

| **Authors/year** | **(Wang et al., 2010)** | **(Yang et al., 2008)** | **(Horenblas et al., 2017)** |
| --- | --- | --- | --- |
| Participant characteristics | born during 1956–1964 | born in the years of 1959, 1960, 1961 and 1964. | age of58 years |
| Settings/context | Female only  Urban and rural | only rural residents | Hospital Born |
| Famine year/duration |  |  |  |
| Assessment of famine Exposure | birth-year | Birth year | medical history |
| Exposure groups | Toddler group = born 1–3 years before the famine (1956–1958)  Gestational group = born during the famine period (1959–1961)  Control group = born after the famine (1962–1964) | Exposed **=** 1959 – 1961  Unexposed = 1964 | Early gestation = (born between 19 August 1945 and 28 December 1945)  mid gestation= (born between 9 April 1945 and 18 August 1945)  late gestation (born between 7 January 1945 and 28 April 1945)  Control = born before 7 January 1945 and after 8 December 1945 = unexposed to famine in utero. |
| Types of studies | Retrospective cohort | Retrospective cohort | Historical |
| sources of information | Annual physical evaluations in the Public Health Center (hospital) | Data from the 2002 CNHS |  |
| Country | China |  | Dutch famine |
| Sample size | Toddler group = 4,563  Gestational group = 4,056  Control group = 8,404  Total = 17,023 | Exposed = 4,363  Unexposed = 2,693  Total = 7056 | Exposed = 454  Unexposed = 723  Total = 1177 |
| Sampling technique |  |  |  |
| Outcome studied | overweight and obesity | Overweight and obesity | stroke |
| Outcome measurement/definition | Chinese criteria for overweight and obesity | Chinese ‘guideline for prevention and control of overweight and obesity in Chinese adults | International Classification of Diseases (ICD) |
| Key findings/Proportions/Mean | Overweight  Female  Toddler = 26.31*  Gestational = 23.37*  Control = 19.49  Male  Toddler = 46.05  Gestational = 48.88  Control = 48.22  **Obesity**  Female  Toddler = 4.43*  Gestational = 2.85  Control = 3.08  Male  Toddler group = 10.51  Gestational = 10.70  Control = 11.21  Height  Female  Toddler = 156.98 cm  Gestational = 157.67 cm  Control = 158.12 cm  Male  Toddler = 167.86 cm  Gestational = 168.52 cm  Control group = 168.45 cm | **Overweight, obesity: Female**  **Born in years of disasters**  Born in 1959 = 23.87%, 7.03  Born in 1960 = 24.65%, 7.22  Born in 1961 = 25.49%, 6.74  **Born in year w/o disaster**  Born in 1964 = 24.34%, 6.80  **Overweight, obesity: male**  **Born in years of disasters**  Born in 1959 = 30.78%  Born in 1960 = 32.41%  Born in 1961 = 32.35%  **Born in year w/o disaster**  Born in 1964 = 26.48% | **Non-fatal stroke (%)**  Born before = 4.3  Exposed late = 3.9  Exposed mid = 4.2  Exposed early = 5.5  Born after = 3.8  **Death due to stroke (%)**  Born before = 0  Exposed late = 0  Exposed mid = 0  Exposed early = 0  Born after = 0.09 |
| Effect Measures **(**95%CI OF AOR OR COR OR β-coefficients) | Overweight  **Odds ratio (95% CI)**  Female  Toddler group = 1.475 (1.288–1.689  Gestational group = 1.260 (1.089–1.457)  Male  Toddler group = 0.917 (0.835–1.006)  Gestational group = 1.027 (0.933–1.130)  **Obesity**  **Odds ratio (95% CI)**  Female  Toddler group = 1.461 (1.083–1.970)  Gestational group = 0.924 (0.646–1.323)  Male  Toddler group = 0.930 (0.801–1.080)  Gestational group = 0.949 (0.814–1.106) | **OR (95% CI), Overweight, obesity: Female**  **Born in years of disasters**  Born in 1959 = 1.289 (1.063, 1.565), 1.465 (1.088, 1.972)  Born in 1960 = 1.372 (1.136, 1.658), 1.396 (1.039, 1.876)  Born in 1961 = 1.352 (1.103, 1.657), 1.243 (0.894, 1.728)  **OR (95% CI), Overweight, obesity**  Born in 1959 = 0.959 (0.767, 1.199), 1.011 (0.699, 1.463)  Born in 1960 = 0.972 (0.780, 1.211), 1.005 (0.699, 1.446)  Born in 1961 = 1.027 (0.817, 1.291), 0.952 (0.645, 1.405) | Adjusted for gender HR (95% CI)  Exposed late = 1.26 (0.55–2.89)  Exposed mid = 1.26 (0.55–2.89)  Exposed early = 1.13 (0.46–2.74)  **Unadjusted HR (95% CI): Men**  Exposed late = 0.66 (0.15–2.90)  Exposed mid = 0.36 (0.05–2.69)  Exposed early = 1.12 (0.37–3.35)  **Unadjusted HR (95% CI): Women**  Exposed late = 2.01 (0.69–5.89)  Exposed mid = 2.31 (0.84–6.37)  Exposed early = 1.14 (0.25–5.21) |
| Comment | Famine seems to be producing shorter but slimmer males | The higher risks of overweight and obesity in women were caused by malnutrition in fetal life. | we could not find any evidence for any major increased risk of stroke after prenatal exposure to famine, but we cannot exclude the potential more subtle differences in risk of stroke after prenatal exposure to famine. |
| Adjustment for covariates | Age, sex | Geographic areas | Sex, obesity, diabetes, smoking, hypertension |

| **Authors/year** | **(De Rooij et al., 2011)** | **(de Rooij et al., 2012)** | **(He et al., 2018)** |
| --- | --- | --- | --- |
| Participant characteristics | Age  Exposed = 58  Unexposed =59.3 and 57.4  men and women | Age Exposed = 63.4  Unexposed =64.6 3 and 62.6  men and women | Born in 1956–1965, at the ages of 41–50 years during the survey time. |
| Settings/context | Urban and rural | born hospital | Urban and rural |
| Famine year/duration |  |  |  |
| Assessment of famine Exposure | daily food rations = less than 1000 calories | daily food rations = less than 1000 calories | AGE |
| Exposure groups | Exposed = born as term singletons around the 1944–1945 Dutch famine  Unexposed = born before and after the famine | Exposed = born as term singletons around the 1944–1945 Dutch famine  Unexposed = born before and after the famine | Pre-famine cohorts (1956–1958)  Famine cohorts (1959–1962)  Post-famine cohorts (1963–1964)  Reference cohort (1965) |
| Types of studies | Cohort study | Cohort study | cross sectional |
| sources of information | Annual physical evaluations in the Public Health Center (hospital) | medical records | Second National Sample Survey on Disability conducted in 31 provinces in 2006 |
| Country | Dutch | Dutch | China |
| Sample size | Exposed = 485  Unexposed = 334  Total = 819 | Prenatally exposed = 224  Unexposed = 341  Total = 565 | N = 239,055 |
| Sampling technique |  |  |  |
| Outcome studied | Self-reported depression/anxiety | Psychiatric disorders/Personality and stress appraisal | Schizophrenia |
| Outcome measurement/definition | Hospital Anxiety and Depression Scale (HADS) | Scores on the Big Five Inventory (BFI) and the Perceived Stress Scale (PSS) | WHO DAS II and tICD-10) |
| Key findings/Proportions/Mean | **HADS-D score**  Born before famine = 3.1  Exposed in late gestation = 3.3  Exposed in mid gestation = 3.3  Exposed in early gestation= 3.8  Conceived after famine = 3.3 | Scores on BFI= **Openness**  Born before = 34.8  Exposed in late gestation = 35.1  Exposed in mid gestation = 34.4  Exposed in early gestation = 33.3  Exposed in early gestation = 33.3  Conceived after = 33.6  Scores on BFI= **Extraversion**  Born before = 28.0  Exposed in late gestation = 28.0  Exposed in mid gestation = 27.5  Exposed in early gestation = 27.9  Conceived after = 28.5  Scores on BFI= **Agreeableness**  Born before = 33.4  Exposed in late gestation = 33.5  Exposed in mid gestation = 33.9  Exposed in early gestation = 34.1  Conceived after = 33.5  Scores on BFI= **Neuroticism**  Born before = 20.2  Exposed in late gestation = 19.7  Exposed in mid gestation = 21.3  Exposed in early gestation = 20.9  Conceived after = 20.6  **Scores on PSS= means (SD)**  Born before = 19.8  in late gestation = 19.4  in mid gestation = 20.6 Early gestation = 21.0  Conceived after = 20.1 | Conceived after = 34.8  Scores on BFI= **Conscientiousness**  Born before = 33.9  Exposed in late gestation = 33.8  Exposed in mid gestation = 33.3 |
| Effect Measures **(**95%CI OF AOR OR COR OR β-coefficients) | 95% CI  Exposed in late gestation = 0. 9 (0. 4, 2.4)  Exposed in mid gestation = 0. 6 (0. 2, 2. 2)  Exposed in early gestation= 0. 6 (0.6, 4. 7) |  | **Schizophrenia: OR (95% CI)**  Pre-famine cohorts (1956–1958) = 1.37 (0.86, 2.18)  Famine cohorts (1959–1962) = 1.82 (1.11, 2.98)  Post-famine cohorts (1963–1964) = 1.41 (0.89, 2.24) |
| Comment | Self-reported mild-to-severe anxiety symptoms were more prevalent among early exposed men than women | exposed to famine during early gestation had lower conscientiousness scores and women exposed during early gestation had higher agreeableness scores | Famine severity was defined as cohort size shrinkage index  Schizophrenia was estimated by difference-in-difference models  The long-term association between prenatal famine exposure and risk of adult schizophrenia observed in this study only occurred in the rural population |
| Adjustment for covariates | Age, sex | Age, sex, age at delivery, wight gain third trimester, weight at last antenatal visit, primiparous, gestational age, birth weight, head circumference, Education, interaction term sex* famine | gender, ethnicity, marital status, education, and family income. |

| **Authors/year** | **(Huang et al., 2013)** | **(Li et al., 2018)** | **(St Clair et al., 2005)** |
| --- | --- | --- | --- |
| Participant characteristics | Age  Prenatal exposed = 51 - 53  Postnatal exposed = 54-56  Unexposed =49-50  men and women | Adults aged ≥45 | 1956-1958 and 1963-1965 |
| Settings/context | Urban and rural | urban/rule location |  |
| Famine year/duration | 1959- 1961 |  |  |
| Assessment of famine Exposure | Birth year | self-reported famine exposure |  |
| Exposure groups | 1963 = unexposed  1956- 1958 = postnatal life  1959- 1961 = exposed in utero and early postnatal period | Fetal cohort = born or conceived in the famine (1959 and 1962)  Infant= aged 0–1 when famine occurred (1958) = 445  Toddler= aged 1–2 when famine occurred (1956–1957)  Preschool= aged 3–5 when the famine occurred (1954–1955)  Mid-childhood = aged 6–11 when the famine occurred (1948–1953)  Young teenage = aged 12–14 when the famine occurred (1945–1947)  Teenage = aged 15–17 when the famine occurred (1942–1944)  Early adulthood = aged 18–30 when the famine occurred (before 1941) | Prenatal Exposure to the Chinese Famine of 1959-1961 |
| Types of studies | Cross sectional | Retrospective |  |
| sources of information | Mental health epidemiology survey conducted between 2001 and 2005 | CHARLS |  |
| Country | China | China | China |
| Sample size | 4972 | Fetal cohort = 996  Infant= 445  Toddler= 1108  Preschool= 1241  Mid-childhood = 3217  Young teenage = 1143  Teenage = 887  Early adulthood = 2486  Total = 11523 | 14778368  Total records of inpatients and patients |
| Sampling technique | Multistage stratified random sampling | four-stage, stratified, cluster probability sampling design |  |
| Outcome studied | mental illness | Depressive symptoms | Schizophrenia |
| Outcome measurement/definition | GHQ-12 | Center for Epidemiological Studies (Depression Scale short form score ≥12) | World Health Organization International Classification of Diseases (ICD-10) |
| Key findings/Proportions/Mean  Effect Measures **(**95%CI OF AOR OR COR OR β-coefficients) | **GHQ-12 (std) =Men/ Women**  956 =0.76 (1.72)/ 1.13 (1.95)  1957=0.68 (1.59)/ 0.81 (1.71)  1958 =0.74 (1.57)/ 0.89 (1.65)  1959=0.86 (1.84)/ 0.93 (1.79)  1960 =0.70 (1.40)/ 1.15 (2.01)  1961 =0.77 (1.79)/ 0.90 (1.78)  1962 =0.70 (1.46)/ 0.80 (1.65)  1963=0.58 (1.30)/ 0.77 (1.67) | **Depression**  Unexposed = 22.7%  Moderate famine exposed = 28.5%  Sever famine exposed= 37.8%  **BMI, kg/m2: mean (s.d.)**  Unexposed = 23.3 (3.9)  Moderate famine exposed = 23.4 (4.0)  Sever famine exposed= 23.2 (4.1)  **DBP, mmHg: mean (s.d.)**  Unexposed = 75.4 (12.1)  Moderate famine exposed = 76.0 (12.1)  Sever famine exposed= 74.8 (12.1)  **Self-reported chronic conditions, %**  **Lung disease**  Unexposed = 10.2%  Moderate famine exposed = 10.1%  Sever famine exposed= 13.4%  **Kidney disease**  Unexposed = 4.9%  Moderate famine exposed = 6.8%  Sever famine exposed= 7.0%  **Digestive disease**  Unexposed = 19.6%  Moderate famine exposed = 22.8%  Sever famine exposed= 28.2%  **Arthritis**  Unexposed = 29.2%  Moderate famine exposed = 34.2%  Sever famine exposed= 43.7% | **OR (95% CI)**  1959 = 0.89 (0.78-1.03)  1961 = 1.93 (1.68-2.23)  1962 = 0.95 (0.87-1.04) |
|  | **95% GHQ scores VS risk of mental illness**  1959-1961 = 0.95 (0.26, 1.65) VS 2.80 (1.23, 6.39)  1956- 1958 = 0.55 (-0.36, 1.47) 1.95 (0.80, 4.76)) | (95% CI)  Depressive symptoms  Total, % (95% CI) = 26.2 (25.1–27.3)  Rural Men= 24.1 (22.7–25.4)  Urban Men= 2.5 (11.0–14.1)  Rural Women= 36.4 (34.8–38.1)  Urban Women = 22.0 (19.5–24.4)  **AOR** 95% CI **among severe famine exposed**  Fetal cohort = 2.37 (1.25–4.48)  Infant= 0.86 (0.18–4.03)  Toddler= 1.03 (0.47–2.26)  Preschool= 1.19 (0.64–2.20)  Mid-childhood = 1.63 (1.10–2.44)  Young teenage = 2.07 (1.12–3.82)  Teenage = 1.30 (0.66–2.58)  Early adulthood = 2.20 (1.42–3.41) |  |
| Comment | The greater biological vulnerability and stronger natural selection in utero of male versus female fetuses during severe famine may result in a stronger selection effect among men than women, obscuring the deleterious impact of famine exposure on the risk of mental illness in men later in life | Famine during infant, toddler, preschool or teenage stages was not associated with depressive symptom  * Severe famine defined as one or more member of the family died from starvation between 1959 and 1961. | The most exposed cohort conceived during the famine showed a 2-fold increased risk of schizophrenia |
| Adjustment for covariates | Age, sex, famine severity | Age, gender, education, childhood location (rural/urban), current location (rural/urban), marital status, self-perceived family income level, individual income sources, employment status, smoking, drinking, body mass index, diastolic blood pressure, lung disease, chronic kidney disease, digestive diseases and arthritis were matched for or controlled for in the models. | Age, gender |

| **Authors/year** | **(Stein et al., 2009)** | **(Wang and Zhang, 2017)** | **(Xu et al., 2009)** |
| --- | --- | --- | --- |
| Participant characteristics | mean age = 59 years  men and women | prenatal exposure to the Chinese Famine of 1959–1961 | Born in 1956–1965, at the ages of 41–50 years during the survey time. |
| Settings/context | Urban and rural |  | Urban and rural |
| Famine year/duration |  |  |  |
| Assessment of famine Exposure | daily food rations = less than 1000 calories |  | AGE |
| Exposure groups | Exposed = born as term singletons around the 1944–1945 Dutch famine  Unexposed = born before and after the famine | Pre-famine cohort (1955–1958  Famine cohort (1959–1961)  Post-famine cohort (1962–1965) | Prenatal exposure to 1959-1961 Chinese Famine |
| Types of studies | Cohort study | Cohort study | cross sectional |
| sources of information |  | China's Second National Sampling Survey  on Disability in 2006 |  |
| Country | Dutch | China | China |
| Sample size | Exposed = 411  Unexposed = 512  Total = 923 | Pre-famine cohort = 120, 287  Famine cohort = 81, 279  Post-famine cohort = 150, 429  Total = 351995 | 171 822 |
| Sampling technique |  | multi-stage, stratified cluster |  |
| Outcome studied | Quality of Life and Depressive Symptoms | Schizophrenia | Schizophrenia |
| Outcome measurement/definition | Center for Epidemiologic Studies Depression scale. | WHO DAS II and ICD-10) | Chinese Classification of Mental Disorders (CCMD) |
| Key findings/Proportions/Mean  Effect Measures **(**95%CI OF AOR OR COR OR β-coefficients) | Mental component score = 52.6 (9.4)  Physical component score =48.0 (9.6)  Depressive symptoms =11.6(7.3) | %schizophrenia cases  Pre-famine cohort = 0.79  Famine cohort = 0.86  Post-famine cohort = 0.81 |  |
|  | **95% CI**  Mental component   - **Before conception =** -2.48 (-4.46 -0.50) - **During gestation =** -0.07 (-1.15—1.29)   Physical component   - **Before conception =** 1.26 (-0.67 (-4.46 -3.19) - **During gestation =** -0.73 (-1.94—0.48)   Depressive symptoms   - **Before conception =** 2.07 (0.60 - 3.54) - **During gestation =** 0.96 (0.09 -1.88) | **95% CI**  post-famine cohort 0.80 (0.76–0.84)  pre-famine cohort 0.91 (0.87–0.95) | RR for schizophrenia was 1.5 (1960) and 2.05 (1961), respectively. However, theeffectwasexclusivelyfromthe ruralareasRR5 1.68 (1960) and RR 5 2.25 (1961). |
| Comment | Self-reported mild-to-severe anxiety symptoms were more prevalent among early exposed men than women | Those cohorts conceived and born during the famine had a higher risk of schizophrenia in mid-adulthood than cohorts conceived and born before or after the famine  schizophrenia risk was higher for urban residents than for rural residents and higher for females than for male | We observe a 2-fold increased risk of schizophrenia among those conceived or in early gestation at the height of famine with risk related to severity of famine conditions. |
| Adjustment for covariates | Age, sex, schooling attainment | Age, gender, residency | Age, sex |

| **Authors/year** | **(Lopuhaä et al., 2000)** | **(Van Abeelen et al., 2013)** | **(Wang et al., 2017d)** |
| --- | --- | --- | --- |
| Participant characteristics | Age (years)  Exposed = 28.9  Unexposed = 27-30  men and women | Age at recruitment (years)  Exposed = 60.4- 60.8  Unexposed = 59.0 | AGE  Non-exposed group = 47~49  Fetal-exposed = 50~51  Infant-exposed = 53  Preschool-exposed= 54~55 |
| Settings/context | Urban and rural | Women ONLY | Urban and rural |
| Famine year/duration |  |  |  |
| Assessment of famine Exposure | daily food rations = less than 1000 calories | daily food rations = less than 1000 calories | AGE |
| Exposure groups | Exposed = born as term singletons around the 1944–1945 Dutch famine  Unexposed = born before and after the famine | childhood (0 to 9 years)  adolescence (10 to 17 years)  young adulthood (18 years or older) | non-exposed group = 1 October 1962 and 30 September 1964  fetal-exposed = 1 October 1 1959 and 30 September 1961  infant-exposed = 1 January 1958 and 31 December 1958  preschool-exposed = 1 January 1956 and 31 December 1957 |
| Types of studies | Retrospective cohort study | Retrospective Cohort study | cross sectional |
| sources of information |  | Prospect-EPIC | CHARLS |
| Country | Dutch | Dutch | China |
| Sample size | Born before famine =264  late gestation =140  mid-gestation =137  Early gestation =87  Conceived after famine =284  Total = 912 | 295 women | Non-exposed group = 1536  Fetal-exposed = 834  Infant-exposed = 518  Preschool-exposed= 1247  Total = 4136 |
| Sampling technique |  |  |  |
| Outcome studied | obstructive airways disease  Wheeze  Productive cough | Hospitalization for COPD and Asthma | chronic lung disease |
| Outcome measurement/definition | medical history, serum concentrations of total IgE and specific IgE against mite, pollen and cat | International Classification of Disease (ICD) | self-reported |
| Key findings/Proportions/Mean | **Wheeze**  Born before famine = 8.7%  late gestation = 9.3%  mid-gestation = 19.0%  Early gestation = 12.6%  Conceived after famine = 12.0%  **Productive cough**  Born before famine = 4.2%  late gestation = 5.8%  mid-gestation = 8.0%  Early gestation = 2.3%  Conceived after famine = 3.2%  **Obstructive airways disease**  Born before famine = 15.5%  late gestation = 15.0%  mid-gestation = 24.8%  Early gestation = 23.0%  Conceived after famine = 17.3% | **COPD = Number of cases**  Unexposed = 68  Moderately exposed = 89  Severely exposed = 47  **Asthma**  Unexposed = 16  Moderately exposed = 20  Severely exposed = 13  **Obstructive airways disease**  Unexposed = 81  Moderately exposed = 102  Severely exposed = 55 | **PREVALCNE**  fetus-exposed = 6.5%  infant-exposed = 7.9%  preschool exposed = 6.8%  non-exposed = 6.1% |
| Effect Measures **(**95%CI OF AOR OR COR OR β-coefficients) | **Wheeze**  late gestation = 0.9 (0.5 to 1.6)  mid-gestation = 1.9 (1.2 to 3.2)  Early gestation = 1.2 (0.6 to 2.4)  **Productive cough**  late gestation = 1.6 (0.7 to 3.7)  mid-gestation = 2.2 (1.1 to 4.8)  Early gestation = 0.6 (0.1 to 2.7)  **Obstructive airways disease**  late gestation = 0.9 (0.5 to 1.5)  mid-gestation = 1.7 (1.1 to 2.6)  Early gestation = 1.5 (0.9 to 2.6) | **Obstructive airways disease**  Moderately exposed = 1.31 (0.97 to 1.77)  Severely exposed = 1.57 (1.10 to 2.23) | AOR **(**95%CI)  Fetal-exposed group = 0.99 (0.53 to 1.84  Infant-exposed group = 1.95 (1.10 to 3.44)  Preschool-exposed group = 0.99 (0.60 to 1.66) |
| Comment | The link between exposure to famine in mid and early gestation and obstructive airways disease in adulthood suggests that fetal lungs can be permanently affected by nutritional challenges during periods of rapid growth. | Acute undernutrition in childhood or young adulthood is associated with an increased risk of later COPD and asthma hospitalization, possibly through increased sensitivity for tobacco smoke.  Associations between famine exposure and hospitalization for COPD were stronger in ever-smokers than in never-smokers | Severe famine exposure during the period of infancy might increase the risk of chronic lung diseases in male adults. |
| Adjustment for covariates | sex, height and age. | Age at start of the famine (October 1, 1944), smoking (never/past/current and pack years), and level of education (low/intermediate/high; socioeconomic status proxy). | Gender, smoking, and drinking, family economic status, and the highest educational attainment of the parents |

| **Authors/year** | **(Zheng et al., 2017)** | **(Chen et al., 2016)** | **(Wang et al., 2016c)** |
| --- | --- | --- | --- |
| Participant characteristics | AGE  Prenatal-exposed = 51.79 ± 1.19  Postnatal-exposed = 54.95 ± 1.18  Control = 49.02 ± 1.16 | Mean age = 49.93 ± 2.97  men and women | non-exposed (40- 51yr)  fetal-exposed (age 52- 55yrs)  childhood-exposed (56-65yrs)  Adolescence exposed (66–93) |
| Settings/context | Women only | Urban and rural | Urban and rural |
| Famine year/duration |  |  |  |
| Assessment of famine Exposure | Age at birth | Age at birth | Age of Exposure to Famine |
| Exposure groups | Prenatal-exposed = (1960-1961)  Postnatal-exposed = (1957-1958)  Control = (1963-1964) | Unexposed = 1963–1965  exposed during pregnancy = 1960 and 1961  exposed at conception and/or infancy and/or early childhood= 1959–1962 | non-exposed (born between 1963-1974)  fetal-exposed (born between 1959-1962)  childhood-exposed (between 1949-1958)  Adolescence exposed (1921–1948) |
| Types of studies | retrospective | Cohort study | cross sectional |
| sources of information |  |  | SPECT-China |
| Country | China | China | China |
| Sample size | Prenatal-exposed = 1873  Postnatal-exposed = 2403  Control = 4476  Total = 6349 | non-exposed (n = 7513)  1959 (n = 648)  1960 (n = 644)  1961 (n = 750)  1962 (n = 1380)  Total = 10935 | non-exposed = 1740  fetal-exposed =712  childhood-exposed = 1778  Adolescence exposed = 1076  Total = 5306 |
| Sampling technique |  |  |  |
| Outcome studied | **NAFLD** | **NAFLD** | **NAFLD** |
| Outcome measurement/definition | Ultrasound  Liver enzymes | Ultrasound  Liver enzymes | Ultrasound  Liver enzymes |
| Key findings/Proportions/Mean | prenatally = 23.0%  post-natally exposed = 22.9%  non-exposed = 17.3% | non-exposed = 34.02%  1959 = 36.42%  1960 = 34.63%  1961 = 40.27%  1962 = 36.09% | Prevalences =  **Men**  non exposed = 55.9%  fetal-exposed = 55.8%,  childhood exposed = 55.4%  **Women**  non-exposed = 33.0%,  fetal-exposed = 46.3%,  childhood exposed =51.7% |
| Effect Measures **(**95%CI OF AOR OR COR OR β-coefficients) | prenatally = 1.33 (1.041.70)  post-natally exposed = 1.26 (1.03-1.55) | 1959 = 1.132 (0.950–1.350)  1960 = 1.001 (0.840–1.193)  1961 = 1.375 (1.172–1.613)  1962 = 1.142 (1.008–1.294) | **Men**  Fetal exposed = 0.86 (0.59, 1.27)  Childhood exposed = 1.00 (0.74, 1.34)  Adolescence exposed = 0.84 (0.60, 1.19)  **Women**  Fetal exposed = 1.05 (0.65,1.69)  Childhood exposed = 1.65 (0.87, 3.12)  Adolescence exposed = 2.53 (0.88, 7.22) |
| Comment | Prenatally exposed women displayed higher risks of NAFLD and mild, moderate and severe steatosis. | Exposure to the Chinese famine during fetal life and infancy was associated with an increased risk of fatty liver disease in adulthood | malnutrition in early life may influence the development of adult NAFLD; thus pregnant women and their infants and children may require the highest priority in obtaining nutritional relief. |
| Adjustment for covariates | Age, BMI, waist, blood pressure, and blood lipids. | Age | for age, rural/urban residence, and economic status of areas |

| **Authors/year** | **(Brand et al., 2016)** | **(Elias et al., 2005)** | **(Koupil et al., 2009)** |
| --- | --- | --- | --- |
| Participant characteristics | Age at recruitment  Unexposed = 59.0 (9.7)  Moderate = 60.4 (10.1)  Severe = 60.8 (8.4) | ages 2 to 33 years during the famine and 41 to 73 years old at interview  Unexposed = 41-73  Moderately exposed = 42-73  Severely exposed = 43-73 | 48.8 ± 5.6 - 52.7  born between 1910 and 1940  (age range, 1–31 years at the peak of starvation in 1941–1942  women and men |
| Settings/context | women ONLY | women ONLY | Urban and rural |
| Famine year/duration |  |  | 1941–1944 |
| Assessment of famine Exposure | self-reported famine exposure | Individual experiences of famine exposure | Age of Exposure to Famine |
| Exposure groups | Prenatal exposed  post-natal exposed | Unexposed = 1963–1965  exposed during pregnancy = 1960 and 1961  exposed at conception and/or infancy and/or early childhood= 1959–1962 |  |
| Types of studies | retrospective | Cohort study | cross sectional |
| sources of information | Prospect-EPIC |  |  |
| Country | Dutch | Dutch | Leningrad |
| Sample size | Unexposed = 3595  Moderate = 3001  Severe = 1310  Total = 7906 | Unexposed = 1,179  Moderately exposed = 917  Severely exposed = 242  Total = 2338 | non-exposed = 1740  fetal-exposed =712  childhood-exposed = 1778  Adolescence exposed = 1076  Total = 5306 |
| Sampling technique |  |  |  |
| Outcome studied | colorectal cancer (CRC) | Cancer risk | death from cancer |
| Outcome measurement/definition | International Classification of Diseases for  Oncology | Population-based project, called Diagnostisch Onderzoek Mammacarcinoom |  |
| Key findings/Proportions/Mean | Unexposed = 2.6%  Moderate = 3.2%  Severe = 3.8% | Unexposed = 60.8%  Moderately exposed = 72.9%  Severely exposed = 87.6% | Women who were 10– 18 years old at the peak of starvation were taller as adults (age adjusted difference, 1.7 cm; 95% CI, 0.5–3.0) and had a higher risk of dying from breast cancer compared with unexposed women born during the same period (age-adjusted HR, 9.9; 95% CI, 1.1–86.5). |
| Effect Measures **(**95%CI OF AOR OR COR OR β-coefficients) | 95% CI = HR  Moderate = 1.15 (0.87–1.53)  Severe = 1.35 (0.96–1.90) | **HR (95% CI)**  Moderately exposed = 1.10 (0.96-1.27)  Severely exposed = 1.25 (1.01-1.55) | HR (95% CI)  Men = 1.14 (0.97–1.33)  Women = 0.90 (0.61–1.33) |
| Comment | severe exposure to a short period of caloric restriction in pre-adult women may relate to colorectal cancer (CRC) risk decades later | We found no indications that this brief famine has affected overall cancer risk, exclusive of breast cancer. | The experience of severe starvation and stress during childhood and adolescence may have long-term effects on cancer in surviving men and women.  Higher risk of dying from breast cancer compared with unexposed women born during the same period |
| Adjustment for covariates | age at start of famine, height, BMI, physical activity, level of education,NSAID use at baseline, OAC use and duration, HRT use and duration, calcium, red meat, processed meat, fibre, familial history of CRC, diagnosis of intestinal polyps or diabetes. | age at examination, BMI, height, socioeconomic status, and cigarette smoking habits. | Cox regression, stratified by gender and period of birth, adjusted for age, smoking, alcohol and social characteristics |

| **Authors/year** | **(Painter et al., 2006a)** | **(Lumey et al., 2012)** | **(Painter et al., 2006c)** |
| --- | --- | --- | --- |
| Participant characteristics | Age at interview  Born before = 59.2  Late = 58.5  Mid = 58.3  Early = 58.1  Conceived after = 57.5 | Age  Exposes = 58.7  Unexposed = 58.6 | persons conceived during the famine were 3 y younger than the unexposed persons at the time of CAD diagnosis (47 y compared with 50 y |
| Settings/context | women |  | Urban and rural |
| Famine year/duration |  |  | 1941–1944 |
| Assessment of famine Exposure | average daily ration | Date of birth | Ration < 1000 kcal |
| Exposure groups | Late gestation (born between January 7–April 28, 1945)  Midgestation (born between April 29–August 18, 1945)  Early gestation (born between August 19–December 8, 1945) | Mother exposed in gestational weeks  1-10 = 26 November 1944 and 4 March 1945  11-20 = 18 September 1944 and 24 December 1944  21-30 = 10 July 1944 and 15 October 1944  31 to delivery = 2 May 1944 and 24 August 1944 | Late gestation (born between 7 January and 28 April 1945)  Midgestation (born between 29 April and 18 August 1945)  Early gestation (born between 19 August and 8 December 1945)  Unexposed = Born between 1 November 1943 and 6 January 1945 (born before the famine) and between 9 December 1945and28 February 1947 (conceived after the famine) |
| Types of studies | retrospective | Cohort study | Retrospective |
| sources of information | Dutch Famine Birth Cohort |  |  |
| Country | Dutch | Dutch | Dutch |
| Sample size | Born before = 144  Late = 82  Mid = 77  Early = 46  Conceived after = 126  Total = 475 | Exposure in late gestation = 131  Exposure in mid gestation = 166  Exposure in early gestation = 113  Born before or after famine = 665  Total = 1,075 | Born before the famine = 289  Late gestation = 160  Midgestation = 138  Early gestation = 87  conceived after the famine = 301  Total =975 |
| Sampling technique |  |  |  |
| Outcome studied | Breast Cancer | coronary artery disease | Early onset of coronary artery disease |
| Outcome measurement/definition | Women were asked whether they had ever  been diagnosed with cancer, and if so, what type of cancer and at what age | Framingham risk |  |
| Key findings/Proportions/Mean | Breast cancer (%)  Born before = 2.8%  Late = 3.7%  Mid = 3.9%  Early = 8.7%  Conceived after = 0.8% | Exposure in late gestation = 8%  Exposure in mid gestation = 6%  Exposure in early gestation = 7%  Born before or after famine = 6% | Born before the famine = 48%  Late gestation = 44%  Midgestation = 39%  Early gestation = 44%  conceived after the famine = 53% |
| Effect Measures **(**95%CI OF AOR OR COR OR β-coefficients) | HR (95% CI)  Late = 2.2 (0.5–9.3)  Mid = 2.3 (0.5–9.6)  Early = 5.8 (1.5–21.7) | HR (95% CI)  Exposure in late gestation = 1.31 (0.67 to 2.57)  Exposure in mid gestation = 0.92 (0.48 to 1.78)  Exposure in early gestation = 1.26 (0.59 to 2.70) | conceived during the famine = (HR:1.9 (1.0, 3.8) |
| Comment | women exposed to prenatal famine more often reported a history of breast cancer than non-exposed women  Prenatal famine may increase breast cancer incidence | no relation between prenatal famine and adult CAD, Framingham risk, or any ECG predictors of increased cardiac disease risk. | We found an earlier onset of CAD among persons conceived during the famine, which suggests that maternal nutrition in early gestation may play a role in the onset of CAD. |
| Adjustment for covariates | age at start of famine, height, BMI, physical activity, | age at examination, age at examination squared, BMI, height, socioeconomic status, and cigarette smoking habits. | Sex, social class or size at birth |

| **Authors/year** | **(Roseboom et al., 2000b)** | **(Huang et al., 2014)** | **(Lv et al., 2020)** |
| --- | --- | --- | --- |
| Participant characteristics | Singletons born alive between November 1943 and February 1947 for whom detailed birth records were available  Men and women | Exposure to famine  during gestation and early postnatal life  (1959–1961) | Age in 2011  Fetal exposed = 49～52  Preschool exposed = 54～57  school-aged exposed = 58～61  non-exposed groups = 44～47 |
| Settings/context | Community study | women | Urban and rural |
| Famine year/duration |  |  |  |
| Assessment of famine Exposure | average daily ration | Date of birth | Based on the birth year |
| Exposure groups | Born before famine  (1/11/43 to 6/1/45)  Late gestation  (7/1/45 to 28/4/45)  Mid-gestation  (29/4/45 to 18/8/45)  Early gestation  (19/8/45 to 8/12/45(  Conceived after famine  (9/12/45 to 28/2/47) | Pre-famine cohort (1957–58)  Famine cohort (1959–61)  Post-famine cohort (1962–63)  Unexposed cohort (1964–65) | non-exposed (born between January 1, 1964 to December 31, 1967),  fetal-exposed (born between January 1, 1959 to December 31, 1962),  preschool exposed (born between January 1, 1954 to December 31, 1957)  school-aged exposed (born January 1, 1950 to December 31, 1953). |
| Types of studies | Historical cohort study | Cohort study | Retrospective |
| sources of information | Dutch Famine Birth Cohort |  | CHARLS 2011–2012 |
| Country | Dutch | China | China |
| Sample size | late gestation (n = 120)  mid-gestation (n = 108)  early gestation (n = 68)  born in the year before the famine or those conceived in the year after the famine (non-exposed subjects = 440)  Total =736 | Rural sample (N = 65 184)  Urban sample (N = 5359)  Total = 70543 | Fetal exposed = 1199  Preschool exposed = 1357  school-aged exposed = 1887  non-exposed groups = 1824  Total = 6267 |
| Sampling technique |  |  |  |
| Outcome studied | Coronary heart disease | proteinuria | CKD |
| Outcome measurement/definition |  |  | glomerular filtration rate (eGFR) = calculated according to Japanese coefficient–modified Chronic Kidney Disease Epidemiology Collaboration equation.  CKD was defined as eGFR less than 60 mL/min per 1.73 m2. |
| Key findings/Proportions/Mean | Born before famine = 3.8%  Late gestation = 2.5%  Mid-gestation = 0.9%  Early gestation = 8.8%  Conceived after famine = 2.6% | **proteinuria in the rural sample, AOR 95% CI**  Pre-famine cohort = 1.28 (0.73, 2.25)  OR (95% CI) Famine cohort = 1.53 (1.04, 2.26)  Post-famine cohort = 1.26 (0.99, 1.59)  **proteinuria in the urban sample**  Pre-famine cohort = 0.63 (0.18, 2.21)  OR (95% CI) Famine cohort = 0.90 (0.36, 2.28)  Post-famine cohort = 1.17 (0.65, 2.10) | Fetal exposed = 4.27%  Preschool exposed = 5.41%  school-aged exposed = 9.65%  non-exposed groups = 2.42%  **Male**  Fetal exposed = 4.64%  Preschool exposed = 5.40%  school-aged exposed = 8.40  Non-exposed group = 1.74%  **Female**  Fetal exposed = 3.89%  Preschool exposed = 5.41%  school-aged exposed = 10.75%  Non-exposed group = 2.84 |
| Effect Measures **(**95%CI OF AOR OR COR OR β-coefficients) | Exposed in early gestation = 3.0 (1.1 to 8.1)  Exposed in mid gestation = 0.9 (0.3, 0.0 to 2. 2)  Exposed in late gestation = 2.5 (0.8, 0.2 to 2.8) | Pre-famine cohort (1957–58) = 0.63 (0.18, 2.21)  Famine cohort (1959–61) = 0.90 (0.36, 2.28)  Post-famine cohort (1962–63) = 1.17 (0.65, 2.10) | Fetal exposed = 1.79 (1.14–2.80)  Preschool exposed = 2.28 (1.50–3.45)  school-aged exposed = 4.25 (2.86–6.32)  **Male**  Fetal exposed = 2.63 (1.21–5.70)  Preschool exposed = 3.06 (1.46–6.41)  school-aged exposed = 4.90 (2.39–10.07)  **Female**  Fetal exposed = 1.41 (0.80–2.47)  Preschool exposed = 1.93 (1.16–3.20)  school-aged exposed = 4.05 (2.52–6.51) |
| Comment | The prevalence of coronary heart disease was significantly greater in people exposed in early gestation than in those who were not exposed prenatally. The prevalence of coronary heart disease was not increased in those exposed in mid-gestation or late gestation. The effect of exposure to famine in early gestation was independent of gestational age (weight of the baby at birth and weight of the mother | Severe undernutrition during gestation and the early postnatal period may have long-term effects on levels of proteinuria in humans, but the effect sizes may be small.  No association was observed among urban samples. Results were robust to adjustment for covariates. | Severe famine exposure as a fetus might increase the risk of chronic kidney disease in male adults. |
| Adjustment for covariates | Age, gender, blood pressure | controlling for height, weight status, hypertension, occupation, education, month of birth and ethnicity. | adjusted for age, area affected by famine, marital status, household per capita income, history of kidney disease, hypertension, diabetes or high blood sugar, smoking, drinking, rural/urban residence and highest educational attainment of parents. |

| **Authors/year** | **(Painter et al., 2005)** | **(Wang et al., 2018a)** | **(de Groot et al., 2011)** |
| --- | --- | --- | --- |
| Participant characteristics | aged 48 to 53, who were born as term singletons in a university hospital in Amsterdam, | Age in 2014  Exposed = 52~ 93  Unexposed = 40 ~ 51 | age of 59 years  men and women who were whose mothers experienced famine during or immediately preceding pregnancy  born in the same three institutions during 1943 and 1947, whose mothers did not experience famine during this pregnancy (same-sex siblings of those in the first two categories |
| Settings/context | Community study | women | born in birth clinics |
| Famine year/duration |  |  |  |
| Assessment of famine Exposure | average daily ration | Date of birth | official ration of <900 kcal/day between November 26, 1944 and May 12, 1945 |
| Exposure groups | Born before  Late  Mid  Early  Conceived after | Fetal-exposed (1959–1962)  Childhood-exposed (1949–1958)  Adolescence/adult-exposed (1921–1948)  Non-exposed (1963–1974) | Prenatal exposed = (infants whose mothers were already 2 months pregnant when the famine started, or who conceived during the famine or in the month following its end) and hence were exposed to a ration of <1000 kcal/day for at least 3 months of pregnancy  Time controls = Infants whose mothers did not experience famine during this pregnancy (births from 1943 and 1947) |
| Types of studies | Historical cohort study | Cohort study | Retrospective |
| sources of information | Dutch Famine Birth Cohort |  |  |
| Country | Dutch | China | Dutch |
| Sample size | ^Born Before = 207^  ^Late =119^  ^Mid = 104^  ^Early = 65^  ^Conceived after = 229^  ^Total = 724^ | Fetal-exposed = 647  Childhood-exposed = 1679  Adolescence/adult-exposed = 1003  Non-exposed = 1795  Total = 5124 | Exposed during gestation = 349  Time controls = 289  Sibling controls = 308  Total = 946 |
| Sampling technique |  |  |  |
| Outcome studied | microalbuminuria (MA) | CKD | cognitive functioning |
| Outcome measurement/definition | albumin/creatinine ratio >2.5 | CKD was defined as eGFR less than 60 mL/min per 1.73 m2. | comprehensive test battery |
| Key findings/Proportions/Mean | MA (ACR >2.5 %)  Born Before famine =8%  Late gestation exposure =7%  Mid gestation exposure =12%  Early gestation exposure = 9%  Conceived after famine = 4%  **creatinine clearance female (ml/min)**  Born Before famine = 110  Late gestation exposure = 115  Mid gestation exposure = 110  Early gestation exposure = 128  Conceived after famine = 119  **Creatinine clearance male (ml/min)**  Born Before famine = 120  Late gestation exposure = 128  Mid gestation exposure = 121  Early gestation exposure = 127  Conceived after famine = 127 | **eGFR, mL/min per 1.73 m2**  Fetal-exposed = 87.8 ± 13.0*  Childhood-exposed = 83.4 ± 12.3  Adolescence/adult-exposed = 74.9 ± 13.4  Non-exposed = 95.0 ± 12.3  **Creatinine (μmol/L)**  fetal-exposed = 68.7 ± 11.8  childhood-exposed = 68.7 ± 12.6  adolescence/young adult-exposed = 71.5 ± 17.5  non-exposed = 66.6 ± 9.7 | General cognitive index  Exposed during gestation = 99.3± 14.7  Time controls = 100.6±14.1  Sibling controls = 100.2 ±16.1 |
| Effect Measures **(**95%CI OF AOR OR COR OR β-coefficients) | **MA, AOR, (95% CI)**  Late gestation exposure = 1.27 (0.49–3.26)  Mid gestation exposure = 3.22 (1.34–7.65)  Early gestation exposure = 1.89 (0.59–6.11) | CKD  fetal-exposed =2.42(1.05, 5.58)  childhood-exposed = 1.23(0.52, 2.90)  adolescence/young adult-exposed = 1.18(0.39, 3.59)  **eGFR**  Fetal-exposed = −1.35(−2.67, −0.04)  Childhood-exposed = 0.29(−1.38, 1.96)  Adolescence/young adult-exposed = 0.89(−1.80, 3.59) | General cognitive index  Whole sample (n¼946) Estimatea (95% CI) = - 0.57 (- 2.41 to 1.28)  Within-sibling pairs = - 1.03 (- 4.97 to 2.91) |
| Comment | Midgestation is a period of rapid increase in nephron number, which is critical in determining nephron endowment at birth. Fetal undernutrition may lead to lower nephron endowment with consequent MA in adult life. The effect of famine was independent of size at birth. | Severe undernutrition during gestation and the early postnatal period may have long-term effects on levels of proteinuria in humans, but the effect sizes may be small.  No association was observed among urban samples. Results were robust to adjustment for covariates. | We found no overall association between maternal exposure to acute famine in pregnancy and cognitive performance of the offspring at the age of 59 years, but cannot rule out an association specific to early pregnancy exposure. |
| Adjustment for covariates | Gender, age, adult BMI, smoking, SES (ISEI-92), systolic BP, IG | controlling for height, weight status, hypertension, occupation, education, month of birth and ethnicity. | Age, , educational level, sex, alcohol intake, smoking, hospital of birth (Amsterdam, Leiden, Rotterdam, sibling) and where necessary test version and clustering of siblings. |

| **Authors/year** | **(Rong et al., 2019)** | **(Rong et al., 2018)** | **(Wang et al., 2016a)** |
| --- | --- | --- | --- |
| Participant characteristics | Unexposed = 52.0 (0.7)  Fetal Exposed = 55.1 (0.7)  Early Childhood = 58.0 (0.7)  Mid Childhood = 60.0 (0.7)  Late Childhood = 62.0 (0.7) | Non-exposed (age = 53-54)  Fetal exposed group (age = 56–57)  Early childhood-exposed group (age = 59–60)  Mid childhood-exposed group (age = 61–62)  Late childhood-exposed group (= 63–64) | Non-exposed group (age = 51–53)  Fetal-exposed group (age = 54–56)  Early childhood-exposed group (age = 57–59)  Mid childhood-exposed group (age = 60–62)  Late childhood-exposed group (age = 63–65) |
| Settings/context | Community study | women and men | women and men |
| Famine year/duration |  |  |  |
| Assessment of famine Exposure | date of birth | Date of birth | Date of birth |
| Exposure groups | Unexposed group (born from 1 October 1962 to 30 September 1964)  Fetal-exposed group (born from 1 October 1959 to 30 September 1961)  Early childhood-exposed group (born from 1 October 1956 to 30 September 1958)  Mid childhood-exposed group (born from 1 October 1954 to 30 September 1956)  Late childhood-exposed group (born from 1 October 1952 to 30 September 1954) | Non-exposed group were born between October 1st, 1962 and September 30th, 1964)  Fetal exposed group (born between October 1st, 1959 and September 30th, 1961  Early childhood-exposed group (born between October 1st, 1956 and September 30th, 1958)  Mid childhood-exposed group (born between October 1st, 1954 and September 30th, 1956)  Late childhood-exposed group (born between October 1st, 1952 and September 30th, 1954) | Non-exposed group (birth year = 1962–1964)  Fetal-exposed group (birth year = 1959–1961)  Early childhood-exposed group (birth year = 1956–1958)  Mid childhood-exposed group (birth year = 1953–1955)  Late childhood-exposed group (, birth year = 1950–1952) |
| Types of studies | Historical cohort study | Historical Cohort study | Retrospective |
| sources of information | CHARLS |  |  |
| Country | China | China | China |
| Sample size | Unexposed = 1635  Fetal Exposed = 895  Early Childhood = 1218  Mid Childhood = 1364  Late Childhood = 1305  Total = 6417 | Non-exposed = 266  Fetal exposed = 210  Early childhood-exposed = 254  Mid childhood-exposed group = 227  Late childhood-exposed = 205  Total = 1162 |  |
| Sampling technique |  |  |  |
| Outcome studied | cognitive decline | Cognitive function | cognitive functioning |
| Outcome measurement/definition | comprehensive neuropsychological test (Telephone Interview of Cognitive Status (TICS-10), word recall, and pentagon drawing) | comprehensive neuropsychological battery test (Montreal cognitive Assessment-Beijing version, mini-mental state examination, auditory verbal learning test, digit span forward, digit span backward, trail making test, and digit symbol test) | Cognitive functioning tests (MMSE, MoCA, LMT, SCWT) |
| Key findings/Proportions/Mean | **General cognition**  Unexposed = 11.9 ± 3.7  Fetal Exposed = 11.2 ± 3.9  Early Childhood = 10.7 ± 4.0  Mid Childhood = 10.7 ± 4.0  Late Childhood = 10.2 ± 4.2 | **MoCA-BJ**  non-exposed 1964 = 25 (23, 27)  fetal exposed = 24 (22, 26)  early childhood-exposed = 25 (22, 27)  mid childhood-exposed group = 25 (22, 26)  late childhood-exposed = 25 (23, 27)  **MMSE**  non-exposed 1964 = 29 (27, 30)  fetal exposed = 28 (27, 29)  early childhood-exposed = 28 (27, 29)  mid childhood-exposed group = 28 (27, 29)  late childhood-exposed = 29 (27, 30) | **MoCA**  Non-exposed group = 26 (24, 28)  Fetal-exposed group = 26 (23, 27)  Early childhood-exposed group = 25 (22, 27)  Mid childhood-exposed group = 25 (23, 27)  Late childhood-exposed group = 25 (22, 27)  **MMSE**  Non-exposed group = 29 (28, 30)  Fetal-exposed group = 29 (27, 30)  Early childhood-exposed group = 29 (27, 30  Mid childhood-exposed group = 29 (27, 30)  Late childhood-exposed group = 28 (27, 29.5) |
| Effect Measures **(**95%CI OF AOR OR COR OR β-coefficients) | **General cognition**  Fetal Exposed = −1.05 (−1.64, −0.47)  Early Childhood = −0.75 (−1.71, 0.21)  Mid Childhood = −0.41 (−1.17, 0.34)  Late Childhood = −0.54 (−1.08, 0.01) | **(95% CI)**  MCI  fetal exposed = 1.43 (0.97–2.11)  early childhood-exposed = 1.48 (1.02–2.15)  mid childhood-exposed group = 1.40 (0.96–2.06)  late childhood-exposed = 1.44 (0.95–2.17)  **Global cognitive decline**  fetal exposed = 1.64 (1.00–2.68)  early childhood-exposed = 1.78 (1.10–2.84)  mid childhood-exposed group = 1.38 (0.84–2.82)  late childhood-exposed = 0.95 (0.54–1.70) | **MCI** = **OR (95%CI) ***  Fetal-exposed group = 1.14 (0.64–2.03)  Early childhood-exposed group = 1.17 (0.55–2.48)  Mid childhood-exposed group = 0.93 (0.37–2.34)  Late childhood-exposed group = 1.27 (0.43–3.76)  **DEMENTIA**= **OR (95%CI) ***  Fetal-exposed group = 1.96 (0.91–4.26)  Early childhood-exposed group = 2.01 (0.72–5.58)  Mid childhood-exposed group = 1.68 (0.48–5.88)  Late childhood-exposed group = 3.23 (0.74–14.01) |
| Comment | Early-life famine exposure in different stages is positively associated with late-life cognitive decline. Fetal famine exposure might affect the overall cognitive status in adulthood, and childhood famine exposure has potential adverse effects on visuospatial episodic memory. | The stronger associations were manifested in the people with high nutrient consumption pattern. The consumption of fat, carbohydrate and manganese were associated with multiple domains cognitive decline. | Famine exposure in utero and during childhood is associated with overall and specific cognitive decline, affecting selective attention and response inhibition particularly |
| Adjustment for covariates | age, gender, education, marital status, famine severity, drinking, activities of daily living, depression, self-reported general health status, health status in childhood, hypertension, and heart disease. | Age, sex, education, smoking, drinking, obesity, stroke, hypercholesterolemia, hypertension and HDL-C, dietary nutrient intake combination patterns | demographic and clinical characteristics |

| **Authors/year** | **(Xu et al., 2018)** | **(De Rooij et al., 2006a)** | **(de Rooij et al., 2006b)** |
| --- | --- | --- | --- |
| Participant characteristics | Rural participants born between 1958 and 1963 in | All singletons born alive between 1 November 1943 and 28 February 1947 in Wilhelmina Gasthuis, Amsterdam, | born as term singletons immediately before, during or after the 1944–1945 Dutch famine |
| Settings/context | Community study | women and men | women and men |
| Famine year/duration |  |  |  |
| Assessment of famine Exposure | date of birth | defined the famine period according to the daily official food rations | daily official food |
| Exposure groups | Pre-famine (1958 cohort)  Born during the famine period   - 1959 cohort - 1960 cohort - 1961 cohort - 1962 cohort   Post-famine (1963 cohort) | Exposed = born between 7 January and 8 December 1945  Late gestation = born between 7 January and 28 April 1945  Midgestation = born between 29 April and 18 August 1945)  Early gestation = born between 19 August and 8 December 1945  Unexposed = born before 7 January 1945 and conceived after 8 December 1945 | Exposed = born between 7 January and 8 December 1945  Late gestation = born between 7 January and 28 April 1945  Midgestation = born between 29 April and 18 August 1945)  Early gestation = born between 19 August and 8 December 1945  Unexposed = born before 7 January 1945 and conceived after 8 December 1945 |
| Types of studies | Historical cohort study | Historical Cohort study | Retrospective |
| sources of information | CHARLS | Dutch Famine Birth Cohort |  |
| Country | China | Dutch | Dutch |
| Sample size | Pre-famine (1958 cohort) = 404  Born during the famine period   - 1959 cohort = 327 - 1960 cohort = 307 - 1961 cohort = 291 - 1962 cohort = 522   Post-famine (1963 cohort) = 595  Total = 2446 | Born before famine = 19  Late gestation = 18  Midgestation = 18  Early gestation =18  Conceived after famine = 18  Total = 94 | Born before famine = 215  Late gestation = 122  Midgestation = 104  Early gestation = 65  Conceived after famine = 193  Total = 699 |
| Sampling technique |  |  |  |
| Outcome studied | cognitive performance | Impaired Insulin Secretion | Glucose tolerance comparison at age 58 and 50 |
| Outcome measurement/definition | Telephone Interview of Cognition Status (TICS), Episodic memory, Draw pentagons | World Health Organization recommendations, 199 | WHO |
| Key findings/Proportions/Mean | **General cognition**  Pre-famine (1958 cohort) = 11.37 (3.79)  Born during the famine period  1959 cohort = 11.40 (3.76)  1960 cohort = 11.81 (4.00)  1961 cohort = 11.55 (3.55)  1962 cohort = 12.18 (3.74)  Post-famine (1963 cohort) = 12.06 (3.50) | **Basal glucose (mmol/l)**  Born before famine = 5.1  late gestation = 4.8  Midge station = 5.0  Early gestation = 5.0  Conceived after famine = 5.0  **Glucose tolerance index**  Born before famine = 1.96  late gestation = 1.82  Midge station = 1.55*  Early gestation = 1.41*  Conceived after famine = 1.87  **Disposition index**  Born before famine = 1,576  late gestation = 1,539  Midge station = 1,059*  Early gestation = 1,252  Conceived after famine = 1,576 | **Fasting Glucose (mmol/l)**  Born before famine = 5.6  late gestation = 5.5  Midge station = 5.5  Early gestation = 5.6  Conceived after famine = 5.6  **HbA1c (%)**  Born before famine = 5.5  late gestation = 5.5  Midge station = 5.6  Early gestation = 5.6  Conceived after famine = 5.6  **120-min Glucose (mmol/l)**  Born before famine = 5.8  late gestation = 6.2  Midge station = 6.2  Early gestation = 6.2  Conceived after famine = 5.9  **Prevalence of diabetes based on OGTT (%)**  Born before famine = 5  late gestation = 5  Midge station = 5  Early gestation = 6  Conceived after famine = 4 |
| Effect Measures **(**95%CI OF AOR OR COR OR β-coefficients) | **OLS regression**  1958 cohort) = - 0.003 (-0.025, 0.018)  1959 cohort = 0.016 (-0.004, 0.036)  1960 cohort = - 0.018 (-0.044, 0.008)  1961 cohort = - 0.030 (-0.048, -0.011)  1962 cohort = - 0.007 (-0.026, 0.011) | **disposition index =** 95**% CI**  Prenatally exposed = - 21% [ - 41 to - 4]  Late gestation: -4% (-29 to 19)  Midgestation: -24% (-52 to -1)  Early gestation: -37% (-68 to -12) | **95% CIs**  **120-min glucose (mmol/l)**  Born before famine = 0.3 (0.0 to 0.6)  Late gestation = −0.1 (−0.6 to 0.2)  Midgestation = −0.1 (−0.5 to 0.3)  Early gestation = 0.2 (−0.4 to 0.8)  Conceived after famine = −0.1 (−0.4 to 0.3) |
| Comment | Severe nutritional deprivation during prenatal and postnatal periods has a lasting impact on cognitive performance in Chinese adults | Impaired glucose tolerance after exposure to famine during midge station and early gestation seems to be mediated through an insulin secretion defect | Although we confirmed that undernutrition during gestation is linked to decreased glucose tolerance, the effect does not seem to become more pronounced at age 58 as compared with age 50. |
| Adjustment for covariates | CSSI, gender, marital status, provincial fixed effects, education | sex and BM | Estimated marginal means (adjusted for sex and BMI) for differences |

| **Authors/year** | **(Li et al., 2010)** | **(Lumey et al., 2015)** | **(Meng et al., 2018)** |
| --- | --- | --- | --- |
| Participant characteristics | **Mean ages**  non- exposed = 39  fetal-exposed = 42  early childhood–exposed = 45  mid childhood–exposed = 47  late childhood–exposed = 49 | born between 1930 and 1938  patients with type 2 diabetes diagnosed at age 40 years or older | **Age at baseline, mean (SE)**  Non-exposed = 42.47 (0.01)  Fetal-exposed = 45.55 (0.01)  Early-childhood exposed = 48.45 (0.01) |
| Settings/context | Community study | women and men | women and men |
| Famine year/duration |  | 1932–33/2 years |  |
| Assessment of famine Exposure | subjects’ birth dates | Date of birth | All participants reported their date of birth at baseline |
| Exposure groups | Non-exposed = born between October 1, 1962, and September 30, 1964  Fetal exposed = born between October 1, 1959, and September 30, 1961  Early-childhood = born between October 1, 1956, and September 30, 1958  Mid-childhood = October 1, 1954 - September 30, 1956  Late-childhood = 1 October 1, 952 - September 30, 1954 | Included all patients with type 2 diabetes diagnosed at age 40 years or older in the Ukraine  Individuals born before and after the famine period as controls | Non-exposed (born between 1 October 1962 and 30 September 964)  Fetal-exposed (born between 1 October 1959 and 30 September 1961)  Early-childhood exposed (born between 1 October 1956 and 30 September 1958) |
| Types of studies | cross-sectional | retrospective cohort study | follow-up |
| sources of information | 2002 CNNHS | Ukraine national diabetes register 2000–08 |  |
| Country | China | Ukraine | China |
| Sample size | Non-exposed = 1,954  Fetal exposed = 1,005  Early-childhood = 1,654  Mid-childhood = 1,588  Late-childhood = 1,673  Total = 7874 | 43 150 patients with diabetes | Non-exposed = 38588  Fetal-exposed = 18879  Early-childhood exposed = 31363  Total = 88,8830 |
| Sampling technique |  |  |  |
| Outcome studied | Hyperglycemia and Type 2 Diabetes | type 2 diabetes | Joint effects of Prenatal famine exposure and adulthood obesity |
| Outcome measurement/definition | WHO Expert Committee on Diabetes Mellitus | WHO 1999 criteria | International Classification of Diseases (ICD-10) |
| Key findings/Proportions/Mean | **Fasting plasma glucose (mmol/l) ***  non- exposed cohort = 4.77 (0.03)  fetal-exposed cohort = 4.88 (0.05)  early childhood–exposed cohort = 4.87 (0.04)  mid childhood–exposed cohort = 4.88 (0.04)  late childhood–exposed = 4.99 (0.05)  **Hyperglycemia (%)**  Non- exposed cohort = 2.43  Fetal-exposed cohort = 5.65  Early childhood–exposed cohort = 3.93  Mid childhood–exposed cohort = 3.40  Late childhood–exposed = 5.89  **Type 2 diabetes (%)**  Non- exposed cohort = 1.57  Fetal-exposed cohort = 1.70  Early childhood–exposed cohort = 2.78  Mid childhood–exposed cohort = 1.69  Late childhood–exposed = 3.89 |  | Cases  **Whole cohort**  Non-exposed = 0.56%  Fetal-exposed = 0.35%  Early-childhood exposed = 0.62%  **HRs (95% CIs) for incident type 2 diabetes**  **Men**  Non-exposed = 0.47%  Fetal-exposed = 0.64%  Early-childhood exposed =0.59%  **HRs (95% CIs) for incident type 2 diabetes**  **Women**  Non-exposed = 0.81%  Fetal-exposed = 1.04%  Early-childhood exposed = 1.17% |
| Effect Measures **(**95%CI OF AOR OR COR OR β-coefficients) | **95% CI)**  **Hyperglycemia** (95% CI)  Fetal-exposed cohort = 3.92 (1.64–9.39)  Early childhood–exposed cohort = 1.77 (0.82–3.83)  Mid childhood–exposed cohort = 1.21 (0.57–2.55)  Late childhood–exposed = 2.38 (1.11–5.11)  **Type 2 diabetes** (95% CI)  Fetal-exposed cohort = 1.43 (0.53–3.87)  Early childhood–exposed cohort = 2.07 (0.82–5.24)  Mid childhood–exposed cohort = 0.75 (0.26–2.12)  Late childhood–exposed = 2.51 (0.91–6.87) | **OR** (95% CI) **for type 2 diabetes**  1·47 (95% CI 1·37–1·58) in individuals born in the first half of 1934 in regions with extreme famine  1·26 (1·14–1·39) in individuals born in regions with severe famine  there was no increase (OR 1·00, 0·91–1·09) in individuals born in regions with no famine | **HRs (95% CIs) for incident type 2 diabetes**  **Whole cohort**  Fetal-exposed = 1.21 (0.94–1.57)  Early-childhood exposed = 0.95 (0.67–1.36)  **HRs (95% CIs) for incident type 2 diabetes**  **Men**  Fetal-exposed = 1.27 (0.83–1.94)  Early-childhood exposed = 0.83 (0.46–1.51)  **HRs (95% CIs) for incident type 2 diabetes**  **Women**  Fetal-exposed = 1.15 (0.83–1.60)  Early-childhood exposed = 0.98 (0.62–1.54) |
| Comment | Fetal exposure to the severe Chinese famine increases the risk of hyperglycemia in adulthood. This association appears to be exacerbated by a nutritionally rich environment in later life | These results show a dose–response relation between famine severity during prenatal development and odds of type 2 diabetes in later life. the findings suggest that early gestation is a critical time window of development. The associations between type 2 diabetes and famine around the time of birth were similar in men and women | The association between general obesity and diabetes was consistent across subgroups according to famine exposure (P for interaction>0.05). A stronger association between abdominal obesity and diabetes was observed in the fetal-exposed subgroup than in other subgroups (P for interaction¼0.025 in the whole population). Coexistence of prenatal experience of undernutrition and abdominal obesity in adulthood was associated with a higher risk of type 2 diabetes. |
| Adjustment for covariates | Sex, education level, family history of diabetes, and current smoking, alcohol use, and physical activity level | Birth season, region of birth, and sex | Age, BMI and WHR |

| **Authors/year** | **(Ravelli et al., 1998)** | **(Stanner et al., 1997)** | **(Sun et al., 2018)** |
| --- | --- | --- | --- |
| Participant characteristics | Traced 5425 people born between Nov 1, 1943, and Feb 28, 1947 in the Wilhelmina Gasthuis, one of the principal hospitals in Amsterdam at that time for whom we had detailed prenatal and birth records  oral glucose load | **Age (years)**  Exposed = 52.3  Infant group = 53.1  Unexposed = 52.8  Subjects exposed to malnutrition in utero (intrauterine group) during the siege of Leningrad (now St Petersburg) | **Age in 2011/** **Age at the start of the famine (years)**  Fetal exposed = 49–52/ 0–4  Late exposed = 59–62/ 7–13  Middle exposed = 56–58/ 4–9  Early childhood = 53–55/ 1–6  Unexposed = 45–48/-  Residents who were born between 1 October 1949 and 1 July 1966 who had fasting plasma glucose (FPG) data, and who had never migrated from the province where they were born |
| Settings/context | Born in hospitals | Ott Institute of Obstetrics and Gynaecology, St Petersburg | women and men |
| Famine year/duration |  | 8 September 1941 and 27 January 1944 |  |
| Assessment of famine Exposure | Daily  rations for the general population | Date of birth | Birthdate was used as a proxy for famine exposure |
| Exposure groups | Non-exposed = before or conceived in the year after the famine  Exposed = exposed to famine at any stage during gestation | Exposed (intrauterine group) = born during the siege  Infant group = born in Leningrad just before rationing began, before the siege  Unexposed=subjects born concurrently with the first two groups but outside the area of the siege | Fetal exposed = (born between 1 January 1959 and 31 December 1962)  Late exposed = (born between 1 October 1949 and 31 December 1952)  Middle exposed = (born between 1 January 1953 and 31 December 1955)  Early childhood exposed = (born between 1 January 1956 and 31 December 1958)  Unexposed = (born between 1 January 1963 and 1 June 1966) |
| Types of studies | Historical | Cross sectional study | Cross sectional |
| Sources of information | Wilhelmina Gasthui hospitals | Register of the Society of Children of the Siege, which maintains a complete and updated record of all people living in, or born in, the city of Leningrad during the siege and  Historical records document the plentiful supply of food in Leningrad until rationing was imposed on 18 July 1941 in preparation for the impending siege. | CHARLS is a nationally representative longitudinal survey of Chinese middle-aged and elderly residents (≥45 years of age) supported by Peking University |
| Country | Dutch | Leningrad siege study | China |
| Sample size | Born before famine =202  Late gestation = 116  Mid gestation =100  Early gestation = 63  Conceived after famine =221  Total = 702 | Exposed (intrauterine group) = 169 during the siege  Infant group = 192 born in Leningrad just before rationing began, before the siege  Unexposed = 188 subjects born concurrently with the first two groups but outside the area of the siege  Total = 549 | Fetal exposed = 1389  Late exposed = 1499  Middle exposed = 1476  Early childhood = 1297  Unexposed = 1601  Total = 7,262 |
| Sampling technique |  |  |  |
| Outcome studied | Glucose tolerance | Risk factors for coronary heart disease and diabetes mellitus—obesity, blood pressure, glucose tolerance, insulin concentrations, lipids, albumin excretion rate, and clotting factors | type 2 diabetes mellitus and hyperglycemia |
| Outcome measurement/definition | WHO | WHO criteria  Electrocardiographic | WHO |
| Key findings/Proportions/Mean | **Fasting Glucose (mmol/L)**  Born before famine = 5·8  Late gestation = 5·8  Mid gestation = 5·7  Early gestation = 5·8  Conceived after famine = 5·6  **120 min Glucose (mmol/L)**  Born before famine = 5·7  Late gestation = 6·3  Mid gestation = 6·1  Early gestation = 6·1  Conceived after famine = 5·9  **Prevalence of IGT or type 2 diabetes**  Born before famine = 15%  Late gestation = 21%  Mid gestation = 14%  Early gestation = 16%  Conceived after famine = 15% | **Newly diagnosed diabetes**  Exposed = 1.8  Infant group = 0.5  Unexposed = 2.7  **Total cholesterol (mmol/l)**  Exposed = 5.5 (5.3 to 5.7)  Infant group = 5.5 (5.3 to 5.7)  Unexposed = 5.5 (5.4 to 5.6)  **Triglyceride (mmol/l)**  Exposed = 1.1 (0.6 to 2.2)  Infant group = 1.1 (0.6 to 2.2)  Unexposed = 1.0 (0.5 to 2.0)  **High density lipoprotein (mmol/l)**  Exposed = 1.3 (1.2 to 1.4)  Infant group = 1.4 (1.3 to 1.5)  Unexposed = 1. 4 (1.3 to 1.5)  **Low density lipoprotein (mmol/l)**  Exposed = 3.6 (1.5 to 5.7)  Infant group = 3.5 (3.3 to 3.7)  Unexposed = 3.6 (3.5 to 3.7)  **2-hour albumin excretion rate (g/min)**  Exposed = 4.5 (2.2 to 9.3)  Infant group = 4.7 (2.3 to 9.7)  Unexposed = 4.8 (2.3 to 9.9)  **Height (m)**  Exposed = 1.72  Infant group = 1.74  Unexposed = 1.73  **BMI, Male**  Exposed = 24.6  Infant group = 25.4  Unexposed = 25.2  **BMI, Female**  Exposed = 26.9  Infant group = 27.0  Unexposed = 26.7  **WHR, Male**  Exposed = 0.86  Infant group = 0.88  Unexposed = 0.87  **SBP (mm Hg)**  Exposed = 134.7  Infant group = 134.4  Unexposed = 130.9  **DBP (mm Hg)**  Exposed = 82.2  Infant group = 82.9  Unexposed = 79.0  **ECG**  Exposed = 2.7:1.5  Infant group = 3.2:2.3  Unexposed = 3.2:2.3  **Angina**  Exposed = 13.9:27.5  Infant group = 3.2:2.3  Unexposed = 14.0:21.9 | **Male, T2DM (%)**  Fetal exposed = 42.8  Late exposed = 38.9  Middle exposed = 41.1  Early childhood = 39.0  Unexposed = 47.0  **Male, Hyperglycemia (%)**  Fetal exposed = 72.0  Late exposed = 74.3  Middle exposed = 73.4  Early childhood = 70.6  Unexposed = 74.8  **Female, T2DM (%)**  Fetal exposed = 38.4  Late exposed = 38.9  Middle exposed = 37.8  Early childhood = 36.9  Unexposed = 36.5  **Female, Hyperglycemia (%)**  Fetal exposed = 70.7  Late exposed = 73.9  Middle exposed = 71.5  Early childhood = 71.7  Unexposed = 62.3 |
| Effect Measures **(**95%CI OF AOR OR COR OR β-coefficients) | **Glucose concentrations after 2 h glucose load**  Late gestation exposed = 0·5 mmol/L ( 0·1–0·9)  Mid gestation exposed = 0·4 mmol/L (0–0·8)  Early gestation exposed = 0·1 mmol/L (-0·4 to 0·6)  Unexposed = 5·8 mmol/L | **AOR (95% CI**  **glucose tolerance (mean fasting glucose)**  Intrauterine group = 5.2 (5.1 to 5.3)  Infant group = 5.3 (5.1 to 5.5)  **mean 2-hour glucose**  Intrauterine group = 6.1 (5.8 to 6.4)  Infant group = 6.0 (5.7 to 6.3) | **OR (95% CI),** Diabetes  Fetal exposed = 0.88 (0.71–1.09)  Late exposed = 0.88 (0.72–1.07)  Middle exposed = 0.86 (0.68–1.10)  Early childhood = 0.77 (0.62–0.96)  **Male, OR (95% CI),** Diabetes  Fetal exposed = 0.87 (0.66–1.15)  Late exposed = 0.74 (0.56–0.98)  Middle exposed = 0.86 (0.66–1.12)  Early childhood = 0.65 (0.49–0.86)  **Female, OR (95% CI),** Diabetes  Fetal exposed = 0.89 (0.67–1.17)  Late exposed = 1.02 (0.79–1.32)  Middle exposed = 0.89 (0.67–1.17)  Early childhood = 0.91 (0.69–1.19) |
| Comment | Poor nutrition in utero may lead to permanent changes in insulin-glucose metabolism, even if the effect on fetal growth is small. This effect of famine on glucose tolerance is especially important in people who become obese.  Glucose concentrations were increased 2 h after a standard glucose load among exposed participants (p=0·006) | Intrauterine malnutrition was not associated with glucose intolerance, dyslipidemia, hypertension, or cardiovascular disease in adulthood. Subjects exposed to malnutrition showed evidence of endothelial dysfunction and a stronger influence of obesity on blood pressure. Short adult stature was associated with raised concentrations of glucose and insulin 2 hours after a glucose load—independently of siege exposure. | Exposure to famine during early life can increase the risk of hyperglycemia in female adults, but may decrease the risk of T2DM in males. |
| Adjustment for covariates | birthweight, and body-mass index | adjustment for season of birth, sex | Gender, famine severity, self-reported family economic status, residential area type, health status before 16 years of age and residential area type before 16 years of age, body mass index, smoking status, alcohol consumption status, and waist circumference |

| **Authors/year** | **(Thurner et al., 2013)** | **(van Abeelen et al., 2012b)** | **(Wang et al., 2016b)** |
| --- | --- | --- | --- |
| Participant characteristics | Austrian patients that were under pharmaceutical treatment for diabetes during 2006 and 2007 | **Age at recruitment**  Unexposed = 59.0 (49–70)  Moderately exposed = 60.4 (49–70)  Severely exposed = 60.8 (49–70) | **Age, y**  Late child = 60.2 6± 0.6  Middle childhood = 58.1 ±6 0.6  Early-childhood = 56.1 ±6 0.6  Fetal-exposed = 53.1 6± 0.6  Unexposed groups = 50.0 ±6 0.6 |
| Settings/context | under treatment for diabetes | Women | women and men |
| Famine year/duration | The three major Famines: 1918–1919, 1938, 1946–1947 |  |  |
| Assessment of famine Exposure | year of birth | Self-reported famine exposure | birthdate and birthplace |
| Exposure groups | percentage of all diabetes patients in the total population specifically for each year of birth, from 1917 to 2007 | Childhood (age 0–9 years) =  Adolescence (age 10–17 years) =  Young adulthood (age >18 years) = | Unexposed = 1 October 1962 and 30 September 1964  Fetal-exposed = 1 October 1959 and 30 September 1961  Those born between 1 October 1952 and 30 September 1958 (before the famine) were classified into late-, middle-, and early- childhood– exposed groups by every 2 y. |
| Types of studies | Cross sectional | follow-up study | Cross sectional |
| Sources of information | Diabetes Patient Dataset | ProspectEPIC (European Prospective Investigation into Cancer and Nutrition) | CHARLS is a nationally representative longitudinal survey of Chinese middle-aged and elderly residents (≥45 years of age) supported by Peking University |
| Country | Austria | Dutch | China |
| Sample size | 325,000 | Unexposed = 3,572  Moderately exposed = 2,975  Severely exposed = 1,290  Total = 7,837 | Late child = 1953  Middle childhood = 1712  Early-childhood = 1932  Fetal-exposed = 1266  Unexposed groups = 938  Total = 7,801 |
| Sampling technique |  |  |  |
| Outcome studied | Type 2 Diabetes | Type 2 diabetes | type 2 diabetes mellitus |
| Outcome measurement/definition | WHO | WHO criteria  Electrocardiographic | WHO Diabetes Association criteria |
| Key findings/Proportions/Mean | 1918–1919 = ∼13% (16%) for a male (female)  1938 = 9% for males and 8% for females  1946–1947 = 5% for males and 3% for females. | Unexposed = 4.03%  Moderately exposed = 5.78%  Severely exposed = 7.05% | Prevalence of type 2 diabetes mellitus  Late child = 15.4%  Middle childhood = 16.5%  Early-childhood = 14.3%  Fetal-exposed = 12.0%  Unexposed = 12.3%  **Fasting plasma glucose**  Late child = 5.48  Middle childhood = 5.48  Early-childhood = 5.40  Fetal-exposed = 5.44  Unexposed = 5.31  **Glycated hemoglobin, %**  Late child = 5.49  Middle childhood = 5.46  Early-childhood = 5.41  Fetal-exposed = 5.34  Unexposed = 5.30  **Hyperglycemia, %**  Late child = 32.6  Middle childhood = 31.4  Early-childhood = 27.3  Fetal-exposed = 26.0  Unexposed = 23.6 |
| Effect Measures **(**95%CI OF AOR OR COR OR β-coefficients) |  | **HR 95% CI**  Moderately exposed = 1.28 (1.02–1.60)  Severely exposed = 1.52 (1.16–1.99)  **0–9 years, HR 95% CI**  Moderately exposed = 1.20 (0.83–1.74)  Severely exposed = 1.72 (1.13–2.62)  **10–17 years, HR 95% CI**  Moderately exposed = 1.39 (1.02–1.90)  Severely exposed = 1.40 (0.96–2.04)  > 18 years**, HR 95% CI**  Moderately exposed = 1.57 (0.74–3.33)  Severely exposed = 1.25 (0.47–3.33) | **AOR 95% CI, Type 2 diabetes**  Late child = 1.26 (0.97, 1.64)  Middle childhood = 1.44 (1.10, 1.87)  Early-childhood = 1.18 (0.91, 1.53)  Fetal-exposed = 1.03 (0.77, 1.38)  **AOR 95% CI, Hyperglycemia**  Late child = 1.54 (1.26, 1.88)  Middle childhood = 1.51 (1.23, 1.85)  Early-childhood = 1.21 (0.99, 1.48)  Fetal-exposed = 1.19 (0.96, 1.47) |
| Comment | We found a massive excess risk of diabetes in people born during the times of the three major famines and immediately after, which occurred in Austria in the 20th century. Depending on the region, there was an up to 40% higher chance of having diabetes when born in 1919–1921, compared with 1918 or 1922. The excess risk for diabetes was practically absent in those provinces of Austria that were less affected by the famines. Our results might be of relevance for establishing higher awareness in the health system for those born in high-risk years | a short period of moderate or severe undernutrition during postnatal development increases type 2 diabetes risk in adulthood. | Exposure to the Chinese famine in childhood was related to an increased risk of adulthood T2D and hyperglycemia, particularly in women |
| Adjustment for covariates |  | age at famine exposure, smoking, and level of education | Sex, BMI (in kg/m2), famine severity, drinking status, smoking status, metabolic equivalent, and family history of diabetes |

| **Authors/year** | **(van Abeelen et al., 2012a)** | **(Wang et al., 2017a)** | **(Wang et al., 2015)** |
| --- | --- | --- | --- |
| Participant characteristics | **Age at recruitment**  Unexposed = 59.0 (49–70)  Moderately exposed = 60.4 (49–70)  Severely exposed = 60.8 (49–70) | **Age, years**  Unexposed = 44  Fetal-exposed = 54± 1  Childhood-exposed = 62± 3  Adolescence/adult-exposed = 71± 4 | **Age in 2014, y**  Non-exposed = 40~51  Fetal-exposed = 52~55  Childhood-exposed = 56~65  Adolescence/adult-exposed = 66~93 |
| Settings/context | Women | women and men | women and men |
| Famine year/duration |  | 8 September 1941 and 27 January 1944 |  |
| Assessment of famine Exposure | Self-reported famine exposure | Self-reported famine exposure | birthdate and birthplace |
| Exposure groups | Childhood (age 0–9 years)  Adolescence (age 10–17 years)  Young adulthood (age >18 years) | Unexposed (born 1963−1997)  Fetal-exposed (born 1959−1962)  Childhood-exposed (born 1949−1958)  Adolescence/adult-exposed (born 1926−1948) | Non-exposed (born after 1975)  Non-exposed (born between 1963-1974)  Fetal-exposed (born between 1959-1962)  Childhood-exposed (born between 1949-1958)  Adolescence/adult-exposed (born between 1921-1948) |
| Types of studies | follow-up study | cross-sectional | Cross sectional |
| Sources of information | Prospect-EPIC cohort  National Medical Registry (hospital discharge diagnosis) | SPECT-China | SPECT-China |
| Country | Dutch | China | China |
| Sample size | Unexposed = 3577  Moderately exposed = 2,976  Severely exposed = 1292  = 7,845 | Unexposed (n=1632)  Fetal-exposed (n=489)  Childhood-exposed (n=1140)  Adolescence/adult-exposed (n=706)  Total = 3967 | Non-exposed = 1245  Non-exposed = 1808  Fetal-exposed = 745  Childhood-exposed = 1911  Adolescence/adult-exposed = 1188  Total = 6897 |
| Sampling technique |  |  |  |
| Outcome studied | coronary heart disease (CHD) and stroke | Diabetes | type 2 diabetes mellitus |
| Outcome measurement/definition | International Classification of Disease (ICD) | American Diabetes Association 2014 criteria | American Diabetes Association criteria |
| Key findings/Proportions/Mean | **CHD**  Unexposed = 8.27%  Moderately exposed = 7.99%  Severely exposed = 9.28%  **Stroke**  Unexposed = 3.15%  Exposed = 2.85% | **HbA1c, mmol/mol**  Unexposed = 37.0 ± 9.8  Fetal-exposed = 42.5 ± 12.7  Childhood-exposed = 43.6 ± 12.7  Adolescence/adult-exposed = 44.1 ± 13.0 | **Men, Diabetes, %**  Non-exposed = 2.7  Non-exposed = 10.6  Fetal-exposed = 18.1  Childhood-exposed = 18.3  Adolescence/adult-exposed = 16.0  **Women, Diabetes, %**  Non-exposed = 1.0b  Non-exposed = 5.0  Fetal-exposed = 8.7  Childhood-exposed = 14.8  Adolescence/adult-exposed = 21.3 |
| Effect Measures **(**95%CI OF AOR OR COR OR β-coefficients) | **CHD**  **HR 95% CI**  Moderately exposed = 1.08 (0.90–1.29)  Severely exposed = 1.15 (0.92–1.44)  **0–9 years, HR 95% CI**  Moderately exposed = 1.16 (0.87–1.56)  Severely exposed = 1.02 (0.69–1.52)  **10–17 years, HR 95% CI**  Moderately exposed = 1.02 (0.69–1.52)  Severely exposed = 1.02 (0.69–1.52)  > 18 years**, HR 95% CI**  Moderately exposed = 0.98 (0.76–1.26)  Severely exposed = 1.24 (0.92–1.67)  **Stroke, HR 95% CI**  Exposed = 0.78 (0.60–1.01)  **0–9 years, HR 95% CI**  Exposed = 0.70 (0.43–1.15)  **10–17 years, HR 95% CI**  Exposed = 0.86 (0.60–1.22)  **> 18 years, HR 95% CI**  Exposed = 0.63 (0.32–1.24) | **HbA1c, 95% CI**  Fetal-exposed = 0.31 (0.13, 051)  Childhood-exposed = 0.20 (0.05, 0.35)  Adolescence/adult-exposed = 0.27 (0.09. 0.44) | **95% CI, Total**  Non exposed (1975 and later) = 0.25 (0.13, 0.45)  Fetal exposed = 1.63 (1.13, 2.35)  Childhood exposed = 1.90 (1.22, 2.95)  Adolescence/adult exposed (1921–1948) = 1.95 (0.95, 3.98)  Non exposed (1963–1974) = 1.00 (Reference)  **Men, 95% CI**  Non exposed (1975 and later) = 0.30 (0.14, 0.67)  Fetal exposed = 1.74 (1.06, 2.84)  Childhood exposed = 1.40 (0.74, 2.66)  Adolescence/adult exposed (1921–1948) = 1.00 (0.35, 2.85)  Non exposed (1963–1974) =1.00 (Reference)  **Women, 95% CI**  Non exposed (1975 and later) = 0.22 (0.08, 0.58)  Fetal exposed = 1.51 (0.87, 2.62)  Childhood exposed = 2.61 (1.38, 4.92)  Adolescence/adult exposed (1921–1948) = 3.71 (1.37, 10.09)  Non exposed (1963–1974) =1.00 (Reference) |
| Comment | Exposure to undernutrition during postnatal periods of development, including adolescence, may affect cardiovascular health in adult life | Exposure to severe famine in the fetal or childhood period may predict a higher HbA1c and an increased diabetes risk in adulthood. Both the prenatal and postnatal period may offer critical time windows for the determination of the risk of diabetes. | The rapid increase in the prevalence of diabetes in middle-aged and elderly people in China is associated with the combination of exposure to famine during the fetal stage and childhood and high economic status in adulthood. |
| Adjustment for covariates | Age at famine exposure, smoking, and level of education as a proxy for socio-economic status | age, sex, current smoking status, education level and waist circumference | age, sex (only in total), rural/urban residence, and economic status, waist circumference, height, metabolic factors (LDL, HDL, triglycerides, and systolic BP). No interaction was found between life stages and economic status or between rural/urban residence and economic status. |

| **Authors/year** | **(Wang et al., 2018b)** | **(Zhang et al., 2018)** | **(Zhang et al., 2020)** |
| --- | --- | --- | --- |
| Participant characteristics | **Age (Mean ± SD, years)**  non-exposed = 48.84 ± 0.66  fetal-stage exposed = 51.83 ± 0.66  infant-stage exposed = 54.00 ± 0.00  preschool-stage exposed = 55.49 ± 0.50 | **Ages, Mean ± SD**  Early-childhood exposure = 54.52 ± 0.93  Fetal exposure =.58 ± 0.92  Transitional= 49.50 ± 0.50  Unexposed= 47.66 ± 0.96  50 years after early-life famine exposure | **Age**  Exposed group = 50.9 ± 1.0  Control group = 48.4 ± 0.8 |
| Settings/context | women and men | women and men | women and men |
| Famine year/duration |  |  |  |
| Assessment of famine Exposure | Birthdates of subjects | subjects’ dates of birth | birthdate and birthplace |
| Exposure groups | non-exposed = 10/1/1962–9/30/1964  fetal-stage exposed = 10/1/1959–9/30/1961  infant-stage exposed = 1/1/1958–12/31/1958  preschool-stage exposed = 1/1/1956–12/31/1957 | Early-childhood exposure = born between 1 January 1956 and 31 December 1958  Fetal exposure = born between 1 January 1959 and 31 December 1961  Transitional= born between 1 January 1962 and 31 December 1962  Unexposed= Born between 1 January 1963 and 31 December 1965 | Exposed group = born between 1 January 1960 and 31 December 1961  Control group = born between 1 January 1963 and 31 December 1964 |
| Types of studies | Retrospective | cross-sectional | Cross sectional |
| Sources of information | (CHARLS) 2011–2012 | SPECT-China | 2010–2012 CNHS |
| Country | China | China | China |
| Sample size | Non-exposed = 1536  fetal-stage exposed = 832  infant-stage exposed = 519  preschool-stage exposed = 1251  Total = 4138 | Early-childhood exposure = 1582  Fetal exposure = 1442  Transitional= 680  Unexposed= 1986  Total = 5690 | Exposed group = 4081  Control group = 3749  7830 |
| Sampling technique |  |  |  |
| Outcome studied | Diabetes mellitus | Hyperglycemia and Diabetes | Dysglycemiain |
| Outcome measurement/definition | American Diabetes Association | WHO criteria | WHO and IDF |
| Key findings/Proportions/Mean | non-exposed = 9.0%  fetal-stage exposed = 13.6%  infant-stage exposed = 12.7%  preschool-stage exposed = 10.8% | **Diabetes, n (%)**  Early-childhood exposure = 7.8  Fetal exposure = 8.5  Transitional= 7.1  Unexposed= 5.8  **Diabetes, Male n (%)**  Early-childhood exposure = 7.8  Fetal exposure = 8.0  Transitional= 7.8  Unexposed= 6.7  **Diabetes, Female n (%)**  Early-childhood exposure = 7.7  Fetal exposure = 8.9  Transitional= 6.4  Unexposed= 5.1  **Hyperglycemia**, n (%)  Early-childhood exposure = 10.0  Fetal exposure = 10.3  Transitional= 9.4  Unexposed= 9.1  **Hyperglycemia, Male n (%)**  Early-childhood exposure = 11.4  Fetal exposure = 12.9  Transitional= 12.5  Unexposed= 12.7  **Hyperglycemia, Female n (%)**  Early-childhood exposure = 8.8  Fetal exposure = 8.0  Transitional= 6.4  Unexposed= 6.2 | **Type 2 diabetes**  Exposed group = 6.4%  Control group = 5.1%  **Impaired glucose tolerance**  Exposed group = 5.3%  Control group = 5.0%  **Impaired fasting glucose**  Exposed group = 7.0%  Control group = 6.4% |
| Effect Measures **(**95%CI OF AOR OR COR OR β-coefficients) | Non-exposed = 1.58 (1.21–2.06)  Fetal-stage exposed = 0.92 (0.66–1.27)  Infant-stage exposed = 0.77 (0.59–1.01)  Preschool-stage exposed 1.37 (1.09–1.72)  **Sever Area**  Non-exposed = 1.70 (1.18–2.45)  Infant-exposed = 1.05 (0.69–1.61)  Preschool-exposed = 0.87 (0.61–1.25)  **Less, Sever Area**  Non-exposed = 1.33 (0.90–1.96)  Infant-exposed = 0.77 (0.45–1.31)  Preschool-exposed = 0.66 (0.43–1.01)  **Male**  Non-exposed = 1.51 (1.03–2.20)  Infant-exposed = 0.76 (0.47–1.24  Preschool-exposed = 0.66 (0.44–0.97)  **Female**  Non-exposed = 1.72 (1.18–2.50)  Infant-exposed = 1.01 (0.63–1.60)  Preschool-exposed = 0.86 (0.59–1.26) | **Diabetes**  Early-childhood exposure = 1.22 (0.92–1.61)  Fetal exposure = 1.40 (1.06–1.85)  Transitional= 1.16 (0.80–1.67)  **Diabetes, Male**  Early-childhood exposure = 1.18 (0.80–1.76)  Fetal exposure = 1.10 (0.74–1.65)  Transitional= 1.11 (0.68–1.83)  **Diabetes, Female**  Early-childhood exposure = 1.22 (0.81–1.82)  Fetal exposure = 1.67 (1.12–2.49)  Transitional= 1.18 (0.69–2.03)  **Hyperglycemia**  Early-childhood exposure = 1.17 (0.93–1.47)  Fetal exposure = 1.15 (0.91,1.45)  Transitional= 1.02 (0.75–1.38)  **Hyperglycemia, Male**  Early-childhood exposure = 0.93 (0.68–1.27)  Fetal exposure = 1.01 (0.75–1.37)  Transitional= 0.96 (0.66–1.42)  **Hyperglycemia, Female**  Early-childhood exposure = 1.55 (1.10–2.19)  Fetal exposure = 1.35 (0.94–1.94)  Transitional= 1.07 (0.65–1.76) | ORs (95%CI)  Type 2 diabetes  Exposed group = 1.23(1.01,1.50)  Impaired glucose tolerance  Exposed group = 1.06(0.86,1.31)  Impaired fasting glucose  Exposed group = 1.10(0.91,1.33) |
| Comment | Famine exposure in fetal stages was associated with the elevated diabetes risk in adults, which could be the critical periods for relative intervention.  use age-balanced control group | Early-life exposure to famine increased the risk of diabetes. Furthermore, early-childhood exposure to famine might increase the risk of hyperglycemia in women. A policy for preventing early life malnutrition should be drafted by the government to prevent hyperglycemia and diabetes in adulthood | fetal exposure to Chinese famine increased the risk of dysglycemia in adulthood. This association was stronger in the severely affected area and females. |
| Adjustment for covariates | gender, BMI, smoking status, drinking status, physical activity level, the highest education attainments of participants and their parents. | BMI, region, education, smoking status, drinking status, physical activity, and fruit intake | gender, economic status, education level, physical exercise, sedentary time, smoking, drinking, dietary factors, BMI. |

| **Authors/year** | **(Zhang et al., 2019)** | **(Chen et al., 2019)** | **(Ding et al., 2020)** |
| --- | --- | --- | --- |
| Participant characteristics | **Age in 2011 (years)**  Late childhood = 57.48 ± 0.59  Mid childhood = 55.51 ± 0.57  Early childhood = 53.51 ± 0.55  Foetal exposure = 50.57 ±0.57  No exposure = 47.53 ±0.58 | **Age in 2014**  fetal-exposed = 52–55  childhood-exposed = 56–65  adolescence exposed = 66–93  non-exposed = 40–51 | **Age, years, mean (SD)**  Unexposed = 48.23 (0.9)  Postnatal-Exposed= 51.61 (0.8)  Prenatal-Exposed= 50.60 (0.8) |
| Settings/context | women and men | women and men | women and men |
| Famine year/duration |  |  |  |
| Assessment of famine Exposure | Birthdates of subjects | subjects’ dates of birth | birthdate and birthplace |
| Exposure groups | Late childhood = 1 October, 1952 to 1964, 30 September  Mid childhood = 1 October, 1954 to 1961, 30 September  Early childhood = 1 October, 1956 to 1958, 30 September  Foetal exposure = 1 October, 1959 to 1961, 30 September  No exposure = 1 October, 1962 to 1964, 30 September | fetal-exposed (1959–1962)  childhood-exposed (1949–1958)  adolescence/young adultexposed (1921–1948)  non-exposed (1963–1974) groups | Unexposed = 1962.10.01–1964.09.30  Exposure during early postnatal = 1959.10.01–1960.09.30  Exposure during prenatal = 1960.10.01–1961.09.30 |
| Types of studies | Retrospective | cross-sectional | Cross sectional |
| Sources of information | CHARLS | SPECT-China | CNHS 2010–2012 |
| Country | China | China | China |
| Sample size | Late childhood = 952  Mid childhood = 939  Early childhood = 864  Foetal exposure = 568  No exposure = 1014  4337 | Early-childhood exposure = 1582  Fetal-exposed = 706  Childhood-exposed = 1799  Adolescence exposed = 1064  Non-exposed = 1726 | Unexposed = 5832  Exposure during early postnatal = 2068  Exposure during prenatal = 1592 |
| Sampling technique |  |  |  |
| Outcome studied | Modifies the effect of hyperglycaemia on cardiovascular disease (CVD). | visceral adipose dysfunction | Lipid Profiles |
| Outcome measurement/definition | self-reported doctor’s diagnosis of stroke or heart problems (including heart attack, coronary heart disease (CHD), angina, congestive heart failure or other heart problems) | VAI was calculated using the following sex-specific equations | WHO and IDF |
| Key findings/Proportions/Mean | **CVD (%)**  Late childhood = 15.24  Mid childhood = 12.53  Early childhood = 13.55  Foetal exposure = 11.86  No exposure = 10.18  **Hyperglycaemia (%)**  Late childhood = 34.77  Mid childhood = 32.37  Early childhood = 31.48  Foetal exposure = 31.34  No exposure = 24.85 | **Visceral adiposity index, Men**  Fetal-exposed = 1.47 (0.94–2.39)  Childhood-exposed = 1.29 (0.85–2.05)  Adolescence exposed = 1.07 (0.70–1.72)  Non-exposed = 1.50 (0.97–2.51)  **Visceral adiposity index, Women**  Fetal-exposed = 1.57 (1.06–2.46)  Childhood-exposed = 1.67 (1.11–2.66)  Adolescence exposed = 1.71 (1.14–2.63)  Non-exposed = 1.25 (0.85–1.87) | **TG, mmol/L, mean (SD)**  Unexposed = 1.52 (1.1)  Postnatal-Exposed= 1.56 (1.1)  Prenatal-Exposed= 1.58 (1.2)  **TC, mmol/L, mean (SD)**  Unexposed = 4.67 (1.0)  Postnatal-Exposed= 4.78 (1.0)  Prenatal-Exposed= 4.73 (1.0)  **HDL-C, mmol/L, mean (SD)**  Unexposed = 1.18 (0.3)  Postnatal-Exposed= 1.20 (0.3)  Prenatal-Exposed= 1.16 (0.3)  **LDL-C, mmol/L, mean (SD)**  Unexposed = 2.79 (0.8)  Postnatal-Exposed= 2.87 (0.8)  Prenatal-Exposed= 2.85 (0.8)  **TC/HDL-C, mean (SD)**  Unexposed = 4.23 (1.5)  Postnatal-Exposed= 4.26 (1.5)  Prenatal-Exposed= 4.40 (1.7)  **TG/HDL-C, mean (SD)**  Unexposed = 1.58 (2.0)  Postnatal-Exposed= 1.60 (1.9)  Prenatal-Exposed= 1.70 (2.2)  **Overweight, (%)**  Unexposed = 37.35  Postnatal-Exposed = 37.61  Prenatal-Exposed = 39.77  **Obesity, (%)**  Unexposed = 14.72  Postnatal-Exposed = 15  Prenatal-Exposed = 13.15  **Central obesity, (%)**  Unexposed = 31.75  Postnatal-Exposed = 33.85  Prenatal-Exposed = 33.92  **BMI, kg/m2, mean (SD)**  Unexposed = 24.43 (3.4)  Postnatal-Exposed = 24.48 (3.4)  Prenatal-Exposed = 24.41 (3.4) |
| Effect Measures **(**95%CI OF AOR OR COR OR β-coefficients) | OR (95% CI), Overall (Early Life)  1.27 (1.12,1.44)  **OR (95% CI)**  Late childhood = 1.46 (0.94,2.26)  Mid childhood = 1.76 (1.06,2.90)  Early childhood = 1.40 (0.86,2.27)  Foetal exposure = 2.55 (1.30,5.02)  No exposure = 1.10 (0.63,1.95) | Men  Fetal-exposed = − 0.02 (− 0.13, 0.10)  Childhood-exposed = − 0.08 (− 0.23, 0.06)  Adolescence exposed = − 0.09 (− 0.32, 0.15)  Women  Fetal-exposed = 0.17 (0.08, 0.26)  Childhood-exposed = 0.13 (0.02, 0.24)  Adolescence exposed = 0.02 (− 0.16, 0.21) | **Elevate TG, (95% CI)**  Postnatal-Exposed= 1.07 (0.90, 1.26)  Prenatal-Exposed= 1.06 (0.88, 1.27)  **Elevate TC, (95% CI)**  Postnatal-Exposed= 1.60 (1.27, 2.02)  Prenatal-Exposed= 1.22 (0.92, 1.60)  **Low HDL-C, (95% CI)**  Postnatal-Exposed= 0.92 (0.80, 1.05)  Prenatal-Exposed= 1.19 (1.03, 1.37)  **Elevate LDL-C**  Postnatal-Exposed= 1.37 (1.09, 1.73)  Prenatal-Exposed= 1.02 (0.77, 1.34)  **Dyslipidemia, (95% CI)**  Postnatal-Exposed= 0.96 (0.85, 1.10)  Prenatal-Exposed= 1.20 (1.04, 1.38) |
| Comment | Adverse long-term effects of famine might be more apparent in the coming decade with ageing of the  exposed populations. There was a significant interaction between hyperglycaemia and famine exposure for CVD (P Z 0.0374). The effect of hyperglycaemia on CVD in the foetal exposure cohort was significantly higher than those in any of the other famine-exposed cohorts, especially in those who lived in rural areas, lived in severe famine areas and those who were men. Exposure to the Chinese Famine, especially during the foetal stage of life, aggravated the association between hyperglycaemia and CVD | Visceral adiposity index (VAI) is a new method for the assessment of adipose distribution and function. WC is traditional, does not distinguish between visceral and subcutaneous fat. Exposure to famine in early life may have a significant association with visceral adipose dysfunction in adult females. The fetal age and childhood may be important time windows for nutrition relief to prevent visceral adipose dysfunction. | Famine during early life, especially in females and people physical inactivity, would impair of lipid profiles in later life. Healthy lifestyle like adequate physical activity may partially alleviate the adverse effects. |
| Adjustment for covariates | age, sex, smoking, alcohol drinking, education, residence, BMI, famine severity, hypertension and dyslipidaemia | age, smoking, rural/urban residence, economic status, diabetes and hypertension | gender, age, obesity status, abdominal obesity or not, education level, marital status, per-capita annual income, exercise, working strength, control lipid plasma by drugs or others and severity of famine |

| **Authors/year** | **(Lumey et al., 2009)** | **(Lussana et al., 2008)** | **(Roseboom et al., 2000a)** |
| --- | --- | --- | --- |
| Participant characteristics | **Age (y)**  Hospital controls = 58.6 ± 6 1.6  Sibling controls = 57.2 ± 6.4  Famine exposed = 58.7 ± 0.4 | men and women (aged 58 y) born in the Wilhelmina Gasthuis in Amsterdam, | persons aged = 50 y |
| Settings/context | singleton men and women born in clinics whose mothers were exposed to the famine during pregnancy | women and men | men and women born at term as singletons in a university hospital in Amsterdam between 1 November 1943 and 28 February 1947 around the time of a severe famine. |
| Famine year/duration |  |  |  |
| Assessment of famine Exposure | official ration of < 900 kcal/d between 26 November 1944 and 12 May 1945. | average daily rations of the mothers during gestation: < 1000 kcal (4200 kJ)/d | official food rations for the general population aged >21 y |
| Exposure groups | Exposed = born between January 1945 and March 1946 in clinics in Amsterdam  Unexposed = born in the same institutions as exposed individuals but before the famine or after the famine and not exposed to famine during gestation | Prenatally exposed = born between 7 January 1945 and 8 December 1945  Late gestation (born between 7 January and 28 April 1945)  Midgestation (born between 29 April and 18 August 1945)  Early gestation (born between 19 August and 8 December 1945)  Unexposed = born before 7 January 1945 and persons conceived after 8 December 1945 | Exposed = born between January 1945 and March 1946 in clinics in Amsterdam  Unexposed = born in the same institutions as exposed individuals but before the famine or after the famine and not exposed to famine during gestation |
| Types of studies | Retrospective | retrospective | follow-up study |
| Sources of information | Names and addresses at birth for the 3307 infants were provided to the Population Register in the municipality of birth with a request for tracing to their current address | Medical birth records | Amsterdam (population registry) |
| Country | Dutch | Dutch | Dutch |
| Sample size | Hospital controls = 294  Sibling controls = 308  Famine exposed = 344  Total = 946 | Born before famine= 228  Late gestation = 125  Mid gestation = 106  Early gestation = 63  Conceived after famine = 208 | Born before famine = 199  Late gestation = 118  Mid gestation = 101  Early gestation = 64  Conceived after famine **=** 222 |
| Sampling technique |  |  |  |
| Outcome studied | Lipid profiles | preference for fatty foods and a more atherogenic lipid profile | Plasma lipid profiles |
| Outcome measurement/definition |  |  | WHO and IDF |
| Key findings/Proportions/Mean | **dyslipidemia (%)**  Hospital controls = 29  Sibling controls = 24  Famine exposed = 28 | **Total cholesterol (mmol/L)**  Born before famine= 5.9 ± 1.0  Late gestation = 5.8 ±1.1  Mid gestation = 5.8 ±1.0  Early gestation = 6.0 ± 1.2  Conceived after famine = 5.8 ±1.1  **HDL cholesterol (mmol/L)**  Born before famine= 1.6 ±0.4  Late gestation = 1.5 ± 0.4  Mid gestation = 1.5±0.4  Early gestation = 1.5 ±0.5  Conceived after famine = 1.5 ± 0.4  **LDL cholesterol (mmol/L)**  Born before famine= 3.67 ± 0.9  Late gestation = 3.67 ± 0.9  Mid gestation = 3.57 ± 1.0  Early gestation = 3.82 ±1.1  Conceived after famine = 3.6 ±1.1  **Triglycerides (g/L)**  Born before famine= 1.42 ± 0.9  Late gestation = 1.52 ± 0.9  Mid gestation = 1.5 ± 0.9  Early gestation = 1.6 ± 0.9  Conceived after famine = 1.6 ± 0.9  **LDL: HDL**  Born before famine= 2.5 ± 0.9  Late gestation = 2.7 ± 0.9  Mid gestation = 2.6 ± 1.1  Early gestation = 2.9 ± 1.1  Conceived after famine = 2.5 ± 0.9 | **Total cholesterol (mmol/L)**  Born before famine = 6.06  Late gestation = 5.83  Mid gestation = 5.80  Early gestation = 6.13  Conceived after famine = 6.00  **HDL cholesterol (mmol/L)**  Born before famine = 1.35  Late gestation = 1.32  Mid gestation = 1.37  Early gestation = 1.26  Conceived after famine = 1.32  **LDL cholesterol (mmol/L)**  Born before famine = 4.05  Late gestation = 3.87  Mid gestation = 3.81  Early gestation = 4.26  Conceived after famine = 4.02  **Triacylglycerol (g/L)**  Born before famine = 1.15  Late gestation = 1.08  Mid gestation = 1.10  Early gestation = 1.10  Conceived after famine = 1.16  **LDL: HDL cholesterol**  Born before famine = 2.91  Late gestation = 2.82  Mid gestation = 2.69  Early gestation = 3.26  Conceived after famine = 2.94 |
| Effect Measures **(**95%CI OF AOR OR COR OR β-coefficients) | **TC (mmol/L)**  Famine-exposed men = - 0.11 (-0.30, 0.09)  Famine-exposed women = 0.26 (0.07, 0.46)  **HDL cholesterol (mmol/L)**  Famine-exposed men = 0.03 (-0.05, 0.10)  Famine-exposed women = 0.00 (-0.08, 0.08)  **Triglycerides (mmol/L)**  Famine-exposed men = 0.06 (-0.16, 0.29)  Famine-exposed women = 0.17 (0.03, 0.31)  **LDL cholesterol (mmol/L)**  Famine-exposed men = -0.18 (-0.35, -0.01)  Famine-exposed women = 0.17 (-0.01, 0.35)  **TC: HDL cholesterol**  Famine-exposed men = -0.13 (-0.42, 0.17)  Famine-exposed women = 0.13 (-0.09, 0.34)  **LDL cholesterol:HDL cholesterol**  Famine-exposed men = -0.21 (-0.40, -0.02)  Famine-exposed women = 0.07 (-0.10, 0.24) | **Effect size of early exposure on lipid level**  Total cholesterol = 0.12 (Ҁ0.27, 0.517)  LDL cholesterol = 0.20 (0.15, 0.55)  LDL: HDL = 0.07 (0.01, 0.12) | Late gestation = - 0.20 (- 0.41, 0.02)  Mid gestation = - 0.23 (- 0.46, 0.00)  Early gestation = 0.10 (- 0.18, 0.38)  **HDL cholesterol (mmol/L)**  Late gestation = -2.0 (-6.9, 3.1)  Mid gestation = 0.0 (-5.4, 5.6)  Early gestation = -7.0 (-13.0, -0.6)  **LDL cholesterol (mmol/L)**  Late gestation = -0.15 (-0.36, 0.05)  Mid gestation = -0.21 (-0.42, 0.01)  Early gestation = 0.24 (-0.02, 0.51)  **Triacylglycerol (g/L)**  Late gestation = -5.5 (-15.1, 5.3)  Mid gestation = -2.7 (-13.4, 9.2)  Early gestation = -3.7 (-16.2, 10.7)  **LDL: HDL cholesterol**  Late gestation = -2.5 (-10.1, 5.6)  Mid gestation = -5.3 (-13.1, 3.3)  Early gestation = 13.9 (2.6, 26.3) |
| Comment | In women, but not in men, aged ’58 y, we observed an association between prenatal undernutrition and elevated total cholesterol concentrations and triglycerides | Prenatal nutrition affects dietary preferences and may contribute to more atherogenic lipid profiles in later life | An atherogenic lipid profile might be linked to a transition from poor maternal nutrition in early gestation to adequate nutrition later on. This suggests that maternal malnutrition during early gestation may program lipid metabolism without affecting size at birth |
| Adjustment for covariates | Age, sex, BMI; in kg/m2), waist circumference, education, current smoking habit, alcohol use, and prevalent hypertension. | sex, BMI, SES, and lipid-lowering medication and fat intake | adjusted for sex, between participants prenatally exposed to famine (in late, mid, or early gestation) and nonexposed participants (those born before or conceived after the famine) |

| **Authors/year** | **(Wang et al., 2017c)** | **(Xin et al., 2019)** | **(Yao and Li, 2019)** |
| --- | --- | --- | --- |
| Participant characteristics | **Age mean(SD) year**  Non-Exposed = 47.37 (0.48)  Fetal Stage-Exposed = 50.91 (0.67)  Infant Stage-Exposed = 53.17 (0.37)  Preschool Stage-Exposed = 55.14 (0.35) | Age (years)  Fetal-infant exposed group = 49.32 ± 1.07  Childhood exposed group = 55.61 ± 2.80  Adolescence exposed group = 64.45 ± 2.37  Unexposed group = 45.37 ± 1.46 | born from 1955 to 1965 received a health checkup  51–55-year-old residents were born during the years of the famine |
| Settings/context | women and men | women and men | women and men |
| Famine year/duration |  |  |  |
| Assessment of famine Exposure | birthdates of participants | subjects’ dates of birth | birthdate and birthplace |
| Exposure groups | Non-Exposed = (10/01/1962–09/30/ 1964)  Fetal Stage-Exposed = (10/01/1959–09/ 30/1961)  Infant Stage-Exposed = (01/01/1958– 12/31/1958)  Preschool Stage-Exposed = (01/01/ 1956–12/31/1957) | Fetal-infant exposed group (born between 01/01/1959 and 12/31/1961, exposed prenatally and up to the age of 2 years)  Childhood exposed group (born between 01/01/1949 and 12/31/1958, the age of exposed ranged from 3 to 12 years)  Adolescence exposed group (born between 01/01/1941 and 12/31/1948, the age of exposed ranged from 13 to 20 years); and (4) unexposed group (born between 01/01/1962 and 12/31/1966) | Early childhood-exposure = born between January 1, 1955 and December 31, 1957  Fetal-exposure group = born between January 1, 1959 and December 30, 1961  non-exposed group = born between October 1, 1963 and September 30, 1965 |
| Types of studies | cross-sectional | Historic cohort study | Cross sectional |
| Sources of information | CHARLS | data from CHNS 2009 | participants who underwent a health examination at Hefei Tertiary Hospital |
| Country | China | China | China |
| Sample size | Non-Exposed = 822  Fetal Stage-Exposed = 797  Infant Stage-Exposed = 536  Preschool Stage-Exposed = 597  Total = 2752 | Fetal-infant exposed group = 433  Childhood exposed group = 2132  Adolescence exposed group = 1140  Unexposed group = 1138 | Early childhood-exposure = 206  Fetal-exposure group = 127  non-exposed group = 271  Total = 604 |
| Sampling technique |  |  | cluster sampling |
| Outcome studied | Dyslipidemia | Dyslipidemia | Dyslipidemia |
| Outcome measurement/definition | WHO | Chinese adult dyslipidemia prevention guide (2016 edition): | Chinese adult dyslipidemia prevention guide (2016 edition) |
| Key findings/Proportions/Mean | **Dslipidemia (%)**  Non-Exposed = 15.7  Fetal Stage-Exposed = 23.1  Infant Stage-Exposed = 22.0  Preschool Stage-Exposed = 18.6 | **Dyslipidemia, %**  Fetal-infant exposed group = 64.00  Childhood exposed group = 63.90  Adolescence exposed group = 63.90  Unexposed group = 56.40  **Urban, Dyslipidemia, %**  Fetal-infant exposed = 64.70  Childhood exposed = 63.70  Adolescence exposed = 66.50  Unexposed = 58.90  **Rural, Dyslipidemia, %**  Fetal-infant exposed = 63.50  Childhood exposed = 64.00)  Adolescence exposed = 62.50  Unexposed = 55.40  **TC (mmol/L), Urban**  Fetal-infant exposed = 5.00 (1.37) *  Childhood exposed = 4.95 (1.24) *  Adolescence exposed = 5.04 (1.19) *  Unexposed = 4.79 (1.25)  **TC (mmol/L), Rural**  Fetal-infant exposed = 4.88 (1.31) *  Childhood exposed = 5.00 (1.35) *  Adolescence exposed = 4.97 (1.33) *  Unexposed = 4.74 (1.22)  **TG (mmol/L), Urban**  Fetal-infant exposed = 1.33 (1.42)  Childhood exposed = 1.38 (1.15)  Adolescence exposed = 1.38 (1.29)  Unexposed = 1.32 (1.43)  **TG (mmol/L), Rural**  Fetal-infant exposed = 1.41 (1.26) *  Childhood exposed = 1.35 (1.20) *  Adolescence exposed = 1.31 (1.08)  Unexposed = 1.26 (1.09)  **LDL (mmol/L), Urban**  Fetal-infant exposed = 3.09 (1.16) *  Childhood exposed = 3.07 (1.24) *  Adolescence exposed = 3.14 (1.09) *  Unexposed = 2.85 (1.11)  **LDL (mmol/L), Rural**  Fetal-infant exposed = 2.96 (1.13)  Childhood exposed = 3.08 (1.25) *  Adolescence exposed = 3.06 (1.27) *  Unexposed = 2.87 (1.11)  **HDL (mmol/L), Urban**  Fetal-infant exposed = 1.32 (0.52)  Childhood exposed = 1.36 (0.45)  Adolescence exposed = 1.37 (0.43)  Unexposed = 1.33 (0.44)  **HDL (mmol/L), Rural**  Fetal-infant exposed = 1.37 (0.53)  Childhood exposed = 1.43 (0.49) *  Adolescence exposed = 1.41 (0.49)  Unexposed = 1.40 (0.49) | **TAG (mmol/L)**  non-exposed group = 2.14 (1.96)  Fetal-exposure group = 2.12 (1.71)  Early childhood-exposure = 2.02 (1.51)  **TC (mmol/L)**  non-exposed group = 5.27 (0.92)  Fetal-exposure group = 5.50 (1.02)  Early childhood-exposure = 5.44 (0.92)  **HDL-C (mmol/L)**  non-exposed group = 1.66 (0.50)  Fetal-exposure group = 1.76 (0.60)  Early childhood-exposure = 1.62 (0.37)  **LDL-C (mmol/L)**  non-exposed group = 2.71 (0.75)  Fetal-exposure group = 2.90 (0.90)  Early childhood-exposure = 2.92 (0.76) |
| Effect Measures **(**95%CI OF AOR OR COR OR β-coefficients) | Odds ratio (95% CI)  Fetal Stage-Exposed = 1.62 (1.27–2.09)  Infant Stage-Exposed = 1.53 (1.16–2.02)  Preschool Stage-Exposed = 1.23 (0.93–1.62) | **Adjusted OR (95% CI)**  Fetal-infant exposed = 1.34 (1.05–1.70)  Childhood exposed = 1.44 (1.23–1.69)  Adolescence exposed = 1.41 (1.17–1.71)  **Urban, Dyslipidemia**  Fetal-infant exposed = 1.18 (0.77–1.80)  Childhood exposed = 1.23 (0.92–1.63)  Adolescence exposed = 1.25 (0.89–1.76)  **Rural, Dyslipidemia**  Fetal-infant exposed = 1.39 (1.03–1.87)  Childhood exposed = 1.55 (1.28–1.88)  Adolescence exposed = 1.49 (1.18–1.87) | **Elevated TC, OR (95% CI)**  Fetal-exposure group = 1.90 (1.05, 3.43)  Early childhood-exposure = 1.19 (0.69, 2.07)  **Low HDL-C, OR (95% CI)**  Fetal-exposure group = 7.11 (0.13, 392.60)  Early childhood-exposure = 6.66 (0.12, 382.71)  **Elevated TAG, OR (95% CI)**  Fetal-exposure group = 0.87 (0.52, 1.45)  Early childhood-exposure = 0.68 (0.43, 1.06)  **Elevated LDL-C, OR (95% CI)**  Fetal-exposure group = 5.00 (1.59, 15.77)  Early childhood-exposure = 2.52 (0.81, 7.85)  **dyslipidemia, OR (95% CI)**  Fetal-exposure group = 1.37 (0.86, 2.20)  Early childhood-exposure = 0.75 (0.49, 1.15)  **Male, dyslipidemia, OR (95% CI)**  Fetal-exposure group = 0.96 (0.52, 1.80)  Early childhood-exposure = 0.90 (0.54, 1.50)  **Female, dyslipidemia, OR (95% CI)**  Fetal-exposure group = 2.00 (1.03, 3.86)  Early childhood-exposure = 0.96 (0.52, 1.77) |
| Comment | Early-life exposure to severe Chinese famine could link with the higher dyslipidemia risk in female adulthood, but not in male adulthood. This gender-specific effect might be associated with the hypothesis that parents in China prefer boys to girls traditionally or survivors’ bias. | Exposure to Chinese famine in early life was associated with increased risk of dyslipidemia in adulthood. Preventing undernutrition in early life is an appropriate recommendation to reduce the prevalence of later dyslipidemia. | Exposure to famine in early life leads to altered lipid distribution in adulthood, and the risk of dyslipidemia significantly increased in adult women who were exposed to famine during the fetal period.  This study provides a scientific basis for the prevention and control of abnormal blood lipid levels in adults. |
| Adjustment for covariates | gender and current family economic status | sex, smoking, drinking, BMI, hypertension, diabetes, education | gender, diabetes, hypertension, BMI, occupation, education, smoking, alcohol drinking, exercise frequency, sleeping time, birth weight, feeding methods, waist-to-hip ratio, and dietary pattern. |

| **Authors/year** | **(Chen et al., 2014)** | **(Hult et al., 2010)** | **(Koupil et al., 2007)** |
| --- | --- | --- | --- |
| Participant characteristics | **Age, Mean (SD)**  Unexposed = 46.07 (0.60)  Fetal exposed = 49.16 (0.59)  Early child exposed = 52.03 (0.62) | **Age, Mean**(SD)  Early childhood = 43.0 (0.8)  Fetal-infant = 40.5 (0.6)  Unexposed = 37.0 (0.8)  Transitional period = 39(0.0) | **Age at examination (years)**  Born 1910–1915 = 68.5  Born 1916–1925 = 58.7  Born 1926–1932 = 51.1  Born 1933–1935 = 46.1  Born 1936–1940 = 41.9  aged 1–5, 6–8, 9–15, 16–25 and 26–31 years when exposed to the severest starvation. |
| Settings/context | women and men | women and men | surviving men and women |
| Famine year/duration |  | 6 July 1967 - 15 January 1970 | 1941–1944 |
| Assessment of famine Exposure | birthdates of participants | subjects’ dates of birth | year of birth |
| Exposure groups | Fetal-infant exposed = (born between October 1, 1959 and September 30, 1961; gestation from January 1, 1959 to January 1, 1961)  Early childhood exposed = (born between October 1, 1956 and September 30, 1958)  Post- famine cohort/Unexposed = (born between October 1, 1962 and September 30, 1964) | Early childhood = born between 1965 and 1967  Fetal life and in infancy = born between 1968 and January 1970  Unexposed = born between 1971 and 1973  Transitional period = Feb–Dec 1970 | The vital status of the cohort members was ascertained through contacts with the participants, their relatives or neighbors. |
| Types of studies | cross-sectional | cohort study | cohort |
| Sources of information | CHNS 2009 | data from CHNS 2009 | Population registry |
| Country | China | Nigeria | Leningrad |
| Sample size | Unexposed = 623  Fetal exposed = 321  Early child exposed = 471  Total = 1415 | Early childhood = 388  Fetal-infant = 292  Unexposed = 486  Transitional period = 173  Total = 1338 | Born 1910–1915 = 91  Born 1916–1925 = 251  Born 1926–1932 = 130  Born 1933–1935 = 52  Born 1936–1940 = 81 |
| Sampling technique |  |  | Participants were selected randomly |
| Outcome studied | Hypertension | Blood pressure (BP), random plasma glucose (p-glucose) and anthropometrics, prevalence of hypertension, impaired glucose tolerance, diabetes, overweight | Blood pressure, hypertension and mortality from circulatory disease |
| Outcome measurement/definition | WHO |  | Systolic hypertension was defined as systolic blood pressure of 160+ mm Hg and diastolic hypertension as diastolic blood pressure of 95+ mm Hg. a copy of the official death certificate was obtained to register date of death and the underlying cause of death. These were coded according to ICD 8, as circulatory (ICD 390–459), ischaemic heart disease (IHD) (ICD 410–414), and cerebrovascular |
| Key findings/Proportions/Mean | **SBP (Mean ± SD)**  Unexposed = 121.93 (14.39)  Fetal exposed = 124.72 (16.17)  Early child exposed = 124.89 (15.31)  **Men, SBP (Mean ± SD)**  Unexposed = 124.13 (13.94)  Fetal exposed = 125.32 (15.27)  Early child exposed = 126.00 (14.16)  **Women, SBP (Mean ± SD)**  Unexposed = 119.74 (14.52)  Fetal exposed = 124.18 (16.96)  Early child exposed = 123.93 (16.21)  **DBP (Mean ± SD)**  Unexposed = 81.22 (10.27)  Fetal exposed = 81.74 (11.29)  Early child exposed = 82.46 (9.57)  **Men, DBP (Mean ± SD)**  Unexposed = 83.44 (10.09)  Fetal exposed = 82.52 (10.97)  Early child exposed = 83.75 (10.09)  **Women, DBP (Mean ± SD)**  Unexposed = 79.03 (9.99)  Fetal exposed = 81.04 (11.56)  Early child exposed = 81.33 (8.96)  **Hypertension (%)**  Unexposed = 22.6  Fetal exposed = 26.5  Early child exposed = 29.3  **Men, Hypertension (%)**  Unexposed = 27.7  Fetal exposed = 27.6  Early child exposed = 30.6  **Women, Hypertension (%)**  Unexposed = 17.6  Fetal exposed = 25.4  Early child exposed = 28.2 | **Systolic blood pressure, mm Hg**  Early childhood = 125 (17)  Fetal-infant = 129 (19)  Unexposed = 122(16)  Transitional period = 124 (19)  **Diastolic blood pressure, mm Hg**  Early childhood = 81 (11)  Fetal-infant = 84 (12)  Unexposed = 79 (11)  Transitional period = 81 (12)  **Random p-glucose, mmol/l**  Early childhood = 6.1 (1.6)  Fetal-infant = 6.4 (2.0)  Unexposed = 6.1 (1.8)  Transitional period = 6.3 (2.6)  **Height, cm**  Early childhood = 169 (8)  Fetal-infant = 169 (8)  Unexposed = 170 (8)  Transitional period = 169 (8)  **Waist circumference, cm**  Early childhood = 93 (11)  Fetal-infant = 94 (13)  Unexposed = 91 (11)  Transitional period = 92 (12)  **BMI, kg/m2**  Early childhood = 26.7 (4.7)  Fetal-infant = 27.5 (4.6)  Unexposed = 26.5 (4.4)  Transitional period = 26.6 (5.1) | **Height (cm)**  Born 1910–1915 = 153.9  Born 1916–1925 = 157.2  Born 1926–1932 = 158.3  Born 1933–1935 = 158.4  Born 1936–1940 = 158.0  **Systolic blood pressure (mm Hg)**  Born 1910–1915 = 168.5  Born 1916–1925 = 161.3  Born 1926–1932 = 148.2  Born 1933–1935 = 144.2  Born 1936–1940 = 130.1  **Diastolic blood pressure (mm Hg)**  Born 1910–1915 = 89.3  Born 1916–1925 = 90.7  Born 1926–1932 = 90.3  Born 1933–1935 = 88.9  Born 1936–1940 = 82.4  **Hypertension (‡160/95 mm Hg) %**  Born 1910–1915 = 55.0  Born 1916–1925 = 58.6  Born 1926–1932 = 36.9  Born 1933–1935 = 30.8  Born 1936–1940 = 19.8 |
| Effect Measures **(**95%CI OF AOR OR COR OR β-coefficients) | **SBP (Mean ± SD)**  Fetal exposed = 2.75 (0.83–4.66)  Early child exposed = 3.21 (1.54–4.88)  **Men, SBP (Mean ± SD)**  Fetal exposed = 1.19 (-1.51–3.89)  Early child exposed = 2.55 (0.23–4.88)  **Women, SBP (Mean ± SD)**  Fetal exposed = 4.24 (1.50–6.98)  Early child exposed = 3.86 (1.48–6.25)  **DBP (Mean ± SD)**  Fetal exposed = 0.48 (-0.85–1.80)  Early child exposed = 1.35 (0.23–2.47)  **Men, DBP (Mean ± SD)**  Fetal exposed = -1.00 (-2.91–0.92)  Early child exposed = 0.74 (-0.95–2.43)  **Women, DBP (Mean ± SD)**  Fetal exposed = 1.80 (-0.05–3.64)  Early child exposed = 1.88 (0.41–3.36)  **Hypertension (%)**  Fetal exposed = 1.24 (0.90–1.73)  Early child exposed = 1.48 (1.11–1.98)  **Men, Hypertension (%)**  Fetal exposed = 0.97 (0.61–1.54)  Early child exposed = 1.26 (0.85–1.86)  **Women, Hypertension (%)**  Fetal exposed = 1.62 (1.01–2.61)  Early child exposed = 1.78 (1.15–2.74) | **Adjusted OR (95% CI)**  **SBP>140**  Early childhood = 1.77 (1.17–2.68)  Fetal-infant = 2.87 (1.9–4.34)  **DBP <**  Early childhood = 1.30 (0.88–1.91)  Fetal-infant = 2.28 (1.56–3.34)  **sever HT (***SBP >160 and DBP>100 mmHg**)**  Early childhood = 1.42 (0.65–3.13)  Fetal-infant = 2.50 (1.19–5.26)  **IGT**  Early childhood = 1.13 (0.69–1.83)  Fetal-infant = 1.65 (1.02–2.69)  **Diabetes**  Early childhood = 1.81 (0.64–5.15)  Fetal-infant = 2.56 (0.92–7.17)  **Overweight**  Early childhood = 1.02 (0.77–1.34)  Fetal-infant=1.41(1.03–1.93) **Obesity**  Early childhood =1.20(0.87–1.67) Fetal-infant = 1.30 (0.92–1.85) | Women who were 6–8 year sold and men who were 9–15 years-old at the peak of starvation had higher systolic blood pressure compared to unexposed subjects born during the same period of birth (fully adjusted difference 8.8, 95% CI: 0.1–17.5 mm Hg in women and 2.9, 95% CI: 0.7– 5.0 mm Hg in men). Mean height of women who were exposed to siege as children appeared to be greater than that of unexposed women. Higher mortality from ischaemic heart disease and cerebrovascular disease was noted in men exposed at age 6–8 and 9–15, respectively. |
| Comment | Exposure to famine during the fetal-infant period or early childhood has deleterious effects on adult health, but the effects may be greater for women. Gender-specific intervention strategies for CVD may be warranted for populations exposed to under-nutrition during critical time periods of fetal development | Fetal and infant undernutrition is associated with significantly increased risk of hypertension and impaired glucose tolerance in 40-year-old Nigerians. Prevention of undernutrition during pregnancy and in infancy should therefore be given high priority in health, education, and economic agendas. | The experience of severe stress and starvation in childhood and puberty may have long-term effects on systolic blood pressure and circulatory disease in surviving men and women with potential gender differences in the effect of siege experienced at pre-pubertal age. |
| Adjustment for covariates | age, marital status, BMI, waist circumference, smoke and alcohol use | sex, smoking, drinking, BMI, education | age, smoking, alcohol and social characteristics |

| **Authors/year** | **(Li et al., 2011)** | **(Painter et al., 2006b)** | **(Shi et al., 2018)** |
| --- | --- | --- | --- |
| Participant characteristics | **Age in 2002 (years)**  Non-exposed = 38–39  Fetal exposed = 41–42  Early-child = 44–45  Mid-child = 46–47  Late-child = 48–49 | men and women, aged 58 years, born as term singletons in Amsterdam at about the time of the Dutch 1944–1945 famine | **Age in 2011 (years)**  Non-exposed cohort = 47.8 (0.6)  Fetal exposed cohort = 50.8 (0.6)  Early child exposed = 53.8 (0.6)  Mid childhood exposed = 55.8 (0.6)  Late childhood exposed = 57.7 (0.6) |
| Settings/context | women and men | women and men | surviving men and women |
| Famine year/duration |  |  |  |
| Assessment of famine Exposure | birthday and corresponding exposure period | official rations== less than 1000 calories | Date of birth |
| Exposure groups | **Birth day**  Non-exposed = 1 October 1962 - 30 September 1964  Fetal exposed = 1 October 1959 - 30 September 1961  Early-child = 1 October 1956 - 30 September 1958  Mid-child = 1 October 1954 - 30 September 1956  Late-child = 1 October 1952 - 30 September 1954 | Prenatally exposed = born between 7 January and 8 December 1945  Late gestation = (born between 7 January and 28 April 1945)  Mid-gestation = (born between 29 April and 18 August 1945)  Early gestation = (born between 19 August and 8 December 1945)  unexposed individuals = born before and individuals conceived after the famine | Nonexposed cohort (1 October 1962 to 30 September 1964)  Fetal exposed cohort (1 October 1959 to 30 September 1961)  Early child exposed (1 October 1956 to 30 September 1958)  Mid childhood exposed (1 October 1954 to 30 September 1956)  Late childhood exposed (1 October 1952 to 30 September 1954) |
| Types of studies | cross-sectional | Historical cohort study | cohort |
| Sources of information | 2002 CNNHS | Medical birth records provided information about the mother, the course of the pregnancy, size of the baby at birth and socio-economic status at birth | CHARLS study |
| Country | China | Dutch | China |
| Sample size | Non-exposed = 1954  Fetal exposed = 1005  Early-child = 1654  Mid-child =1588  Late-child = 1673  Total = 7874 | Born before = 228  Mid-gestation = 105  Late gestation = 125  Early gestation = 61  Conceived after =202  Total = 721 | Nonexposed cohort = 1394  Fetal exposed cohort = 762  Early child exposed = 1149  Mid childhood exposed = 1217  Late childhood exposed = 1250  Total = 5772 |
| Sampling technique |  |  |  |
| Outcome studied | Hypertension | Blood pressure response to psychological stressors  BMI | Hypertension and cardiovascular disease  Multimorbidity |
| Outcome measurement/definition | 1999 WHO/International Society of Hypertension guidelines |  | self-reported cardiovascular disease. Multimorbidity was defined as having at least three of the following seven conditions: diabetes, hypertension, central obesity, dyslipidaemia, CKD, anemia, and asthma. |
| Key findings/Proportions/Mean | **Height (cm)**  Non-exposed = 160.9 (0.2)  Fetal exposed = 160.0 (0.4)  Early-child = 159.4 (0.2)  Mid-child = 159.4 (0.4)  Late-child = 159.3 (0.2)  **BMI (kg/m2)**  Non-exposed = 22.9 (0.1)  Fetal exposed = 23.2 (0.1)  Early-child = 23.2 (0.2)  Mid-child = 23.1 (0.2)  Late-child = 23.3 (0.1)  **Hypertension (%)**  Non-exposed = 12.8  Fetal exposed = 19.3  Early-child = 23.3  Mid-child = 22.5  Late-child = 17.4 | **SBP (mmHg)**  Born before = 138  Mid-gestation = 137  Late gestation = 136  Early gestation = 135  Conceived after = 136  **DBP (mmHg)**  Born before = 81  Mid-gestation = 81  Late gestation = 80  Early gestation = 82  Conceived after = 82  **BMI (kg/m2)**  Born before = 28.4  Mid-gestation = 28.1  Late gestation = 27.9  Early gestation = 27.9  Conceived after = 28.8 | **SBP, mean (SD)**  Non-exposed cohort = 124.5 (17.9)  Fetal exposed cohort = 126.3 (19.7)  Early child exposed = 128.1 (20.5)  Mid childhood exposed = 127.1 (19.4)  Late childhood exposed = 130.7 (21.3)  **DBP, mean (SD)**  Non-exposed cohort = 76.6 (12.4)  Fetal exposed cohort = 77.0 (12.8)  Early child exposed = 76.8 (13.0)  Mid childhood exposed = 76.4 (12.3)  Late childhood exposed = 77.1 (12.4)  **BMI (kg/m2), mean (SD)**  Non-exposed cohort = 24.2 (3.6)  Fetal exposed cohort = 24.3 (4.4)  Early child exposed = 23.9 (3.9)  Mid childhood exposed = 23.3 (3.6)  Late childhood exposed = 23.7 (3.8)  **Hypertension (%)**  Non-exposed cohort = 20.8  Fetal exposed cohort = 22.4  Early child exposed = 25.9  Mid childhood exposed = 25.1  Late childhood exposed = 28.6  **CVD (%)**  Non-exposed cohort = 8.6  Fetal exposed cohort = 9.4  Early child exposed = 10.4  Mid childhood exposed = 12.9  Late childhood exposed = 12.0  **CKD (%)**  Non-exposed cohort = 8.9  Fetal exposed cohort = 7.0  Early child exposed = 7.8  Mid childhood exposed = 9.7  Late childhood exposed = 7.5  **Anemia (%)**  Non-exposed cohort = 12.1  Fetal exposed cohort = 8.1  Early child exposed = 10.3  Mid childhood exposed = 10.4  Late childhood exposed = 9.4  **Central obesity (%)**  Non-exposed cohort = 57.0  Fetal exposed cohort = 54.7  Early child exposed = 54.8  Mid childhood exposed = 52.1  Late childhood exposed = 53.4  **Dyslipidemia (%)**  Non-exposed cohort = 67.8  Fetal exposed cohort = 73.4  Early child exposed = 73.0  Mid childhood exposed = 67.7  Late childhood exposed = 71.6  **Stroke (%)**  Non-exposed cohort = 1.0  Fetal exposed cohort = 1.1  Early child exposed = 1.1  Mid childhood exposed = 1.6  Late childhood exposed = 1.8  **Asthma (%)**  Non-exposed cohort = 1.9  Fetal exposed cohort = 1.6  Early child exposed = 2.1  Mid childhood exposed = 2.3  Late childhood exposed = 3.5 |
| Effect Measures **(**95%CI OF AOR OR COR OR β-coefficients) | **Hypertension, OR (95% CI)**  Fetal exposed = 1.88 (1.00–3.53)  Early-child = 1.74 (1.04–2.89)  Mid-child = 2.22 (1.33–3.71)  Late-child = 1.43 (0.84–2.44) |  | **Hypertension, OR (95% CI)**  Non-exposed cohort = 1.40 (0.82–2.38)  Fetal exposed cohort = 3.35 (1.54–7.27)  Early child exposed = 2.48 (1.49–4.11)  Mid childhood exposed = 2.35 (1.44–3.83)  Late childhood exposed = 1.69 (1.06–2.72)  **Multimorbidity, OR (95% CI)**  Non-exposed cohort = 1.33 (0.76–2.33)  Fetal exposed cohort = 2.55 (1.13–5.72)  Early child exposed = 2.54 (1.50–4.31)  Mid childhood exposed = 1.61 (0.97–2.69)  Late childhood exposed = 1.24 (0.74–2.07) |
| Comment | fetal famine exposure is associated with higher blood pressure and an increased risk of hypertension in adulthood. These associations are stronger in participants who have a western dietary pattern or who are overweight as adults | We found a greater blood pressure increase during stress among individuals exposed to famine in early gestation. Increased stress responsiveness may underlie the known association between coronary heart disease and exposure to famine in early gestation | Early life exposure to the Chinese famine exacerbated the association between hypertension and CVD, especially among women and those living in urban areas or those with central obesity |
| Adjustment for covariates | sex, family income, educational level, current smoking, alcohol use, physical activity level, dietary intake of salt, calcium, fat, red meat, fruit and vegetable, BMI and family history of hypertension. | sex, smoking, drinking, education | Age, sex, smoking, alcohol drinking, education, urban/rural, physical activity, BMI, famine severity, CRP. |

| **Authors/year** | **(Stanner and Yudkin, 2001)** | **(Stein et al., 2006, Vågerö et al., 2013)** | **(Vågerö et al., 2013)** |
| --- | --- | --- | --- |
| Participant characteristics | **Age (years**)  Intrauterine exposed = 52.3  Infant exposed = 53.1  Unexposed = 52.8 | **Age (y)**  Famine exposed = 58.7 (0.42)  Hospital controls = 58.6 (1.56)  Siblings = 57.2 (6.34)  Mean age 59 y | **Age at peak starvation**  1-5  6-8  9-11  12-15  16-25 |
| Settings/context | women and men | women and men | surviving men and women |
| Famine year/duration | 1941–4 |  | 1941–1944 |
| Assessment of famine Exposure | birthdates and place of participants | official ration of <900 kcal/week | **Age at peak starvation** |
| Exposure groups | intrauterine starvation (during the Siege of Leningrad, 1941–4)  Infant exposed = born in Leningrad before the siege  Unexposed = born concurrently with these two groups but outside the area of the siege | Exposed = Born before or during gestation  Unexposed = born before or conceived after the famine OR same-sex siblings of subjects | The vital status of the cohort members was ascertained through contacts with the participants, their relatives or neighbors. |
| Types of studies | cross-sectional | cohort study | cohort |
| Sources of information |  | live singleton births at three institutions in famine-exposed cities | Population registry |
| Country | Leningrad Siege | Dutch | Leningrad |
| Sample size | Intrauterine exposed = 169  Infant exposed = 192  Unexposed = 188  Total = 549 | Famine exposed = 359  Hospital controls = 299  Siblings = 313  Total = 971 | men (n=3899) and women (n=1428)  Total = 5328 |
| Sampling technique |  |  | From 1975 to 1982 men and women living in Leningrad (now St. Petersburg) were randomly sampled and invited to examine their health and cardiovascular functioning |
| Outcome studied | Hypertension | blood pressure at age 59 y | Blood pressure, hypertension and mortality from circulatory disease |
| Outcome measurement/definition | Blood pressure (BP) measurements, self-reported previous diagnosis of hypertension and current anti-hypertension drug use | European Society of Hypertension Working Group on BP Monitoring |  |
| Key findings/Proportions/Mean | **Height (m), Male**  Intrauterine exposed = 1.72 (1.70-1.74)  Infant exposed = 1.74 (1.72-1.76)  Unexposed = 1.73 (1.71-1.75)  **Height (m), Female**  Intrauterine exposed = 1.58 (1.56-1.60)  Infant exposed = 1.59 (1.57-1.61)  Unexposed = 1.60 (1.56-1.64)  **BMI (kg/m2, Male**  Intrauterine exposed = 24.6 (23.6-25.6)  Infant exposed = 25.4 (24.2-26.6)  Unexposed = 25.2 (24.1-26.3)  **BMI (kg/m2, Female**  Intrauterine exposed = 26.9 (26.1-27.7)  Infant exposed = 27.0 (26.2-27.8)  Unexposed = 26.7 (25.9-27.5)  **WHR, Male**  Intrauterine exposed = 0.86 (0.84-0.88)  Infant exposed = 0.88 (0.84-0.92)  Unexposed = 0.87 (0.85-0.89)  **WHR, Female**  Intrauterine exposed = 0.79 (0.77-0.81)  Infant exposed = 0.78 (0.76-0.80)  Unexposed = 0.79 (0.75-0.83)  **Fasting Glucose (mmol/l)**  Intrauterine exposed = 0.79 (0.77-0.81)  Infant exposed = 5.3 (5.1-5.5)  Unexposed = 5.3 (5.1-5.5)  **SBP**  Intrauterine exposed = 131.6 (127.9-135.3)  Infant exposed = 133.1 (129.9-136.3)  Unexposed = 128.5 (125.3-131.7)  **DBP**  Intrauterine exposed = 80.9 (78.7-83.1)  Infant exposed = 82.2 (80.0-84.3)  Unexposed = 77.3 (75.4-79.2) | **Height (cm)**  Famine exposed = 170.8 (8.9)  Hospital controls = 171.5 (9.0)  Siblings = 171.8 (8.9)  **Waist circumference (cm)**  Famine exposed = 99.9 (11.5)  Hospital controls = 97.4 (11.4)  Siblings = 96.4 (11.2)  **SBP (mmHg)**  Famine exposed = 142.7 (20.6)  Hospital controls = 139.7 (20.5)  Siblings = 138.2 (19.6)  **DBP (mmHg)**  Famine exposed = 86.7 (11.2)  Hospital controls = 85.9 (11.0)  Siblings = 84.7 (10.7)  **hypertension (%)**  Famine exposed = 67.4  Hospital controls = 60.9  Siblings = 56.2 |  |
| Effect Measures **(**95%CI OF AOR OR COR OR β-coefficients) |  | **Hypertension (95% CI)**  Gestational weeks 1–10 = 1.14 (0.62, 2.11)  Gestational weeks 11–20= 0.98 (0.59, 1.65)  Gestational weeks 21–30= 1.23 (0.74, 1.05)  Gestational weeks 31–delivery = 1.42 (0.86, 2.35)  **SBP (95% CI)**  Gestational weeks 1–10 = 1.20 (-3.28, 5.69)  Gestational weeks 11–20= -1.18 (-4.92, 2.55)  Gestational weeks 21–30= 1.33 (-2.24, 4.90)  Gestational weeks 31–delivery = 2.02 (-1.53, 5.57)  **DBP (95% CI)**  Gestational weeks 1–10 = 1.10 (-1.36, 3.57)  Gestational weeks 11–20= -1.26 (-3.32, 0.80)  Gestational weeks 21–30= 1.19 (-0.78, 3.15)  Gestational weeks 31–delivery = 0.71 (-1.24, 2.66) | **SBP (95 CI), Men**  6-8 = -2.26 (-5.5; 0.9)  9-11 =1.83 (-1.1;4.8)  12-15 =5.58 (2.8;8.3)  16-25 = 1.38 (-0.9; 3.6)  **DBP (95 CI), Men**  6-8 = -0.60 (-2.4;1.2)  9-11 =0.99 (-0.6; 2.6)  12-15 =1.57 (0.0; 3.1)  16-25 = 1.01 (-0.3; 2.3)  **SBP (95 CI), Women**  6-8 = 4.54 (-3.9; 12.9)  9-11 = -1.81 (-9.3; 5.7)  12-15 = 5.77 (-1.9; 13.4)  16-25 = -0.59 (-5.8; 4.6)  **DBP (95 CI), Women**  6-8 = 0.61 (-3.4;4.6)  9-11 = 0.02 (-3.5;3.6)  12-15 = 1.39 (-2.2;5.0)  16-25 = -0.26 (-2.7; 2.2) |
| Comment | This study did not find an association between intrauterine starvation and glucose intolerance, dyslipidaemia, hypertension or cardiovascular disease in adult life. The intrauterine exposed group had evidence of endothelial dysfunction by higher concentrations of von Willebrand factor and a stronger interaction between adult obesity and blood pressure | Exposure to famine during gestation may predispose to the development of hypertension in middle age. | The siege of Leningrad, particularly when experienced in puberty, has had long-term effects on blood pressure both in men and women. We also found a raised IHD and stroke risk among those men. This was partly mediated via blood pressure but not by any other measured biological, behavioral, or social factors. Girls experiencing the siege around puberty suffered an elevated risk of dying from breast cancer later in life. The fact that the effect of siege exposure is modified by the age at exposure is highly interesting from a scientific point of view. It may suggest that a reprogramming of physiological systems can occur at specific age windows in response to starvation and/or war trauma. |
| Adjustment for covariates | age, marital status, BMI, waist circumference, smoke and alcohol use | Age-, sex, smoking, alcohol intake, height, waist circumference | age, smoking, alcohol and social characteristics |

| **Authors/year** | **(Wang et al., 2012)** | **(Wang et al., 2016d)** | **(Yu et al., 2017)** |
| --- | --- | --- | --- |
| Participant characteristics | **Age in 2010 (years)**  Infancy (,2 years postnatal) = 51–53  Both fetal and infancy = 49–51  Fetal only = 48–49  1st,2nd and 3rd trimester = 49  1st and 2nd trimester = 48  1st trimester = 48  No exposure = 46–48 | **Age mean(SD) years**  Non-exposed cohort = 46.78(0.41)  Fetal-exposed cohort = 50.41(0.62)  Infant-exposed cohort = 52.54(0.50)  Preschool-exposed cohort = 54.30(0.70) | **Age in 2013**  Late-child = 60.1 (0.60)  Mid-child = 58.0 (0.59)  Early-child = 56.1 (0.55)  Fetal exposed = 53.0 (0.59)  Non exposed = 50.3 (0.58) |
| Settings/context | women and men | women and men | surviving men and women |
| Famine year/duration |  |  |  |
| Assessment of famine Exposure | Date of birth | participants’ birth dates | Birth Date |
| Exposure groups | Infancy (,2 years postnatal) = 01/01/1957-12/31/1958  Both fetal and infancy = 01/01/1959-09/30/1961  Fetal only = 10/01/1961-06/30/1962  1st,2nd and 3rd trimester = 10/01/1961-12/31/1961  1st and 2nd trimester =01/01/1962-03/31/1962  1st trimester = 04/01/1962-06/30/1962  No exposure = 07/01/1962-12/31/1964 | Non-exposed cohort = 10/1/1962-9/30/1964  Fetal-exposed cohort = 10/1/1959-9/30/1961  Infant-exposed cohort = 1/1/1958-12/31/1958  Preschool-exposed cohort = 1/1/1956-12/31/1957 | Late-child = 1952–1954  Mid-child = 1954–1956  Early-child = 1956–1958  Fetal exposed =1959–1961  Non exposed = 1962–1964 |
| Types of studies | Retrospective cohort | cohort study | cohort |
| Sources of information |  | live singleton births at three institutions in famine-exposed cities | Population registry |
| Country | China | China | China |
| Sample size | Infancy = 3126  Fetal and infancy = 2911  Fetal only = 1156  No exposure = 4872  Total = 12,065 | Non-exposed cohort = 572  Fetal-exposed cohort = 599  Infant-exposed cohort = 338  Preschool-exposed cohort = 457  Total = 1,966 | Late-child = 2248  Mid-child = 1941  Early-child = 2115  Fetal exposed = 1394  Non exposed = 1044  Total = 8,742 |
| Sampling technique |  |  |  |
| Outcome studied | Hypertension | Hypertension | Hypertension |
| Outcome measurement/definition | WHO | After at least a 10-min rest, blood pressure (BP) was measured on the participant’s left arm in seated position to the nearest mmHg | WHO |
| Key findings/Proportions/Mean | **Hypertension**  Infancy = 19.4%  Fetal and infancy = 13.9%  Fetal only = 12.5%  No exposure = 11.1%  **SBP*, mmHg**  Infancy = 127.2±0.4  Fetal and infancy = 125.7±0.4  Fetal only = 124.9±0.5  No exposure = 124.2±0.4  **DBP*, mmHg**  Infancy = 81.8±0.3  Fetal and infancy = 80.8±0.3  Fetal only = 80.9±0.3  No exposure = 80.2±0.3  **BMI, kg/m2, males**  Infancy = 23.5±0.10  Fetal and infancy = 23.2±0.10  Fetal only = 23.3±0.14  No exposure = 23.3±0.09  **BMI, kg/m2, females**  Infancy = 22.8±60.28  Fetal and infancy = 22.7±0.28  Fetal only = 22.6±0.30  No exposure = 22.6±0.28  **Obesity (>28 kg/m2), Male**  Infancy = 5.9%  Fetal and infancy = 4.2%  Fetal only = 5.8%  No exposure = 5.9%  **Obesity (>28 kg/m2), females**  Infancy = 6.7%  Fetal and infancy = 6.4%  Fetal only = 5.6%  No exposure = 5.3%  **Height, cm, males**  Infancy = 166.8±60.2  Fetal and infancy = 167.2±0.2  Fetal only = 167.5±0.3  No exposure = 168.0±0.2  **Height, cm, females**  Infancy = 157.5±0.5  Fetal and infancy = 157.9±0.5  Fetal only = 158.3±0.5  No exposure = 158.7±0.5  **Short (< 10th percentile),** male  Infancy = 13.4%  Fetal and infancy = 9.5%  Fetal only = 11.0%  No exposure = 9.0%  **Short (< 10th percentile),** female  Infancy = 13.4%  Fetal and infancy = 10.5%  Fetal only = 8.2%  No exposure = 8.3% | **Hypertension**  Non-exposed cohort = 18.9%  Fetal-exposed cohort = 20.7%  Infant-exposed cohort = 28.7%  Preschool-exposed cohort = 23.4%  **SBP mean(SD) mmHg**  Non-exposed cohort = 125.19(17.36)  Fetal-exposed cohort = 127.90(20.18)  Infant-exposed cohort = 131.45(19.41)  Preschool-exposed cohort = 129.01(20.00)  **DBP mean(SD) mmHg**  Non-exposed cohort = 77.86(12.81)  Fetal-exposed cohort = 77.95(12.70)  Infant-exposed cohort = 79.06(12.89)  Preschool-exposed cohort = 77.47(12.55)  **BMI mean(SD) kg/m^2^**  Non-exposed cohort = 24.75(3.85)  Fetal-exposed cohort = 24.61(4.14)  Infant-exposed cohort = 24.00(3.46)  Preschool-exposed cohort = 23.78(3.65) | **Hypertension (%)**  Late-child = 54.4  Mid-child = 47.4  Early-child = 43.9  Fetal exposed = 38.0  Non exposed = 34.0  **SBP (mmHg)**  Late-child = 136.5 (21.70)  Mid-child = 133.3 (21.27)  Early-child = 130.4 (20.62)  Fetal exposed = 128.8 (21.01)  Non exposed = 126.6 (20.40)  **DBP (mmHg)**  Late-child = 80.4 (12.23)  Mid-child = 79.5 (12.24)  Early-child = 78.5 (12.21)  Fetal exposed = 78.2 (12.26)  Non exposed = 78.1 (12.54)  **Overweight/obesity (%)**  Late-child = 50.2**%**  Mid-child = 47.3**%**  Early-child = 46.3**%**  Fetal exposed = 44.6**%**  Non exposed = 42.7**%**  **Central obesity**  Late-child = 50.5**%**  Mid-child = 50.8 **%**  Early-child = 49.0**%**  Fetal exposed = 45.2 **%**  Non exposed = 45.2**%**  **Height (cm)**  Late-child = 159.5 (7.51)  Mid-child = 159.6 (7.34)  Early-child = 159.1 (6.90)  Fetal exposed = 157.9 (6.29)  Non exposed = 159.4 (6.30) |
| Effect Measures **(**95%CI OF AOR OR COR OR β-coefficients) | **SBP, mmHg, Difference**  Infancy = 3.0 (2.4, 3.6)  Fetal and infancy = 1.5 (0.9, 2.2)  Fetal only = 0.7 (-0.2, 1.6)  **DBP, mmHg, Difference**  Infancy = 1.6 (1.2, 2.0)  Fetal and infancy = 0.6 (0.2, 1.0)  Fetal only = 0.7 (0.2, 1.3)  **BMI, kg/m2, males, Difference**  Infancy = 0.12 (-0.08, 0.31)  Fetal and infancy = -0.18 (-0.38, 0.02)  Fetal only = -0.03 (-0.30, 0.25)  **BMI, kg/m2, females, Difference**  Infancy = 0.17 (-0.03, 0.38)  Fetal and infancy = 0.12 (-0.09, 0.33)  Fetal only = -0.02 (-0.23, 0.28)  **Height, cm, males, Difference**  Infancy = -1.1 (-1.5, -0.8)  Fetal and infancy = -0.8 (-1.1, -0.4)  Fetal only = -0.5 (-1.0, 0.05)  **Height, cm, females, Difference**  Infancy = -1.2 (-1.5, -0.9)  Fetal and infancy = -0.8 (-1.2, -0.5)  Fetal only = -0.4 (-0.9, 0.1)  **Adjusted OR, HTN**  Infancy = 1.83 (1.61, 2.08)  Fetal and infancy = 1.31 (1.14, 1.51)  Fetal only = 1.16 (0.95, 1.42  **Obesity (>28 kg/m2), Male**  Infancy = 1.02 (0.77, 1.35)  Fetal and infancy = 0.72 (0.53, 0.98)  Fetal only = 1.00 (0.68, 1.47)  **Obesity (>28 kg/m2), females**  Infancy = 1.21 (0.93, 1.58)  Fetal and infancy = 1.24 (0.94, 1.63)  Fetal only = 1.08 (0.72, 1.61)  **Short (< 10th percentile),** male  Infancy = 1.57 (1.28, 1.93)  Fetal and infancy = 1.12 (0.89, 1.40)  Fetal only = 1.31 (0.97, 1.76)  **Short (< 10th percentile),** female  Infancy = 1.66 (1.35, 2.04)  Fetal and infancy = 1.27 (1.02, 1.59)  Fetal only = 1.05 (0.75, 1.46)  No exposure = 8.3% | **OR (95 % CI)**  Fetal-exposed cohort = 0.92(0.45–1.88)  Infant-exposed cohort = 1.66(1.04–2.66)  Preschool-exposed cohort = 1.33(0.83–2.14) | **OR (95% CI), hypertension**  Late-child = 2.11 (1.75–2.55)  Mid-child = 1.67 (1.38–2.02)  Early-child = 1.44 (1.20–1.73)  Fetal exposed = 1.24 (1.01–1.51)  **Male**  Late-child = 2.07 (1.69–2.53)  Mid-child = 1.62 (1.32–1.98)  Early-child = 1.42 (1.17–1.73)  Fetal exposed = 1.24 (1.01–1.52)  **Female**  Late-child = 2.47 (1.40–4.34)  Mid-child = 1.98 (1.11–3.53)  Early-child = 1.62 (0.90–2.91)  Fetal exposed = 1.13 (0.52–2.47)  **BMI<24**  Late-child = 1.94 (1.50–2.51)  Mid-child = 1.49 (1.16–1.93)  Early-child = 1.16 (0.90–1.50)  Fetal exposed = 0.99 (0.75–1.30)  **BMI>24**  Late-child = 2.30 (1.75–3.02)  Mid-child = 1.86 (1.41–2.44)  Early-child = 1.80 (1.38–2.35)  Fetal exposed = 1.58 (1.18–2.10)  **Central obesity, Normal**  Late-child = 1.98 (1.52–2.58)  Mid-child = 1.69 (1.30–2.21)  Early-child = 1.25 (0.97–1.62)  Fetal exposed = 1.07 (0.81–1.41)  **Central obesity, increased WC**  Late-child = 2.22 (1.71–2.90)  Mid-child = 1.63 (1.25–2.12)  Early-child = 1.64 (1.26–2.13)  Fetal exposed = 1.45 (1.09–1.92)  **Famine, less**  Late-child = 1.89 (1.29–2.76)  Mid-child = 1.18 (0.81–1.71)  Early-child = 1.19 (0.84–1.69)  Fetal exposed = 1.03 (0.71–1.49)  **Famine, severe**  Late-child = 2.20 (1.77–2.74)  Mid-child = 1.90 (1.52–2.36)  Early-child = 1.56 (1.25–1.94)  Fetal exposed = 1.31 (1.04–1.66) |
| Comment | Exposure to famine during infancy increased the risk of short stature. Early life exposure to famine did not increase the risk of obesity. Exposure to the Chinese Great Famine during the first trimester of pregnancy only, or during infancy only, or during both fetal development and infancy increased the risk of hypertension in adulthood, suggesting an important role of changes in exposure to famine during fetal development and from prenatal to early postnatal life in developmental ‘‘programming’’ cardiovascular disease risk. | Infanthood exposed to famine might increase the risk of hypertension in adulthood, and a postnatal ‘rich’ nutrient environment further increased the risk. | Exposure to the famine in early life increases the risk of hypertension prevalence in adulthood |
| Adjustment for covariates | Adjusted for socio-demographic and lifestyle characteristics (see Table 2); for blood pressure and hypertension, further adjusted for short stature and BMI; subjects with  anti-hypertensive treatment were included in the models for hypertension, but excluded in the models for SBP and DBP | Age, BMI, gender, smoking and drinking | Sex, education, smoking status, drinking status, physical activity, family history of hypertension, fruit intake, and vegetable intake, BMI, central obesity, famine severity. |

| **Authors/year** | **(Zhao et al., 2019)** | **(Liu et al., 2017b)** | **(Finer et al., 2016)** |
| --- | --- | --- | --- |
| Participant characteristics | Age, year (All subjects) = 36.94 ± 8.17 | **Age (Mean/SD)**  Childhood exposure = 49.69 (0.79)  Fetal exposure = 47.28 (0.45)  Non-exposure = 43.44 (0.62) | **Age (years)**  In utero exposed = 30 (0.3)  Postnatal exposed = 31 (0.4)  Unexposed = 28 (0.3) |
| Settings/context | women and men | women and men | women and men |
| Famine year/duration |  |  | July 1974 and June 1975 |
| Assessment of famine Exposure | Date of birth | participants’ birth dates | birth records |
| Exposure groups | No exposure = born between 1962 and 1965  Fetal exposure = born between 1959 and 1961  early childhood exposure = born between 1956 and 1958  mid-childhood exposure = born between 1953 and 1955  late childhood exposure = born between 1949 and 1952 | Childhood exposure Fetal exposure  Non-exposure | Postnatal famine exposure (born 1–2 years before the start of famine)  In utero exposure (including at least 7 months of famine exposure during gestation)  Unexposed (conceived 6 months to 2 years after famine). |
| Types of studies | A 22-year cohort study | cohort study | Cross-sectional |
| Sources of information | CHNS: ongoing, open-cohort project | live singleton births at three institutions in famine-exposed cities | birth records and using the famine start and end dates |
| Country | China | China | Bangladesh |
| Sample size | No exposure = 2088  Fetal exposure = 880  early childhood exposure = 1214  mid-childhood exposure = 1287  late childhood exposure = 1445  Total = 6,914 | Childhood exposure = 455  Fetal exposure = 299  Non-exposure = 470  Total = | In utero exposed = 68  Postnatal exposed = 81  Unexposed = 70  Total = 219 |
| Sampling technique | multistage random cluster |  | Randomized subsample |
| Outcome studied | Hypertension | Hypertension | BMI (kg/m2)  Waist circumference  Impaired fasting glucose  impaired glucose tolerance  type 2 diabetes |
| Outcome measurement/definition | After at least a 20-minutes rest, blood pressure (BP) was measured on the participant’s right arm in seated position to the nearest mmHg | After at least a 10-min rest, blood pressure (BP) was measured on the participant’s left arm in seated position to the nearest mmHg |  |
| Key findings/Proportions/Mean |  | **Hypertension (%), Men**  Childhood exposure = 56.70  Fetal exposure = 55.85  Non-exposure = 55.74 | **BMI (kg/m2)**  In utero exposed = 19.4 (2.9)  Postnatal exposed = 20.3 (3.1)  Unexposed = 20.0 (2.6)  **BMI category, Underweight (%)**  In utero exposed = 49  Postnatal exposed = 32  Unexposed = 30  **BMI category, Normal weight n (%)**  In utero exposed = 38  Postnatal exposed = 42  Unexposed = 56  **BMI category, Normal weight (%)**  In utero exposed = 38  Postnatal exposed = 42  Unexposed = 56  **BMI category, Overweight (%)**  In utero exposed = 13  Postnatal exposed = 26  Unexposed = 14  **Waist circumference, Normal**  In utero exposed = 63 (93)  Postnatal exposed = 71 (88)  Unexposed = 65 (93)  **Waist circumference, High (M≥90 cm; F≥80 cm)**  In utero exposed = 7  Postnatal exposed = 12  Unexposed = 7  **0 min glucose (mmol/l)** Mean(SD)  In utero exposed = 4.8 (0.5)  Postnatal exposed = 4.8 (0.5)  Unexposed = 4.7 (0.6)  **120 min glucose (mmol/l),** Mean(SD)  In utero exposed = 5.8 (1.6)  Postnatal exposed = 5.2 (1.4)  Unexposed = 5.6 (2.0)  **IFG/IGT/T2D (%)**  In utero exposed = 13  Postnatal exposed = 9  Unexposed = 14 |
| Effect Measures **(**95%CI OF AOR OR COR OR β-coefficients) | **HR 95% CI**  Fetal exposure = 0.791 (0.680-0.920)  early childhood exposure = 0.760 (0.661-0.873)  mid-childhood exposure = 0.710 (0.613-0.822)  late childhood exposure = 0.613 (0.523-0.718)  **HR 95% CI, Males**  Fetal exposure = 0.728 (0.591-0.896)  early childhood exposure = 0.652 (0.537-0.792)  mid-childhood exposure = 0.620 (0.505-0.760)  late childhood exposure = 0.522 (0.419-0.650)  **HR 95% CI, Females**  Fetal exposure = 0.855 (0.676-1.081)  early childhood exposure = 0.910 (0.737-1.125)  mid-childhood exposure = 0.842 (0.676-1.050)  late childhood exposure = 0.733 (0.579-0.929) | **AOR (95% CI)**  Childhood exposure = 1.24 (0.80-1.91)  Fetal and infant exposure = 1.79 (1.13-2.84)  **Men**  Childhood exposure = 1.33 (0.80-2.22)  Fetal and infant exposure = 1.67 (0.95-2.92)  **Women**  Childhood exposure = 1.08 (0.45-2.59)  Fetal and infant exposure = 2.34 (1.01-5.42) |  |
| Comment | Exposure to the Chinese famine decreased the incidence of hypertension, especially in males and in the rural areas. Furthermore, the exposure postponed the age at the onset of hypertension. | Fetal exposure to the Chinese famine may be associated with an increased risk of hypertension in adulthood in women |  |
| Adjustment for covariates | age, sex, BMI, smoking, drinking, physical activity, ethnicity, income, famine severity, region, and death were adjusted in total population; and age, BMI, smoking, drinking, physical activity, ethnicity, income, famine severity, region, and death were adjusted in male and female population | gender, education, smoking, alcohol drinking, physical activity, sleep status, breast feeding, diabetes, BMI |  |

| **Authors/year** | **(Du et al., 2020)** | **(Zhou et al., 2019)** | **(Woo et al., 2010)** |
| --- | --- | --- | --- |
| Participant characteristics | Age, y  Non-exposed = 44.78 ± 2.82  Fetal- Exposed = 50.67±1.42  Childhood- Exposed = 57.56±2.92  Adolescent- Exposed = 66.24±2.40 | Adults aged 45–60 years | men and women aged ≥ 65 years  The mean (SD) age of exposure to famine was 12 (6) and 12 (7) years for men and women respectively |
| Settings/context | women and men | women and men | Community Hong Kong. |
| Famine year/duration |  |  | 1959 - 1961/ 3 year |
| Assessment of famine Exposure | Date of birth | participants’ birth dates | caloric restriction |
| Exposure groups | Non-exposed (1963–1974)  Fetal- Exposed Cohorts (1959–1962)  Childhood- Exposed Cohorts (1949–1958)  Adolescent- Exposed Cohorts = (1941–1948) | Non-exposed = 381  Late-childhood exposed = 141  Mid-childhood exposure= 173  Early-childhood exposure= 160  Fetal exposure group= 84 | Participants were classified as having exposure to famine if  they had experienced caloric restriction for a continuous period of at least one year during their childhood. The question asked was whether during their childhood, they had a prolonged period lasting at least a year when they did not have enough food to eat |
| Types of studies | A Longitudinal) Study | Cross-sectional | Cross sectional cohort survey |
| Sources of information | Risk Evaluation of Cancers in Chinese Diabetic Individuals | live singleton births at three institutions in famine-exposed cities | birth records and using the famine start and end dates |
| Country | China | China | Hong Kong |
| Sample size | Non-exposed =54 525  Fetal- Exposed =29 387  Childhood- Exposed =102 370  Adolescent- Exposed =47 937  Total = 234,219 | Childhood exposure = 455  Fetal exposure = 299  Non-exposure = 470 | Did not experience =1510  Experienced famine =2222  Total = 3732 |
| Sampling technique | multistage random cluster |  | Randomized subsample |
| Outcome studied | CVD, myocardial infarction, stroke, and coronary heart diseases | Dietary Pattern and Chronic Diseases | Body mass index, body composition |
| Outcome measurement/definition | self- reported | After at least a 10-min rest, blood pressure (BP) was measured on the participant’s left arm in seated position to the nearest mmHg | DEXA, grip strength, walking speed and stride length, blood pressure, and ankle-brachial index |
| Key findings/Proportions/Mean | **BMI, kg/m2**  Non-exposed = 24.41±3.61  Fetal- Exposed = 24.67±3.49  Childhood- Exposed = 24.74±3.58  Adolescent- Exposed = 24.82±3.62  **SBP, mm Hg**  Non-exposed = 124±18  Fetal- Exposed = 128±19  Childhood- Exposed = 133±20  Adolescent- Exposed = 140±21  **DBP, mm Hg**  Non-exposed = 77±12  Fetal- Exposed = 79±11  Childhood- Exposed = 79±11  Adolescent- Exposed = 78±11  **TG, mmol/L**  Non-exposed = 1.17 (0.83–1.75)  Fetal- Exposed = 1.28 (0.91–1.89)  Childhood- Exposed = 1.36 (0.97–1.96)  Adolescent- Exposed = 1.35 (0.98–1.92)  **TG, mmol/L**  Non-exposed = 1.32±0.35  Fetal- Exposed = 1.35±0.36  Childhood- Exposed = 1.34±0.36  Adolescent- Exposed = 1.32±0.36  **LDL- C, mmol/L**  Non-exposed = 2.64±0.80  Fetal- Exposed = 2.88±0.85  Childhood- Exposed = 2.95±0.87  Adolescent- Exposed = 2.91±0.88  **TC, mmol/L**  Non-exposed = 4.64±1.07  Fetal- Exposed = 4.96±1.11  Childhood- Exposed = 5.06±1.12  Adolescent- Exposed = 5.00±1.13  **CVD (%)**  Non-exposed = 1.09  Fetal- Exposed = 2.55  Childhood- Exposed = 5.70  Adolescent- Exposed = 11.23  **MI (%)**  Non-exposed = 0.10  Fetal- Exposed = 0.21  Childhood- Exposed = 0.43  Adolescent- Exposed = 0.68  **Stroke (%)**  Non-exposed = 0.27  Fetal- Exposed = 0.58  Childhood- Exposed = 1.30  Adolescent- Exposed = 2.63  **CHD, (%)**  Non-exposed = 0.77  Fetal- Exposed = 1.91  Childhood- Exposed = 4.35  Adolescent- Exposed = 8.70 | **Healthy’ dietary pattern**  Late-childhood exposed = 0·098  Mid-childhood exposure= -0·188  Early-childhood exposure= -0·005  Fetal exposure group= 0·103  **High-fat and high-salt’ dietary pattern**  Late-childhood exposed = 0·175  Mid-childhood exposure= -0·066  Early-childhood exposure= -0·074  Fetal exposure group= 0·539  **Western’ dietary pattern**  Late-childhood exposed = -0·075  Mid-childhood exposure= 0·044  Early-childhood exposure= -0·027  Fetal exposure group= 0·003 | **BMI, Mean (SD)**  Did not experience = 23.46 (3.25)  Experienced famine = 23.83 (3.31)  **Whole body % fat**  Did not experience = 29.51 (7.16)  Experienced famine = 29.23 (7.19)  **Average height (cm)**  Did not experience = 157.37 (8.19)  Experienced famine = 156.96 (8.24)  **Systolic Blood Pressure**  Did not experience = 142.88 (19.03)  Experienced famine = 142.44 (19.24)  **Diastolic blood pressure**  Did not experience = 77.92 (9.14)  Experienced famine = 142.44 (19.24)  **Diabetes mellitus**  Did not experience = 14.64%  Experienced famine = 14.36%  **Osteoporosis**  Did not experience = 28.48%  Experienced famine = 29.03%  **Stroke**  Did not experience = 3.91%  Experienced famine = 4.64%  **Hypertension**  Did not experience = 42.38%  Experienced famine = 42.57%  **Myocardial infraction**  Did not experience = 8.34%  Experienced famine = 10.94%  **Angina (chest pain)**  Did not experience = 7.48%  Experienced famine = 9.18%  **Congestive heart failure**  Did not experience = 3.91%  Experienced famine = 3.56%  **Chronic obstructive pulmonary disease**  Did not experience = 8.15%  Experienced famine = 8.46%  **Cataracts**  Did not experience = 40.00%  Experienced famine = 39.33%  **Arthritis**  Did not experience = 20.40%  Experienced famine = 24.03%  **Back Pain**  Did not experience = 45.36%  Experienced famine = 50.45%  **Peripheral arterial disease (ABI<0.9)**  Did not experience = 6.89%  Experienced famine = 6.49%  **Depression (GDS≥8)**  Did not experience = 8.09%  Experienced famine = 9.72%  **MMSE**  Did not experience = 25.91 (3.50)  Experienced famine = 25.41 (3.82) |
| Effect Measures **(**95%CI OF AOR OR COR OR β-coefficients) | **OR (95% CI), CVD**  Fetal- Exposed = 1.35 (1.20–1.52)  Childhood- Exposed = 1.59 (1.40–1.81)  Adolescent- Exposed = 1.52 (1.27–1.81)  **OR (95% CI), MI**  Fetal- Exposed = 1.59 (1.08–2.35)  Childhood- Exposed = 2.20 (1.52–3.20)  Adolescent- Exposed = 2.07 (1.28–3.35)  **OR (95% CI), Stroke**  Fetal- Exposed = 1.40 (1.11–1.78)  Childhood- Exposed = 1.82 (1.45–2.28)  Adolescent- Exposed = 1.92 (1.42–2.58)  **OR (95% CI), CHD**  Fetal- Exposed = 1.44 (1.26–1.65)  Childhood- Exposed = 1.80 (1.56–2.09)  Adolescent- Exposed = 1.83 (1.50–2.24) | **PR 95 % CI**  **Diabetes**  Late-childhood exposed = 2·01 (0·84, 4·81)  Mid-childhood exposure= 2·37 (1·05, 5·36)  Early-childhood exposure= 3·13 (1·43, 6·84)  Fetal exposure group= 0·72 (0·16, 3·33)  **Hypertension**  Late-childhood exposed = 1·50 (0·85, 2·62)  Mid-childhood exposure= 1·51 (0·87, 2·59)  Early-childhood exposure= 1·48 (0·86, 2·57)  Fetal exposure group= 0·80 (0·36, 1·80)  **Hypercholesterolaemia**  Late-childhood exposed = 1·05 (0·44, 2·51)  Mid-childhood exposure= 1·61 (0·76, 3·43)  Early-childhood exposure= 2·07 (1·01, 4·25)  Fetal exposure group= 1·92 (0·75, 4·93)  **Hypertriacylglycerolaemi**a  Late-childhood exposed = 2·01 (0·84, 4·81)  Mid-childhood exposure= 2·01 (0·84, 4·81)  Early-childhood exposure= 2·01 (0·84, 4·81)  Fetal exposure group= 2·01 (0·84, 4·81)  **Hypertriacylglycerolaemia**  Late-childhood exposed = 0·50 (0·21, 1·19)  Mid-childhood exposure= 1·19 (0·72 0·35)  Early-childhood exposure= 1·51 (0·81 0·40,)  Fetal exposure group= 1·45 (0·63, 3·34) | OR (95% CI)  **BMI, Mean (SD)**  Experienced famine = 1.12 (1.05, 1.19)  **Whole body % fat**  Experienced famine = 1.02 (0.96, 1.09)  **Average height (cm)**  Experienced famine = 0.87 (0.82, 0.92)  **Systolic Blood Pressure**  Experienced famine = 0.98 (0.92, 1.05)  **Diastolic blood pressure**  Experienced famine = 0.95 (0.89, 1.03)  **Diabetes mellitus**  Experienced famine = 0.98 (0.82, 1.18)  **Osteoporosis**  Experienced famine = 1.11 (0.95, 1.30)  **Stroke**  Experienced famine = 1.19 (0.86, 1.66)  **Hypertension**  Experienced famine = 1.02 (0.89, 1.17)  **Myocardial infraction**  Experienced famine = 1.36 (1.09, 1.71)  **Angina (chest pain)**  Experienced famine = 1.23 (0.97, 1.57)  **Congestive heart failure**  Experienced famine = 0.92 (0.65, 1.30)  **Chronic obstructive pulmonary disease**  Experienced famine = 1.01 (0.80, 1.29)  **Cataracts**  Experienced famine = 1.01 (0.88, 1.16)  **Arthritis**  Experienced famine = 1.24 (1.06, 1.45)  **Back Pain**  Experienced famine = 1.29 (1.13, 1.48)  **Peripheral arterial disease (ABI<0.9)**  Experienced famine = 0.97 (0.74, 1.27)  **Depression (GDS≥8)**  Experienced famine = 1.24 (0.98, 1.56)  **MMSE**  Experienced famine = 0.84 (0.79, 0.89) |
| Comment | Early- life exposure to undernutrition is associated with significantly increased risk of CVD in later life, especially among those who were in the severely affected famine area | Having suffered the Chinese famine in childhood might affect an individual’s dietary habits and health status, and the joint effect between famine and harmful dietary pattern could have serious consequences on later-life health outcomes. | (Liu et al., 2019)undernutrition has some adverse impact on late life health and functional outcomes. After controlling for age and lifestyle factors (smoking, alcohol, and physical activity), participants who had experienced famine were shorter, had a higher BMI, slower walking speed, shorter stride length, higher prevalence of recurrent falls (defined as two or more falls in the previous 12 months), higher prevalence of myocardial infarction, back pain and arthritis, and poorer cognitive function as measured by mean MMSE compared with those who had not experienced famine |
| Adjustment for covariates | age, sex, BMI, educational status, smoking and drinking status, physical activity, famine severity, healthy diet, metabolic syndrome | Adjusted for gender, education level, activity intensity, smoking status, drinking, per capita income, residence place and disease family history factors | sex, age, PASE, smoking, alcohol, income and education |

| **Authors/year** | **(Shi et al., 2013)** | **(Rotar et al., 2015)** |  |
| --- | --- | --- | --- |
| Participant characteristics | **Age during the study**  Fetal-exposed =41-42  Early childhood =44-45  Mid childhood =46-47  Late childhood =48-49  Unexposed = 38-39 | Survivors/Exposed =70.5±2.4 - 70.7±3.1)  Control group = 71.8±4.5years) |  |
| Settings/context | women and men |  |  |
| Famine year/duration |  |  |  |
| Assessment of famine Exposure | Date of birth | Date of birth |  |
| Exposure groups | Non-exposed cohort (Oct 1 1962-Sep 30 1964)  Fetal exposed cohort (Oct 1 1959-Sep 30 1961)  Early child exposed (Oct 1 1956-Sep 30 1958)  Mid childhood exposed (Oct 1 1954-Sep 30 1956)  Late childhood exposed (Oct 1 1952-Sep 30 1954) | Leningrad siege survivors (08/09/1941 Beginning of siege)  Control group (27/01/1943 End of siege) |  |
| Types of studies | Retrospective cohort | Retrospective cohort |  |
| Sources of information |  |  |  |
| Country | China | Leningrad siege |  |
| Sample size | Fetal-exposed = 272  Early childhood = 424  Mid childhood = 405  Late childhood = 468  Unexposed = 438  Total = 2,007 | Survivors/Exposed = 305  Control group = 51  Total = |  |
| Sampling technique |  |  |  |
| Outcome studied | Anemia |  |  |
| Outcome measurement/definition | WHO |  |  |
| Key findings/Proportions/Mean | **Anemia (%)**  Fetal-exposed = 33.8  Early childhood = 28.1  Mid childhood = 28.2  Late childhood = 29.7  Unexposed = 26.0  **Men**  Fetal-exposed = 28.4  Early childhood = 17.9  Mid childhood = 21.8  Late childhood = 23.6  Unexposed = 16.4  **Women**  Fetal-exposed = 37.4  Early childhood = 37.2  Mid childhood = 32.9  Late childhood = 35.1  Unexposed = 34.2  **Hypertension (%)**  Fetal-exposed = 21.3  Early childhood = 21.5  Mid childhood = 26.4  Late childhood = 29.1  Unexposed = 17.8  **Height (cm)**  Fetal-exposed = 160.8(7.3)  Early childhood = 160.1(7.8)  Mid childhood = 160.1(7.8)  Late childhood = 160.4(7.7)  Unexposed = 162.2(8.0)  **BMI (kg/m2)**  Fetal-exposed = 24.4(3.3)  Early childhood = 23.9(3.4)  Mid childhood = 24.9(17.1)  Late childhood = 23.9(3.1)  Unexposed = 23.8(3.3)  **Overweight (BMI≥24 kg/m2) (%)**  Fetal-exposed = 49.3  Early childhood = 43.6  Mid childhood = 46.3  Late childhood = 45.3  Unexposed = 42.7 | **Myocardial infarction**  Exposed = 10.6%  Control = 11.7%  **Stroke**  Exposed = 8.6%  Control = 5.8%  **Height (m, mean ± SD**  Exposed = 162.4±8.6  Control = 165.9±8.4  **BMI (kg/m2, mean±SD)**  Exposed = 27.5±5.2  Control = 28.6±4.7  **BMI (kg/m2, mean±SD)**  Exposed = 27.5±5.2  Control = 28.6±4.7  **WC (cm, mean±SD)**  Exposed = 92.6± 13.9  Control = 96.3±15.1  **Abdominal obesity**  Exposed = 67%  Control = 65%  **Obesity**  Exposed = 29.2%  Control = 35.2%  **SBP** (mmHg, mean ± SD)  Exposed = 150.9±25.0  Control = 147.8±22.3  **DBP (mmHg, mean ± SD)**  Exposed = 85.5±12.4  Control = 85.3±12.1  **Hypertension**  Exposed = 89.7%  Control = 86.2%  **Total cholesterol (mmol/l, mean±SD)**  Exposed = 5.9±1.3  Control = 5.7±1.6  **HDL (mmol/l, mean±SD)**  Exposed = 1.44±0.32  Control = 1.29±0.27  **Triglycerides (mmol/l, mean±SD)**  Exposed = 1.36±0.82  Control = 1.52±0.84  **Glucose (mmol/l, mean±SD)**  Exposed = 5.7±1.5  Control = 6.1±2.1  **Diabetes**  Exposed = 19.1%  Control = 17.6% |  |
| Effect Measures **(**95%CI OF AOR OR COR OR β-coefficients) | **RR (95% CI): Anemia**  Fetal-exposed = 1.37(1.09-1.71)  Early childhood = 1.04(0.84-1.30)  Mid childhood = 1.03(0.82-1.29)  Late childhood = 1.05(0.84-1.30)  **Men**  Fetal-exposed = 1.87(1.21-2.87)  Early childhood = 1.02(0.66-1.60)  Mid childhood = 1.19(0.76-1.86)  Late childhood = 1.17(0.76-1.79)  **Women**  Fetal-exposed = 1.17(0.90-1.53)  Early childhood = 1.06(0.82-1.36)  Mid childhood = 0.95(0.73-1.24)  Late childhood = 0.98(0.76-1.26) |  |  |
| Comment | Fetal exposure to the Chinese famine was associated with an increased risk of anaemia in adulthood | Survivors had lower anthropometric parameters (height, weight, and BMI) and higher high-density lipoprotein level. There were no significant differences in the prevalence of cardiovascular diseases and target organ damage between groups. Exposure to famine in childhood and intrauterine period of life was associated with a higher prevalence of hypertension and shorter telomere length. Early-life famine, especially started in the intrauterine period and late childhood, may contribute to accelerated aging with telomere shortening in both sexes, but has no direct effect on the prevalence of cardiovascular diseases and risk factors after seven decades since exposure. |  |
| Adjustment for covariates | Income, education, residence (urban/rural), smoking, alcohol drinking, job, hypertension, gender BMI |  |  |

| **Authors/year** | **(Wang et al., 2019a)** | **(Yarde et al., 2013)** |  |
| --- | --- | --- | --- |
| Participant characteristics | **Birth year (range)**  Non-exposed = 1962-1964  Fetal exposed= 1959-1961  Childhood exposed = 1956-1958 | **Age at interview**  Famine exposed = 58.7±0.44  Time control = 58.7±1.6  Sibling control = 56.9±6.4  men and women born around the time of the Dutch famine of 1944–1945, mean age of 59 years. |  |
| Settings/context | women and men | women and men |  |
| Famine year/duration |  |  |  |
| Assessment of famine Exposure | Date of birth | official ration of ,900 kcal/ day between 26 November 1944 and 12 May 1945 |  |
| Exposure groups | Non-exposed (1963–1974)  Fetal- Exposed Cohorts (1959–1962)  Childhood- Exposed Cohorts (1949–1958)  Adolescent- Exposed Cohorts = (1941–1948) | Non-exposed = 381  Late-childhood exposed = 141  Mid-childhood exposure= 173  Early-childhood exposure= 160  Fetal exposure group= 84 |  |
| Types of studies | Retrospective cohort | cohort study |  |
| Sources of information | Risk Evaluation of Cancers in Chinese Diabetic Individuals | birth records of the Amsterdam midwifery school (1948–1957) and the University of Amsterdam Obstetrics Department (1931–1965 |  |
| Country | China | Dutch |  |
| Sample size | Non-exposed = 1,088  Fetal exposed= 751  Childhood exposed = 1,029 | Famine exposed = 407  Time control = 344  Sibling control = 319 |  |
| Sampling technique | multistage random cluster |  |  |
| Outcome studied | Reproductive aging | Reproductive performance and age at menopause |  |
| Outcome measurement/definition | self- reported | Self-Report |  |
| Key findings/Proportions/Mean | **Menarche ages, y**  Non-exposed = 15.90 ± 1.48  Fetal exposed= 15.84 ± 1.59  Childhood exposed = 15.91 ± 1.57  **Premature ovary failure, (%)**  Non-exposed = 1.2%  Fetal exposed= 2.3%  Childhood exposed = 1.1%  **Early menopause, (%)**  Non-exposed = 4.8%  Fetal exposed= 7.3%  Childhood exposed = 6.4%  **Body mass index, kg/m2**  Non-exposed = 24.14±6.09  Fetal exposed= 24.37±3.47  Childhood exposed = 24.20±3.25 | **Natural menopause (%)**  Famine exposed = 74  Time control = 61  Sibling control = 61  **Nulliparity (%), female**  Famine exposed = 9  Time control = 9  Sibling control = 13  **Nulliparity (%), male**  Famine exposed = 11  Time control = 15  Sibling control = 14  Difficulties conceiving (%) **(%), female**  Famine exposed = 19  Time control = 20  Sibling control = 22  Difficulties conceiving (%), **male**  Famine exposed = 16.0  Time control = 17  Sibling control = 15 |  |
| Effect Measures **(**95%CI OF AOR OR COR OR β-coefficients) | **OR (95% CI), Premature ovarian failure**  Fetal exposed= 1.94 (0.93, 4.00)  Childhood exposed = 0.88 (0.39, 1.98)  **OR (95% CI), Early menopause**  Fetal exposed= 1.59 (1.07, 2.36)  Childhood exposed = 1.59 (1.07, 2.36) | **OR with 95% CI, Nulliparity**  **Prenatal Exposed =** 0.95 (0.53, 1.69)  **OR with 95% CI,** **Difficulties conceiving**  Prenatal Exposed **=** 0.78 (0.47, 1.28) |  |
| Comment | Our study showed that fetal exposure to famine was associated with an increased risk of early menopause. Such findings provided evidence in favor of the thrifty phenotype theory in reproductive aging and helped better understand the etiology of early menopause. | No association was found between intrauterine famine exposure and reproductive performance, but survival analysis showed that women exposed in utero were 24% more likely to experience menopause at any age. Gestational famine exposure was not associated with null parity, age at birth of first child, difficulties conceiving or pregnancy outcome in men or women. At any given age, women were more likely to experience menopause after gestational exposure to famine. The association was not attenuated with an additional control for a woman’s birthweight. In this study, there was no association between birthweight and age at menopause after adjustment for gestational famine exposure. |  |
| Adjustment for covariates | socioeconomic status, lifestyle factors, and body mass index | age and smoking status |  |
