# Supplementary material for: Early life famine exposure and anthropometric profile in adulthood: a systematic review and Meta-analysis
Source: BMC Nutr. 2022 Apr 22;8:36. doi: 10.1186/s40795-022-00523-w (PMC9028079; doi:10.1186/s40795-022-00523-w)
Supplement: Supplementary file 2 — Additional file 2. [file 40795_2022_523_MOESM2_ESM.docx]

Supplementary Table 2: The Newcastle-Ottawa Scale (NOS) for assessing the quality of studies in systematic review and meta-analyses, 2021

| Studies | Selection | | | | Comparability | | Exposure | | Total quality score |
| --- | --- | --- | --- | --- | --- | --- | --- | --- | --- |
| Author, year | Is the Case Definition Adequate? | Representativeness of the Cases | Selection of Controls | Definition of Controls | Comparability of cases and controls | Ascertainment of exposure | Same method of ascertainment for cases and controls | Non Response rate |  |
| de Rooij et al., 2007 | 1 | 1 | 1 | 1 | 0 | 0 | 1 | 1 | 6 |
| Han and Hon, 2019 | 1 | 1 | 1 | 1 | 1 | 0 | 1 | 1 | 7 |
| Ning et al., 2019 | 1 | 1 | 1 | 1 | 1 | 1 | 1 | 1 | 8 |
| Wang et al., 2017 | 1 | 0 | 1 | 1 | 1 | 1 | 1 | 1 | 7 |
| Wang et al., 2019 | 1 | 1 | 1 | 1 | 1 | 1 | 1 | 1 | 8 |
| Keinan-Boker et al. 2015 | 1 | 1 | 1 | 0 | 0 | 1 | 1 | 1 | 6 |
| Stanner et al., 1997 | 1 | 1 | 1 | 0 | 1 | 1 | 1 | 1 | 7 |
| Shi, Nicholls et al. 2018 | 1 | 1 | 1 | 1 |  | 1 | 0 | 1 | 8 |
| Chen et al., 2019 | 1 | 1 | 1 | 0 | 0 | 1 | 1 | 1 | 6 |
| Hult et al., 2010 | 1 | 1 | 1 | 1 | 1 | 0 | 0 | 1 | 6 |
| Painter et al., 2006b | 1 | 1 | 1 | 1 | 0 | 0 | 1 | 1 | 6 |
| Chang et al., 2018 | 1 | 1 | 1 | 1 | 1 | 1 | 0 | 1 | 7 |
| Liu et al., 2017a | 1 | 1 | 1 | 1 | 1 | 1 | 1 | 0 | 7 |
| Liu et al., 2019 | 1 | 1 | 1 | 0 | 0 | 1 | 1 | 1 | 6 |
| Meng et al., 2016 | 1 | 1 | 1 | 1 | 1 | 0 | 1 | 1 | 7 |
| Portrait et al., 2017 | 1 | 1 | 1 | 0 | 0 | 1 | 1 | 1 | 6 |
| Ravelli et al., 1999 | 1 | 1 | 1 | 1 | 1 | 1 | 1 | 1 | 8 |
| Song et al., 2020 | 1 | 1 | 1 | 1 | 1 | 0 | 0 | 1 | 6 |
| Stein et al., 2007 | 1 | 1 | 1 | 1 | 1 | 1 | 1 | 1 | 8 |
| van Abeelen et al., 2012c | 1 | 1 | 1 |  | 1 | 1 | 1 | 1 | 8 |
| Wang, Wang et al. 2010 | 1 | 1 | 1 | 1 | 1 | 1 | 0 | 1 | 7 |
| Yang et al., 2008 | 1 | 0 | 0 | 1 |  | 1 | 1 | 1 | 6 |
| Woo et al., 2010 | 1 | 1 | 1 | 1 | 1 | 1 | 1 | 1 | 8 |
