# Supplementary material for: Early life famine exposure and anthropometric profile in adulthood: a systematic review and Meta-analysis
Source: BMC Nutr. 2022 Apr 22;8:36. doi: 10.1186/s40795-022-00523-w (PMC9028079; doi:10.1186/s40795-022-00523-w)
Supplement: Supplementary file 3 — Additional file 3. [file 40795_2022_523_MOESM3_ESM.docx]

**Excluded studies: reference lists**

1. van Abeelen AF, Elias SG, Roseboom TJ, Bossuyt PM, van der Schouw YT, Grobbee DE, et al. Postnatal acute famine and risk of overweight: the dutch hungerwinter study. International journal of pediatrics. 2012;2012.
2. Dobbelsteyn CJ, Joffres MR, MacLean DR, Flowerdew G. A comparative evaluation of waist circumference, waist-to-hip ratio and body mass index as indicators of cardiovascular risk factors. The Canadian Heart Health Surveys. Int J Obes. 2001;25(5):652–61
3. Dercon S, Porter C. Live aid revisited: long-term impacts of the 1984Ethiopian famine on children. J Eur Econ Assoc. 2014;12(4):927–48
4. Ravelli AC, Van Der Meulen JH, Osmond C, Barker DJ, Bleker OP. Obesity atthe age of 50 y in men and women exposed to famine prenatally. Am J Clin Nutr. 1999;70(5):811–6
5. Stanner SA, Bulmer K, Andres C, Lantseva OE, Borodina V, Poteen V, et al. Does malnutrition in utero determine diabetes and coronary heart disease in adulthood? Results from the Leningrad siege study, a cross sectional study. Bmj. 1997;315(7119):1342–8
6. Meng X, Qian N. The long-term consequences of famine on survivors: evidence from a unique natural experiment using China's great famine: National Bureau of Economic Research; 2009. Report No.: 0898–2937
7. Ravelli G-P, Stein ZA, Susser MW. Obesity in young men after famine exposure in utero and early infancy. N Engl J Med. 1976;295(7):349–53
8. Huang C, Li Z, Wang M, Martorell R. Early life exposure to the 1959–1961 Chinese famine has long-term health consequences. J Nutr. 2010;140(10): 1874–8
9. van Abeelen AF, Elias SG, Roseboom TJ, Bossuyt PM, van der Schouw YT, Grobbee DE, et al. Postnatal acute famine and risk of overweight: the dutch hungerwinter study. Int J Pediatr. 2012;2012.
10. Woo J, Leung J, Wong S. Impact of childhood experience of famine on late life health. J Nutr Health Aging. 2010;14(2):91–5
11. Zheng X, Wang Y, Ren W, Luo R, Zhang S, Zhang JH, Zeng Q. Risk of metabolic syndrome in adults exposed to the great Chinese famine during the fetal life and early childhood. Eur J Clin Nutr. 2012;66(2):231–6.
12. Vågerö D, Koupil I, Parfenova N, Sparen P. Long term health consequences following the Siege of Leningrad. Early Life Nutrition and Adult Health and Development. 2013;207:225.
13. Stein AD, Zybert PA, Van der Pal-de Bruin K, Lumey L. Exposure to famine during gestation, size at birth, and blood pressure at age 59 y: evidence from the Dutch Famine. European journal of epidemiology. 2006;21(10):759-65.
