# Supplementary material for: Early life famine exposure and anthropometric profile in adulthood: a systematic review and Meta-analysis
Source: BMC Nutr. 2022 Apr 22;8:36. doi: 10.1186/s40795-022-00523-w (PMC9028079; doi:10.1186/s40795-022-00523-w)
Supplement: Supplementary file 4 — Additional file 4. [file 40795_2022_523_MOESM4_ESM.docx]

a)


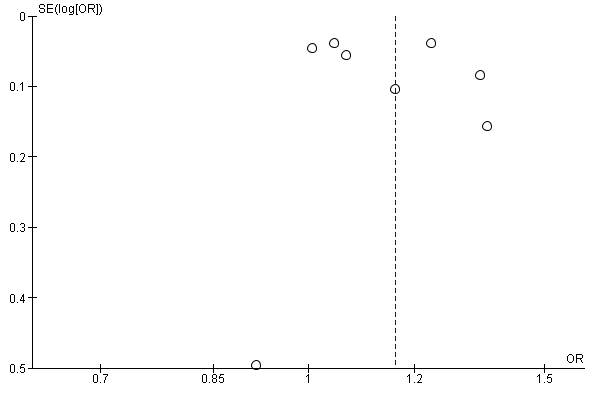


b)


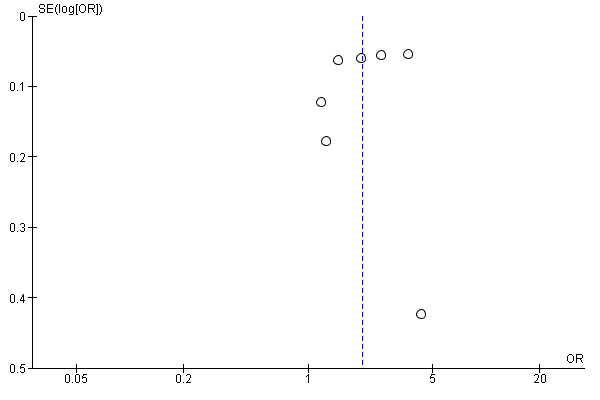


c)


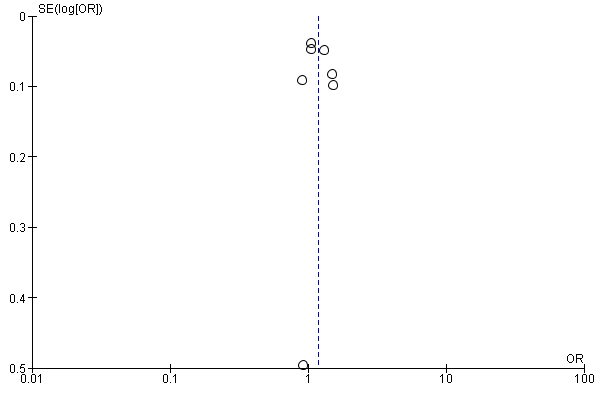


d)


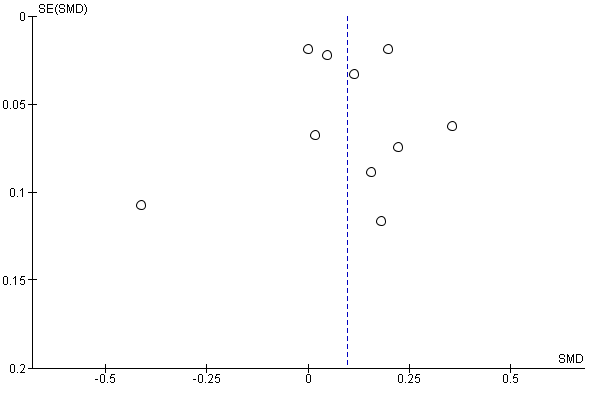


e)


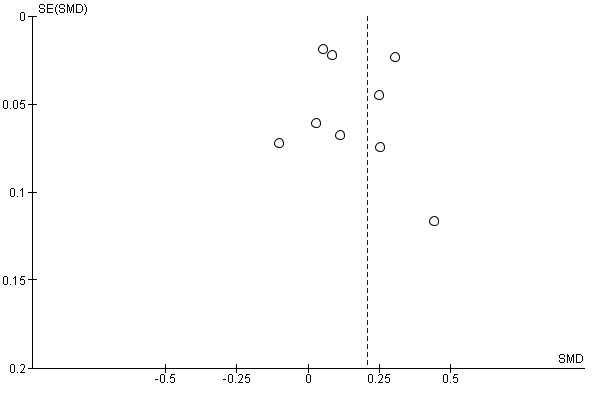


f)


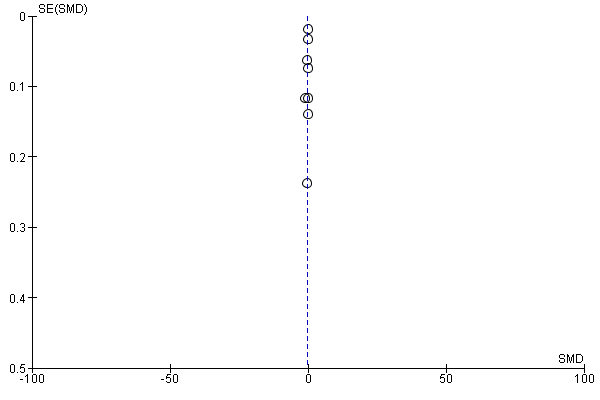


**Supplementary file 4**. Funnel lot for assessment of publication bias, famine exposure in early life and (a) overweight (b) general obesity (c) abdominal obesity (d) BMI (e) waist circumference (f) height in adults, 2021.
